# Missense variant in *TTBK2* kinase domain causes loss of function and impaired protein phosphorylation

**DOI:** 10.1038/s41598-025-32288-0

**Published:** 2025-12-21

**Authors:** Daniela Felício, Hugo Osório, Conceição Pereira, Ana Filipa Brandão, João Parente Freixo, Inês Carvalho, Ana Paula Sousa, Margarida Castro-Caldas, Jorge Sequeiros, Carolina Lemos, Mariana Santos

**Affiliations:** 1 IBMC-Institute for Molecular and Cell Biology, i3S-Instituto de Investigação e Inovação em Saúde, Universidade do Porto, Porto, Portugal; 2 ICBAS School of Medicine and Biomedical Sciences, Universidade do Porto, Porto, Portugal; 3 Ipatimup-Institute of Molecular Pathology and Immunology, Instituto de Investigação e Inovação em Saúde, Universidade do Porto, Porto, Portugal; 4 CGPP-Centre for Predictive and Preventive Genetics, IBMC-Institute for Molecular and Cell Biology, i3S-Instituto de Investigação e Inovação em Saúde, Universidade do Porto, Porto, Portugal; 5 Hospital Dona Estefânia, Centro Hospitalar Universitário de Lisboa Central, Lisboa, Portugal; 6 Centro Hospitalar Universitário de Santo António, ULS de Santo António, Porto, Portugal; 7 UCIBIO, Departamento de Ciências da Vida, Faculdade de Ciências e Tecnologia, Universidade Nova de Lisboa, Caparica, Portugal; 8 Research Institute for Medicines (iMed.ULisboa), Faculty of Pharmacy, Universidade de Lisboa, Lisboa, Portugal; 9 UMIB - Unit for Multidisciplinary Research in Biomedicine, ICBAS School of Medicine and Biomedical Sciences, Universidade do Porto, Porto, Portugal; 10 ITR - Laboratory for Integrative and Translational Research in Population Health, Porto, Portugal; 11 Cambridge Institute for Medical Research, University of Cambridge, Cambridge, UK

**Keywords:** Tau-tubulin kinase 2 (TTBK2), Spinocerebellar ataxia type 11 (SCA11), CRISPR/Cas9, Kinase activity, Phosphoproteomics, Cytoskeleton., Cell biology, Genetics, Molecular biology, Neuroscience

## Abstract

Tau tubulin kinase 2 (TTBK2) is a ubiquitous serine-threonine protein kinase implicated in diverse cellular processes, including microtubule regulation, ciliogenesis, synaptic signaling, and the phosphorylation of key proteins like TDP-43. Despite its relevance, many aspects of TTBK2 function in both physiological and pathological conditions remain poorly understood. Truncating variants in *TTBK2* gene cause **s**pinocerebellar ataxia type 11 (SCA11), a rare form of autosomal dominant cerebellar ataxia. However, the functional consequences and pathogenic potential of missense variants have yet to be elucidated. In this study, we developed a CRISPR/Cas9 knock-in cell model harboring a missense variant in TTBK2 kinase domain (NM_173500.4:c.625 C > T; p.Leu209Phe) to evaluate its impact on TTBK2 expression, associated protein levels, and phosphoproteomic profiles. *TTBK2* missense variant (TTBK2-L209F) was associated with reduced TTBK2 protein levels, altered levels of cytoskeleton-related proteins, and impaired kinase activity, namely toward TDP-43. Phosphoproteomic analyses identified dysregulation in pathways linked to gene regulation, protein degradation, cytoskeletal organization, and TGF-β signaling. These findings provide valuable insights into the biological roles of TTBK2 in cellular signaling. Moreover, this study underscores the importance of functional studies to better understand the consequences of *TTBK2* missense variants, particularly those affecting the kinase domain, and their potential contribution to disease.

**Supplementary Information:**

The online version contains supplementary material available at 10.1038/s41598-025-32288-0.

## Introduction

Tau-tubulin kinase 2 (TTBK2) is a serine-threonine protein kinase that belongs to the casein kinase superfamily. Unlike TTBK1, the other member of theTTBK family, which is neuron-specific, TTBK2 is ubiquitously expressed across multiple tissues . TTBK2 was initially identified due to its ability tophosphorylate the microtubule-associated protein tau and β-tubulin[ ^2,3^] . Since then, TTBK2 has been implicated in various cellular functions, including: ^1^initiation of ciliogenesis, being a key player in the cilium assembly pathway^4,5^ ; (2) microtubule plus-end tracking protein (+TIP), regulating themicrotubule-depolymerizing activity of kinesin family member 2A (KIF2A) ^6,7^;^3^ regulation of neuronal membrane transporters and receptors, importantfor synaptic function and neuronal signaling ; ^4^ phosphorylation of the transactive response DNA-binding protein 43 kDa (TDP-43)^11,12^ whichaccumulates in cytoplasmic inclusions in amyotrophic lateral sclerosis (ALS) and frontotemporal lobar dementia (FTLD-TDP) ; and ^5^ maintenance ofconnectivity and viability of Purkinje cells . Nevertheless, only a few functions have been described in detail, and many aspects of TTBK2 biological rolesare not fully characterized.Pathogenic variants in *TTBK2* gene have been linked to spinocerebellar ataxia type 11 (SCA11), an autosomal dominant disease with adult onset, mainly characterized by slowly progressive cerebellar ataxia. Patients can present with limb and gait imbalance, dysarthria, dysphagia, nystagmus, and hyperreflexia, among other symptoms^14,16^. Life span is thought to be normal, but most patients require a wheelchair decades after disease onset^15,17^. The prevalence of SCA11 is unknown; however, it is extremely rare, likely accounting for less than 1% of autosomal dominant ataxias in Europe^17^.

All described cases of SCA11 have shown small deletions or insertions in *TTBK2*, resulting in frameshifts and truncated proteins^15–19^. Additional studies have reported *TTBK2* missense variants in patients with cerebellar ataxia^20–27^ but, to our knowledge, none has been definitively linked to SCA11^28^. Furthermore, the precise disease mechanism underlying *TTBK2* variants remains poorly understood and is primarily associated with truncating variants. Houlden et al. reported that mutated mRNA is partially degraded by nonsense-mediated decay (NMD)^15^, while other studies showed that truncated TTBK2 exhibits decreased kinase activity^8,9,29^ and may mislocalize from the cytosol to the nucleus^29^. Additionally, truncated TTBK2 appears to dominantly disrupt TTBK2 function, impairing cilia formation and signaling^4,30,31^.

In this study, we investigated the deleterious effects of a missense variant in the kinase domain of *TTBK2* (c.625 C > T; p.Leu209Phe). Functional analyses using a CRISPR/Cas9 knock-in cell model demonstrated a loss of function mechanism for this variant, leading to impaired phosphorylation of both known and new potential TTBK2 targets. Ultimately, these results underscore the importance of investigating the effect of *TTBK2* missense variants, particularly those in the kinase domain.

## Materials and methods

### Variants in Silico analysis

The deleterious effect of *TTBK2* missense variants was accessed by in silico predictions using the Variant Effect Predictor (VEP) tool (Ensembl release 113; https://www.ensembl.org/Tools/VEP), which include the following: MutationTaster, Mutation Assessor, MutPred, FATHMM, FATHMM-MKL, FATHMM-XF, LRT, Deogen2, Eigen/Eigen PC, SIFT, Provean, MVP, Revel, Primate AI, MetaSVM/MetaLR, M-CAP, PolyPhen, LoFtool, Condel, BayesDel (addAF and noAF), ClinPred, LIST-S2, VEST, DANN and CADD. The pathogenic potential of the missense variant was further evaluated resorting to the minor allele frequency in gnomAD (v4.1.0; https://gnomad.broadinstitute.org/), 1000 Genomes Project (Phase 3; https://www.internationalgenome.org/), and dbSNP (build 157; https://www.ncbi.nlm.nih.gov/snp/) databases; and the nucleotide conservation given by PhyloP, PhastCons, Integrated fitCons and GERP++, all from the VEP tool (Ensembl release 113; https://www.ensembl.org/Tools/VEP).

The prediction of protein stability changes of *TTBK2*:c.625 C>T variant (p.Leu209Phe) was accessed through DynaMut^32^ (http://biosig.unimelb.edu.au/dynamut/) and DynaMut2^32^ (http://biosig.unimelb.edu.au/dynamut2/) web servers, obtaining six different predictions of changes in folding free energy (ΔΔG): DynaMut2, DynaMut, ENCoM, mCSM, SDM, and DUET, recurring to the partial or complete 3D structure available in the Protein Data Bank (PDB) (6VRF; https://www.rcsb.org/). The DynaMut2 web server was also used to generate images of interatomic interactions.

### Expression vectors and antibodies

The pEGFP-C1-TTBK2-WT plasmid (MRC PPU, Dundee, Scotland) was modified by site-directed mutagenesis using the QuikChange II kit (Agilent, Santa Clara, CA, USA) to introduce *TTBK2*:c.625 C > T variant. The following primers pairs were used to introduce the variant: forward primer

 5′-GAAATGGGAAGACATGATGACTTTTGGTCCTTATTCTACATGT-3′ 

and reverse primer 5′-ACATGTAGAATAAGGACCAAAAGTCATCATGTCTTCCCATTTC-3′, respectively.

 Antibodies used for Western blot and immunofluorescence studies are described in Table S1.

### Cell culture and CRISPR/Cas9 gene editing

HEK293T (ATCC; kindly provided by Elsa Logarinho, IBMC/i3S, Porto) were grown in DMEM high glucose GlutaMAX™ supplemented with 10% FBS and 1% antibiotic-antimycotic (Gibco, ThermoFisher Scientific, Waltham, MA, USA) at 37 °C in a humidified 5% CO_2_ atmosphere. For overexpression studies, cells were transiently transfected for 24 h with each plasmid using Lipofectamine™ 2000 (Invitrogen, ThermoFisher Scientific, Waltham, MA, USA) according to the manufacturer’s protocol.

HEK293T cells were genetically modified using CRISPR/Cas9 system to insert a N-terminal 3xFLAG-tag in both alleles of *TTBK2* (establishment of FLAG-TTBK2-WT cell line), according to methods already described^34^. A single-chain guide RNA (sgRNA) was selected using the CRISPOR online tool^35^ (v4.97; https://crispor.gi.ucsc.edu/); 5’-ACACATGCATCAGGTTTAGG-3’) and cloned in BbsI (ThermoFisher Scientific, Waltham, MA, USA) restriction sites into the mammalian expression construct pSpCas9(BB)−2 A-Puro (PX459) V2.0 (Addgene, Watertown, MA, USA), bearing both sgRNA scaffold backbone and *S. pyogenes* Cas9. A single-stranded oligodeoxynucleotide (ssODN) with 50 bp homology arms 

(5´-GGGAACTGGATGCCTGTGTAGCTGTTCTACCATATCAGT

GTATTGCAATGGACTACAAAGACCATGA

CGGTGATTATAAAGATCATGACATCGATTACAAGGATGACGATGACA

AGGGTGGTGGTGGATCCAGTGGGGGAGGAGAGCAGCTGG

ATATCCTGAGTGTTGGAATCCTAGTGAA-3´) 

was designed to serve as a template for homology-directed repair (HDR). A silent point mutation within the PAM sequence was introduced in the ssODN. The Cas9/sgRNA (2 µg) plasmid was co-transfected along with the ssODN (10 µM), in the presence of an inhibitor of non-homologous end joining (NHEJ) repair - NU7026^34^ (10 µM; Sigma-Aldrich, Darmstadt, Germany), for 24 h using the DreamFect Gold reagent (OZ Biosciences, Marseille, France), according to the manufacturer’s protocol. Following transfection, clonal cell lines were isolated through fluorescence-activated cell sorting (FACS) and further genotyped by PCR (forward primer 5′-TATTAGTAGTGCTTCCTGATGCTG-3′ and reverse primer 5′- CTCTCTGAGATGTACACTCACCAC-3′), using Ranger Mix (Bioline, London, UK). PCR products with the expected molecular weight were purified with Exo/SAP (GRiSP, Porto, Portugal) and further analysed by Sanger sequencing using BigDye Terminator Cycle Sequencing v1.1 (Applied Biosystems, Foster City, CA, USA) in an ABI 3130xl Genetic Analyzer (Applied Biosystems, Foster City, CA, USA).

Then, homozygous knock-in of *TTBK2*:c.625C > T variant was performed on the FLAG-TTBK2-WT cell line described above (establishment of the FLAG-TTBK2-L209F cell line). Following the same methodology, one sgRNA (5’-ACATGTAGAATAAGGACCAA-3’) was cloned into the pSpCas9(BB)−2 A-Puro (PX459) V2.0. The ssODN with 50 bp homology arms 

(5´-TGTGACTGGATTCTGTTTTGTTTATGAAGGAAATGGGAAGA

CATGATGACTTTTGGTCATTATTCTACATGTTGGTGGAGTTT

GTGGTTGGTCAGCTGCCCTGGAGAAA-3´) contained a silent point mutation to disrupt the AvaII restriction site, allowing genotyping of cell clones; the PAM sequence was disrupted when HDR on the variant site occurred. Transfection and establishment of clonal cells was performed as described above. Cells were genotyped through PCR (forward primer 5′-ACACATTAACTAATGGGCTGTT-3′ and reverse primer 5′-TCTTCTACCACAATTGACATCT-3′) followed by AvaII (New England Biolabs, Ipswich, MA, USA) digestion. Potential hits were sequenced by Sanger sequencing (as described above).

 Off-targets of both sgRNAs were predicted using CRISPOR^35^ (v4.97; https://crispor.gi.ucsc.edu/) and CRISPR Finder^37^ (https://wge.stemcell.sanger.ac.uk) tools and checked for alterations using Sanger sequencing (as described above) in the selected clonal cell lines. Alteration in off-targets with four or fewer mismatches were excluded by sequencing (Table S2). Also, all *TTBK2* exons and flanking intronic regions (Table S3) were checked for potential off-target effects.

### Cell viability, proteasome, and autophagy assays

The CellTiter-Glo^®^ Luminescent Cell Viability Assay (Promega, Madison, WI, USA) was used to determine the number of viable or metabolically active cells in the cell lines created, according to the manufacturer’s protocol. Luminescence was measured using the Synergy Mx Microplate Reader (Agilent, Santa Clara, CA, USA).

The Proteasome-Glo™ Chymotrypsin-Like, Trypsin-Like and Caspase-Like Cell-Based Assays (Promega, Madison, WI, USA) were used to measure the protease activities associated with the proteasome complex in the cell lines, according to the manufacturer’s protocol. Cells were treated with 5 µM MG132 (proteasome inhibitor) for 3 h as a control condition and to help define the activity attributable to the proteasome. The Synergy Mx Microplate Reader (Agilent, Santa Clara, CA, USA) was used to measure luminescence.

To monitor the autophagy flux, the cell lines were incubated with the autophagy inhibitor chloroquine (10 µM for 18 h; Sigma-Aldrich, Darmstadt, Germany) or control vehicle DMEM (Gibco, ThermoFisher Scientific, Waltham, MA, USA). Protein lysates were extracted from cells and the expression of autophagy protein markers was analyzed by Western blotting, as described below.

### qPCR

 Total RNA was isolated from HEK293T, FLAG-TTBK2-WT and -L209F cells, using the NZY Total RNA Isolation kit (Nzytech, Lisbon, Portugal), as per the manufacturer’s recommendations. RNA quantification was performed on the NanoDrop 2000 Spectrophotometer (ThermoFisher Scientific, Waltham, MA, USA). cDNA was synthesized by reverse transcription-PCR of 3 µg total RNA with oligo(dT), using the NZY First-Strand cDNA Synthesis Kit (Nzytech, Lisbon, Portugal), according to the manufacturer’s protocol. qPCR was performed using PowerUp™ SYBR™ Green Master Mix (Applied Biosystems, Foster City, CA, USA), along with respective primers (Table S4). Human *ACTB* was used as endogenous control. All reactions were performed in triplicate and replicated three times, using the Applied Biosystems 7500 Fast Real-Time PCR (Applied Biosystems, Foster City, CA, USA). The analysis was carried out using 7500 Software (version 2.0.6; Applied Biosystems, Foster City, CA, USA), and mRNA expression was calculated by the 2^−ΔΔCT^ method.

### Western blot analysis

HEK293T, FLAG-TTBK2-WT and -L209F cells were collected in RIPA buffer (150 mM NaCl, 1.0% IGEPAL^®^ CA-630, 0.5% sodium deoxycholate, 0.1% SDS, 50 mM Tris, pH 8.0; Sigma-Aldrich, Darmstadt, Germany) supplemented with cOmplete Protease Inhibitor Cocktail (Roche, Basel, Switzerland) and then sonicated for 10 s intermittently with a power of 60 W (Branson sonifier 250, ThermoFisher Scientific, Waltham, MA, USA). Samples analyzed for protein phosphorylation were also supplemented with 1 mM Na_3_VO_4_ and 5 mM NaF. Total protein concentration was measured with the Pierce BCA protein assay kit (ThermoFisher Scientific, Waltham, MA, USA) according to the manufacturer’s instructions. Samples (30–50 µg of total protein) were separated on SDS-PAGE (Mini-PROTEAN; Bio-Rad, Hercules, CA, USA) and electrophoretically transferred onto PVDF membranes (Merck-Milipore, Darmstadt, Germany) using wet (Mini Trans-Blot electrophoretic transfer cell; Bio-Rad, Hercules, CA, USA) or semi-dry (SemiPhor transfer unit; Hoefer, Holliston, MA, USA) transfer systems. Membranes were firstly stained with Ponceau S and then blocked in 5% non-fat dry milk or bovine serum albumin (BSA) in Tris buffer saline/0.1% Tween-20 (TBS-T) for 1 h with agitation, at room temperature (RT), and subsequently incubated with specific primary antibodies as indicated (diluted in 5% BSA in TBS-T and 1% sodium azide), overnight at 4 °C with agitation. The membranes were washed with TBS-T and then incubated with HRP-conjugated secondary antibody (diluted in 3% non-fat dry milk or BSA in TBS-T) for 1 h with agitation at RT. Following washes with TBS-T, detection was achieved using WesternBright Sirius or WesternBright Standard ECL-HRP substrates (Advansta, San Jose, CA, USA), and chemiluminescence detected with a ChemiDoc XRS + Imaging System (Bio-Rad, Hercules, CA, USA). Ponceau S staining (used as loading control) and protein bands were quantified using the ImageLab software (version 6.1; Bio-Rad, Hercules, CA, USA). Quantitative comparisons between samples of each experiment were always performed on the same blot. When necessary, membranes were stripped by incubation in a stripping solution (62.5 mM Tris-HCl pH 6.7, 2% SDS, 100 mM β-mercaptoethanol) at 50 °C for 30 min, with gentle agitation.

### Phosphoprotein enrichment and precipitation

Phosphoprotein enrichment was performed in FLAG-TTBK2-WT and -L209F cells (three independent experiments) using the phosphate metal affinity chromatography columns (TALON^®^ PMAC Phosphoprotein Enrichment Kit; Clontech, Takara Bio, Mountain View, CA, USA), according to the manufacturer’s instructions. The second and third phospho-enriched fractions, which had the highest protein amount, were concentrated using a speed vacuum concentrator (SpeedVac, ThermoFisher Scientific, Waltham, MA, USA) and proteins further precipitated using the ProteoExtract Protein Precipitation Kit (Merck-Millipore, Darmstadt, Germany), according to the manufacturer’s instructions.

### Mass spectrometry analysis

Sample preparation, protein identification, and quantitation, as well as data acquisition and processing, were performed according to the procedure described by Osório et al.^38^. The acquired raw data were processed using the Proteome Discoverer software (version 2.5.0.400; ThermoFisher Scientific, Waltham, MA, USA). Protein identification analysis was performed with the data available in the UniProt protein sequence database for the Reviewed Homo sapiens Proteome 2021_03 with 20,371 entries and a common contaminant database from MaxQuant (version 1.6.2.6, Max Planck Institute of Biochemistry). Additionally, phosphorylation of serine (Ser), threonine (Thr), and tyrosine (Tyr) residues was defined as variable modification and the ptmRS node was used for analysis and mapping of phosphorylation sites. Mass spectrometry data have been deposited to the ProteomeXchange Consortium via PRIDE^39^ repository with the identifier PXD056662.

### Phosphoprotein network and functional enrichment analysis

Phosphoproteins were considered for the analysis of differentially expressed proteins (DEP) if detected in 2 out of 3 samples per experimental group with at least 50% of samples with protein-related peptides sequenced by MS/MS. Proteins identified with more than two unique peptides or with only one unique peptide but an MW lower than 30 kDa were included for analysis. For the selection of upregulated and downregulated proteins the L209F/WT ratio was set at ≥ 1.60 and ≤ 0.625, respectively. p-value was adjusted using Benjamini-Hochberg correction (FDR ≤ 0.05).

The protein-protein interaction (PPI) network from the significant DEP list (UniProt accession numbers) was achieved using the StringAPP plugin version 1.7.0 (minimum confidence 0.4) in the Cytoscape software^40^ (version 3.9.1; https://cytoscape.org/). Further analysis was performed using the NetworkAnalyzer plugin version 4.4.8. In the Cytoscape software, the nodes were labeled according to the relative expression [log2 (L209/WT ratio) values] from red/upregulated to blue/downregulated. The node size and edge width were matched to the node degree of distribution and STRING score retrieved to highlight the most relevant and confident interactions inside the network. The AutoAnnotate plugin version 1.3.5 allowed to cluster (GLay algorithm, clusterMaker2 v2.2) the protein-protein interactions and annotate them according to the word frequencies of Gene Ontology (GO) terms from the Biological Process category (previously attributed in the Proteome Discoverer software). The Over Representation Analysis (ORA) and Gene Set Enrichment Analysis (GSEA) methods were performed with the WebGestalt tool (2019 version; https://www.webgestalt.org/) according to default parameters. The weighted set cover redundancy reduction method was included as a post-processing step, allowing to identify the most representative and significant sets.

The phosphorylation sites identified in the DEP were analyzed using PhosphositePlus (v6.7.1.1; https://www.phosphosite.org/), Phospho.ELM (v.9.0; http://phospho.elm.eu.org/index.html), and PhosphoNet (2019 version; http://www.phosphonet.ca/) databases.

### Statistical analysis

All quantitative data are expressed as mean ± standard deviation (SD) of at least three independent experiments. Statistical significance analysis was conducted using one-way analysis of variance (ANOVA) with Tukey’s post-hoc tests, when comparing means of more than two groups, or a two-tailed Student’s t-test to compare means between two groups. Non-parametric tests (Mann-Whitney U) were used when normality (Shapiro-Wilk test) and/or homogeneity of the variances (Levene test) were not observed. The level of statistical significance was set at a p-value ≤ 0.05. The statistical analysis was performed using the IBM SPSS Statistics software (version 25.0; IBM, Armonk, NY, EUA).

## Results

### In Silico analysis of *TTBK2* missense variants

**Fig. 1:**
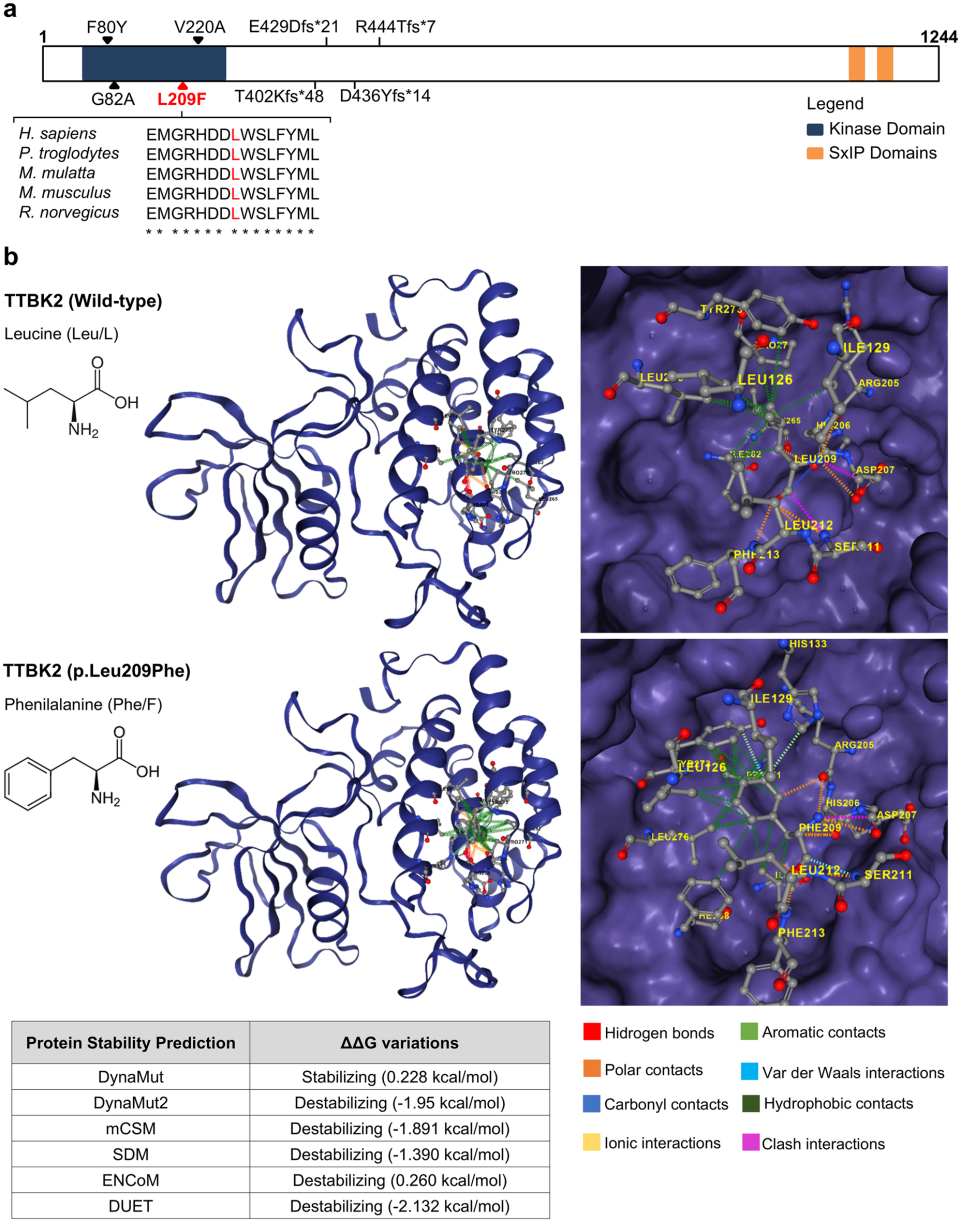
*TTBK2* variants associated with cerebellar ataxia. **(a)** Schematic representation of the TTBK2 protein showing the localization of the missense variant of this study in red (L209F), and truncating and missense variants identified in other studies depicted in black. Sequence alignment of the flanking residues of Leu 209 of human TTBK2 kinase domain against other species is also shown (using Clustal Omega program). **(b)** Protein models of TTBK2 showing altered residue interactions upon amino acid change leucine to phenylalanine in position 209. On the left, the entire kinase domain is shown; on the right, a close-up view highlights the Leu209/Phe209 residues at the center, displaying their interactions with nearby residues in different colors. The models were performed in DynaMut2 (http://biosig.unimelb.edu.au/dynamut2/; PDB: 6VRF). A table displaying the protein stability prediction scores is also shown.

 Truncating variants have been definitively linked to SCA11, whereas missense variants (Fig. 1a) have been identified in patients with cerebellar ataxia, but their pathogenicity has not been clearly established. Missense variants in the conserved kinase domain of TTBK2 have the potential to disrupt its activity and function. To further explore this, we conducted an in silico analysis of previously reported missense variants within TTBK2 kinase domain (Table S5). Among these, a novel variant (NM_173500.4:c.625 C > T; p.Leu209Phe) identified in a Portuguese family with progressive cerebellar ataxia^41^ (Supplementary material and methods), was notable for pathogenicity potential across multiple software tools and a higher CADD score. This variant has only three heterozygous allele counts and a total minor allele frequency of 1,859 × 10^− 06^ according to the gnomAD v4.1.0 database. It is reported in dbSNP as rs1595936604 and classified in ClinVar (VCV001978434.2) as a variant of unknown significance (one submission).

This TTBK2 variant, hereafter referred to as L209F, affects a highly conserved amino acid (Fig. 1a) located within an alpha helix of the kinase domain. It results in the substitution of leucine in position 209 by a phenylalanine, a residue buried within the core of the kinase domain. Protein stability analysis predicted a destabilizing effect of this missense variant (Fig. 1b), namely an increase in aromatic and hydrophobic contacts with the neighboring residues.

### TTBK2-L209F affects protein expression

**Fig. 2:**
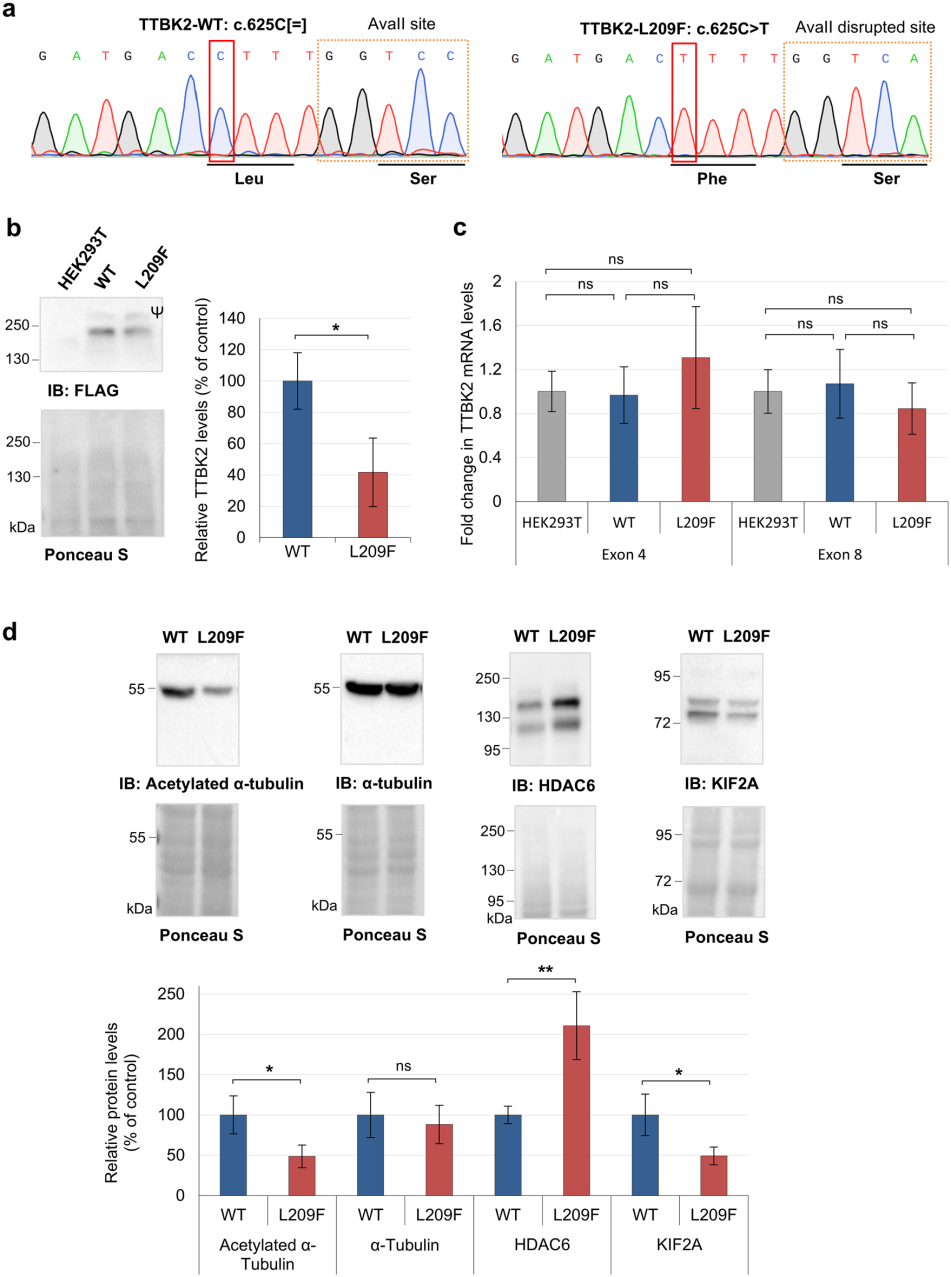
The TTBK2-L209F variant caused a reduction in TTBK2, α-tubulin acetylation and KIF2A levels. **(a)** Electropherogram showing the variant TTBK2:c.625 C > T (boxed in red) introduced by CRISPR-Cas9 in the FLAG-TTBK2-L209F cell line. These cells also carry a single silent point mutation that disrupts AvaII restriction site, enabling initial screening of cell clones. Electropherogram of the control cell line (TTBK2-WT) is also shown for comparison. **(b)** Analysis of TTBK2 protein expression in HEK293T, FLAG-TTBK2-WT (WT) and FLAG-TTBK2-L209F (L209F) cells by immunoblotting with an anti-FLAG antibody. Ponceau S staining was used as loading control. Ψ identifies an unspecific band. Quantification data is presented in percentage as mean ± SD of three independent experiments; *P ≤ 0.05 compared with WT cell line (t-test). **(c)** Analysis of TTBK2 mRNA levels by qPCR. Primers targeting exon 4 or exon 8 of *TTBK2* were used. Quantification data are presented in fold change as mean ± SD of three independent experiments; non-significant (ns) compared with HEK293T (non-modified cell line; one-way ANOVA/Tukey). **(d)** Analysis of acetylated α-tubulin, α-tubulin, HDAC6, and KIF2A expression levels in WT and L209F cells by immunoblotting with specific antibodies. Ponceau S staining was used as the loading control. Quantification data are presented in percentage as mean ± SD of at least three independent experiments; non-significant (ns), *P ≤ 0.05 and **P ≤ 0.01 (t-test).

To study the pathogenic mechanisms triggered by L209F, we first created an endogenous HEK293T knock-in cell model expressing *TTBK2* in fusion with an N-terminal 3xFLAG-tag (FLAG-TTBK2-WT cell line) through CRISPR/Cas9 editing. The introduction of the 3xFLAG allowed the detection of TTBK2 using specific FLAG-tag antibodies, overcoming the limitations of the commercial options. Next, we introduced the L209F variant into the FLAG-tagged cell line (FLAG-TTBK2-L209F cell line), using the same methodology (Fig. 2a). The newly established FLAG-TTBK2-L209F and the control FLAG-TTBK2-WT CRISPR/Cas9-modified cell lines (Fig. 2a) were then used for subsequent experiments.

Expression analysis revealed a significant 60% decrease (p *=* 0.023) in TTBK2 protein levels in the FLAG-TTBK2-L209F cell line compared to FLAG-TTBK2-WT cells (Fig. 2b). qPCR showed no significant reduction of TTBK2 mRNA levels in FLAG-TTBK2-L209F cells, indicating that the variant does not cause mRNA reduction or instability (Fig. 2c). Thus, TTBK2-L209F reduced levels may result from reduced protein stability, as supported by in silico predictions (Fig. 1b). In addition, we showed that introducing the 3xFLAG-tag did not affect TTBK2 mRNA expression, as shown by comparable mRNA levels between HEK293T and FLAG-TTBK2-WT cells (Fig. 2c).

To understand if L209F affects cell viability and/or induces apoptosis, we performed a cell viability assay and analyzed caspase-3 expression and cleavage. We found no significant changes in both assays between the cell lines (Fig. S1). Also, no cleaved fragments of caspase-3 were detected, indicating no measurable activation of apoptosis (Fig. S1b).

Redistribution of TTBK2 truncating proteins to the nucleus has been previously reported^29^. To address this, we performed fluorescence microscopy and subcellular fractionation to determine TTBK2-L209F subcellular localization. In both cell lines, TTBK2 seemed to be distributed throughout the cytoplasm, without noticeable changes in the localization of TTBK2-L209F compared to TTBK2-WT (Fig. S2; Supplementary material and methods).

### TTBK2-L209F influences microtubule stability

TTBK2 was reported to participate in microtubule regulation by associating with tau, tubulin and KIF2A^2,3,7^, and by acting as a + TIP^6,7^. Therefore, we started by analyzing the expression of acetylated α-tubulin, an indicator of microtubule stability. Immunoblotting analysis showed that acetylated α-tubulin levels were significantly reduced by approximately 50% (*p* = 0.031) in FLAG-TTBK2-L209F cells compared to WT cells, which was not a consequence of alterations in total α-tubulin levels (Fig. 2d). Reversible deacetylation of α-tubulin is mediated by histone deacetylases, namely HDAC6^34^. Thus, we measured HDAC6 levels and found that FLAG-TTBK2-L209F cells display nearly twice as much HDAC6 compared to WT cells (*p* = 0.004; Fig. 2d), in agreement with reduced α-tubulin acetylation. Furthermore, KIF2A was expressed at lower levels in FLAG-TTBK2-L209F cells (around 50% less than WT cells; *p* = 0.035; Fig. 2d), even though KIF2A mRNA levels remained unchanged (Fig. S3), confirming that KIF2A transcription is not affected but rather protein expression or stability. These results indicate dysregulation of TTBK2 cytoskeletal targets, likely resulting in microtubule instability in FLAG-TBK2-L209F cells.

### TTBK2-L209F impairs kinase activity against TDP-43

**Fig. 3:**
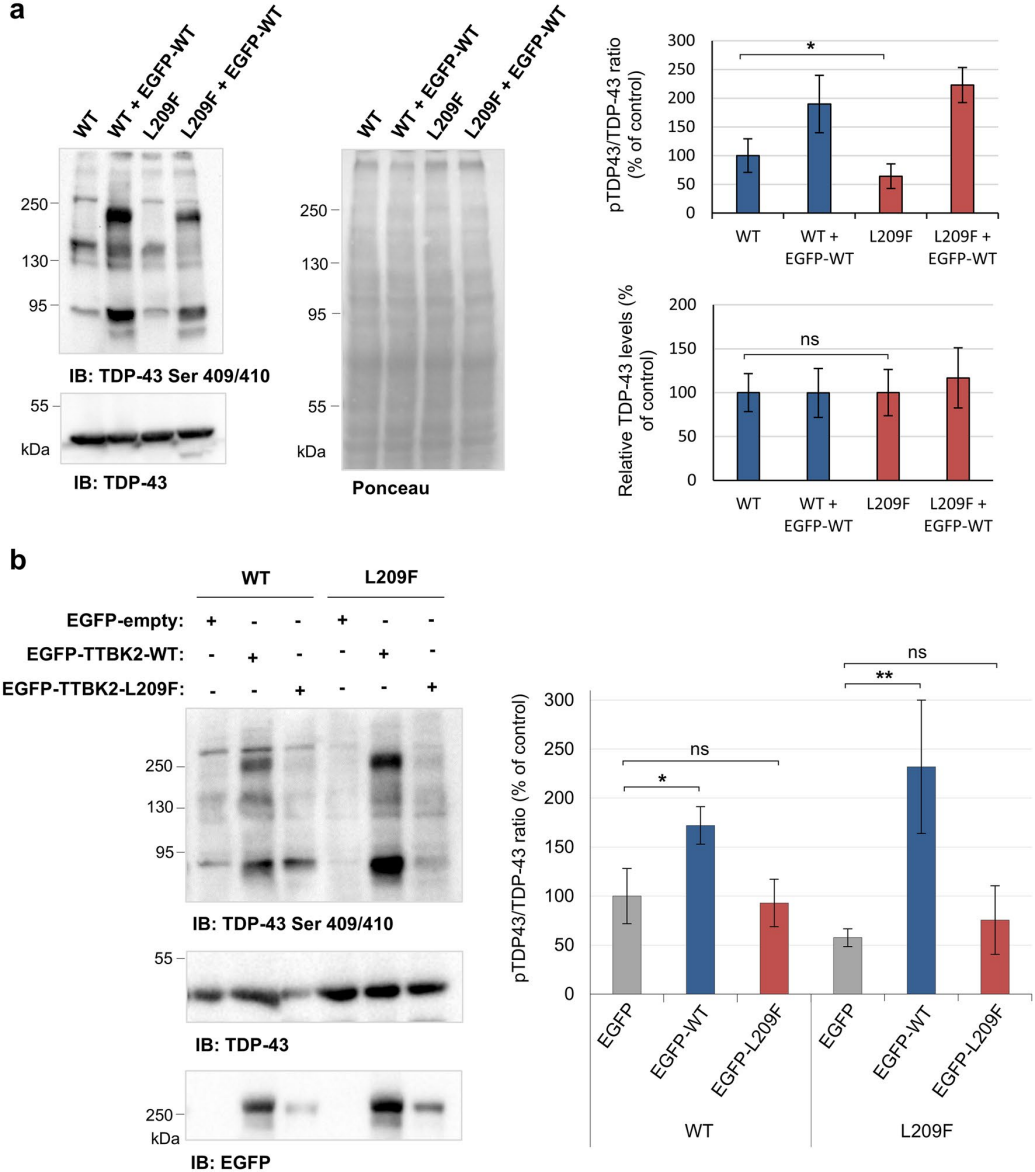
TTBK2-L209F variant impaired TDP-43 phosphorylation. **(a)** Analysis of TDP-43 Ser409/410 protein levels (pTDP-43) in FLAG-TTBK2-WT (WT) and FLAG-TTBK2-L209F (L209F) cells. Total TDP-43 was used to normalize pTDP-43 levels, while Ponceau S was used to normalize total TDP-43 levels. **(b)** WT and L209F cells were transfected with the empty vector EGFP, EGFP-TTBK2-WT or EGFP-TTBK2-L209F, followed by immunoblotting with anti-TDP-43 Ser409/410, anti-TDP-43 and anti-EGFP antibodies. Total TDP-43 levels were used to normalize pTDP-43 levels. Quantification data are presented in percentage as mean ± SD of three independent experiments; non-significant (ns), *P ≤ 0.05 and **P ≤ 0.01 compared with the WT cell line (Mann-Whitney U test in (a) and one-way ANOVA/Tukey in (b)).

TDP-43 is a well-known target of TTBK2 protein kinase^11^. To investigate the effect of the L209F variant on TTBK2 kinase activity against TDP-43, we analyzed the phosphorylation state of TDP-43 at Ser409/410 in our cell lines. In FLAG-TTBK2-L209F cells, the levels of phosphorylated TDP-43 (pTDP-43; bands above 74 kDa) were significantly reduced by 36% (*p* = 0.041; Fig. 3a), while total TDP-43 levels remained unaffected. Transfection of cells with EGFP-TTBK2-WT confirmed that TTBK2 can augment pTDP-43 levels (Fig. 3a), in agreement with previous results. Moreover, we showed that pTDP-43 levels increased with overexpression of EGFP-TTBK2-WT in both cell lines (around 70% and 170%, *p* = 0.025 and 0.007, respectively; Fig. 3b) but not following transfection with EGFP-TTBK2-L209F. Similar to the effects observed with EGFP-TTBK2-L209F overexpression, a kinase-dead and a SCA11 (R444fs*5) mutant did not alter pTDP-43 levels in HEK293 cells. In contrast, the F287S mutant (variant outside the kinase domain) led to an increase in pTDP-43 levels (Fig. S4). These results support that TTBK2-L209F has impaired kinase activity toward TDP-43.

### TTBK2-L209F is linked to abnormal protein phosphorylation

Phosphoprotein enrichment followed by MS/MS analysis was performed to identify and quantify phosphorylated proteins (Fig. S5a), in the context of TTBK2 impaired kinase activity. After filtering the data, we identified 4429 proteins amongst the phospho-enriched fractions of six independent samples: three of FLAG-TTBK2-WT cells (control condition) and three of FLAG-TTBK2-L209F cells (impaired kinase activity). Using a threshold of L209/WT ratio ≥ 1.60 for upregulated proteins and ≤ 0.625 for downregulated proteins, a total of 110 differentially expressed phosphoproteins (DEPs) were identified: 50 upregulated and 60 downregulated in FLAG-TTBK2-L209F cells (Table 1; Fig.S5b).In addition, eighteen phosphorylation sites across ten DEP (Table 2) were also identified. These include AMOT, HTATSF1, RBM20, SQSTM1 (from nowon referred as p62), SMAD2, CTIF, NEDD4L, TNIK, IRS4, and BAG3. Using publicly available databases, we found that half of the identifiedphosphorylated residues had also been found in other mammalian studies. Additionally, the kinases responsible for the phosphorylation of these residueshave previously been described. For example, p62 [Ser272] is phosphorylated by cyclin-dependent kinase 1 (CDK1) and mitogen-activated protein kinase(MAPK) 13; SMAD2 [Thr8] by MAPK3; and NEDD4L [Ser448] by protein kinase cAMP-activated catalytic subunit alpha (PKACA) andserum/glucocorticoid regulated kinase 1 (SGK1) (Table 2).

**Table 1:** List of differentially expressed proteins (DEPs) between FLAG-TTBK2-L209 and -WT cells. In total, 50 proteins were upregulated and 60 downregulated in the FLAG-TTBK2-L209F cell line. P≤0.05 was considered with adjustment using the Benjamini–Hochberg correction (FDR≤0.05). The accession numbers are from the UniProt database.

**Protein**	**Accession No.**	**Gene Symbol**	**Fold-Change (Mut/WT)**	**Unique Peptides**	**MW [kDa]**
HAUS augmin-like complex subunit 2	Q9NVX0	*HAUS2*	100	1	26.9
Annexin A1	P04083	*ANXA1*	100	2	38.7
Forkhead box protein M1	Q08050	*FOXM1*	100	2	84.2
Serine/threonine-protein kinase ULK3	Q6PHR2	*ULK3*	100	2	53.4
Ubiquitin-associated domain-containing protein 1	Q9BSL1	*UBAC1*	100	2	45.3
Vesicle-trafficking protein SEC22b	O75396	*SEC22B*	8.689	1	24.6
Uncharacterized protein C18orf25	Q96B23	*C18orf25*	2.586	6	43.4
Phosphatidylinositol 3-kinase regulatory subunit gamma	Q92569	*PIK3R3*	2.472	3	54.4
NAD-dependent malic enzyme, mitochondrial	P23368	*ME2*	2.412	22	65.4
Oligoribonuclease, mitochondrial	Q9Y3B8	*REXO2*	2.382	3	26.8
Bardet-Biedl syndrome 7 protein	Q8IWZ6	*BBS7*	2.366	2	80.3
Keratin, type I cytoskeletal 9	P35527	*KRT9*	2.282	32	62
Acyl-CoA dehydrogenase family member 11	Q709F0	*ACAD11*	2.223	13	87.2
Ceroid-lipofuscinosis neuronal protein 5	O75503	*CLN5*	2.209	2	41.5
Neuromodulin	P17677	*GAP43*	2.208	1	24.8
Mothers against decapentaplegic homolog 4	Q13485	*SMAD4*	2.196	17	60.4
AT-rich interactive domain-containing protein 5B	Q14865	*ARID5B*	2.165	3	132.3
Mucosa-associated lymphoid tissue lymphoma translocation protein 1	Q9UDY8	*MALT1*	2.08	3	92.2
E3 ubiquitin-protein ligase Midline-1	O15344	*MID1*	2.062	6	75.2
Insulin receptor substrate 4	O14654	*IRS4*	2.034	39	133.7
Histone H3.3	P84243	*H3-3A; H3-3B*	2.022	1	15.3
Asparagine--tRNA ligase, cytoplasmic	O43776	*NARS1*	1.993	18	62.9
Thioredoxin reductase 1, cytoplasmic	Q16881	*TXNRD1*	1.984	3	70.9
Slit homolog 2 protein	O94813	*SLIT2*	1.983	4	169.8
Angiomotin	Q4VCS5	*AMOT*	1.978	45	118
Profilin-2	P35080	*PFN2*	1.957	1	15
CBP80/20-dependent translation initiation factor	O43310	*CTIF*	1.951	8	67.5
Protein ERGIC-53	P49257	*LMAN1*	1.927	16	57.5
Carboxymethylenebutenolidase homolog	Q96DG6	*CMBL*	1.91	1	28
Methyl-CpG-binding domain protein 1	Q9UIS9	*MBD1*	1.895	6	66.6
Angiomotin-like protein 2	Q9Y2J4	*AMOTL2*	1.825	5	85.7
Keratin, type I cytoskeletal 10	P13645	*KRT10*	1.805	27	58.8
Histone-lysine N-methyltransferase MECOM	Q03112	*MECOM*	1.796	10	138
E3 ubiquitin-protein ligase NEDD4-like	Q96PU5	*NEDD4L*	1.772	13	111.9
WD repeat-containing protein 74	Q6RFH5	*WDR74*	1.764	10	42.4
SH2 domain-containing adapter protein B	Q15464	*SHB*	1.755	4	55
Histone H3.2	Q71DI3	*H3C13; H3C14; H3C15*	1.739	1	15.4
Keratin, type II cytoskeletal 1	P04264	*KRT1*	1.739	36	66
Sequestosome-1	Q13501	*SQSTM1*	1.717	9	47.7
E3 SUMO-protein ligase PIAS2	O75928	*PIAS2*	1.698	7	68.2
Mothers against decapentaplegic homolog 2	Q15796	*SMAD2*	1.688	4	52.3
Protein HEXIM1	O94992	*HEXIM1*	1.673	7	40.6
Zinc finger protein ZIC 2	O95409	*ZIC2*	1.666	3	55
HIV Tat-specific factor 1	O43719	*HTATSF1*	1.658	27	85.8
TRAF2 and NCK-interacting protein kinase	Q9UKE5	*TNIK*	1.654	16	154.8
GMP synthase [glutamine-hydrolyzing]	P49915	*GMPS*	1.634	16	76.7
Retinoid-inducible serine carboxypeptidase	Q9HB40	*SCPEP1*	1.624	8	50.8
Methyl-CpG-binding domain protein 2	Q9UBB5	*MBD2*	1.618	9	43.2
ATP-dependent DNA/RNA helicase DHX36	Q9H2U1	*DHX36*	1.617	38	114.7
Zinc finger CCCH-type antiviral protein 1-like	Q96H79	*ZC3HAV1L*	1.602	7	32.9
Far upstream element-binding protein 2	Q92945	*KHSRP*	0.624	8	73.1
Vacuolar protein sorting-associated protein 4B	O75351	*VPS4B*	0.624	13	49.3
Inactive phospholipase C-like protein 2	Q9UPR0	*PLCL2*	0.623	10	125.8
Elongation factor 2	P13639	*EEF2*	0.621	73	95.3
Integrin-linked protein kinase	Q13418	*ILK*	0.619	15	51.4
Eukaryotic translation initiation factor 3 subunit M	Q7L2H7	*EIF3M*	0.614	19	42.5
Cdc42-interacting protein 4	Q15642	*TRIP10*	0.61	13	68.3
Eukaryotic translation initiation factor 3 subunit K	Q9UBQ5	*EIF3K*	0.603	6	25
BAG family molecular chaperone regulator 3	O95817	*BAG3*	0.601	21	61.6
Ribonucleoside-diphosphate reductase large subunit	P23921	*RRM1*	0.592	18	90
Epiplakin	P58107	*EPPK1*	0.589	9	555.3
Phostensin	Q6NYC8	*PPP1R18*	0.581	8	67.9
NAD-dependent protein deacetylase sirtuin-6	Q8N6T7	*SIRT6*	0.579	7	39.1
mRNA cap guanine-N7 methyltransferase	O43148	*RNMT*	0.576	19	54.8
Mitochondrial glutamate carrier 1	Q9H936	*SLC25A22*	0.571	9	34.4
Protein kinase C and casein kinase substrate in neurons protein 3	Q9UKS6	*PACSIN3*	0.569	10	48.5
Ribosome biogenesis protein NOP53	Q9NZM5	*NOP53*	0.567	4	54.4
Nuclear pore membrane glycoprotein 210	Q8TEM1	*NUP210*	0.567	14	205
Alpha-parvin	Q9NVD7	*PARVA*	0.564	9	42.2
RNA-binding protein 20	Q5T481	*RBM20*	0.564	10	134.3
Carnitine O-palmitoyltransferase 1, liver isoform	P50416	*CPT1A*	0.558	3	88.3
Threonine synthase-like 1	Q8IYQ7	*THNSL1*	0.557	2	83
Alpha-internexin	Q16352	*INA*	0.555	6	55.4
Sodium bicarbonate cotransporter 3	Q9Y6M7	*SLC4A7*	0.555	11	136
Zinc finger and BTB domain-containing protein 7A	O95365	*ZBTB7A*	0.554	6	61.4
Beta-parvin	Q9HBI1	*PARVB*	0.549	8	41.7
Ubiquitin-like modifier-activating enzyme ATG7	O95352	*ATG7*	0.546	14	77.9
Ribonuclease inhibitor	P13489	*RNH1*	0.543	5	49.9
60S acidic ribosomal protein P1	P05386	*RPLP1*	0.538	2	11.5
Mitochondrial mRNA pseudouridine synthase RPUSD3	Q6P087	*RPUSD3*	0.524	3	38.4
E3 ubiquitin-protein ligase RAD18	Q9NS91	*RAD18*	0.523	11	56.2
Kinesin-like protein KIF18A	Q8NI77	*KIF18A*	0.517	3	102.2
Krev interaction trapped protein 1	O00522	*KRIT1*	0.515	3	84.3
Glutamine and serine-rich protein 1	Q2KHR3	*QSER1*	0.513	7	189.9
Endonuclease/exonuclease/phosphatase family domain-containing protein 1	Q7L9B9	*EEPD1*	0.509	7	62.4
Very-long-chain 3-oxoacyl-CoA reductase	Q53GQ0	*HSD17B12*	0.509	7	34.3
Solute carrier family 35 member E1	Q96K37	*SLC35E1*	0.508	2	44.7
Histone-lysine N-methyltransferase SETMAR	Q53H47	*SETMAR*	0.505	4	78
Phosphatidylinositol 4-phosphate 3-kinase C2 domain-containing subunit alpha	O00443	*PIK3C2A*	0.499	27	190.6
Shootin-1	A0MZ66	*SHTN1*	0.49	12	71.6
LIM and senescent cell antigen-like-containing domain protein 1	P48059	*LIMS1*	0.482	2	37.2
Electrogenic sodium bicarbonate cotransporter 1	Q9Y6R1	*SLC4A4*	0.481	3	121.4
Cytosolic non-specific dipeptidase	Q96KP4	*CNDP2*	0.47	10	52.8
Microtubule-associated protein 1B	P46821	*MAP1B*	0.47	46	270.5
FSD1-like protein	Q9BXM9	*FSD1L*	0.457	3	59.5
Neurabin-1	Q9ULJ8	*PPP1R9A*	0.452	4	123.3
Inositol 1,4,5-trisphosphate receptor type 3	Q14573	*ITPR3*	0.448	7	303.9
Nuclear factor of activated T-cells, cytoplasmic 1	O95644	*NFATC1*	0.446	5	101.2
Carbonic anhydrase-related protein	P35219	*CA8*	0.44	4	33
Phosphatidate cytidylyltransferase, mitochondrial	Q96BW9	*TAMM41*	0.432	2	51
TLE family member 5	Q08117	*TLE5*	0.431	1	22
Mitochondrial import inner membrane translocase subunit Tim21	Q9BVV7	*TIMM21*	0.43	3	28.2
Neutral amino acid transporter B(0)	Q15758	*SLC1A5*	0.429	4	56.6
Excitatory amino acid transporter 1	P43003	*SLC1A3*	0.427	2	59.5
Metalloreductase STEAP3	Q658P3	*STEAP3*	0.408	3	54.6
DNA-binding protein SATB1	Q01826	*SATB1*	0.329	4	85.9
EKC/KEOPS complex subunit LAGE3	Q14657	*LAGE3*	0.01	2	14.8
Fibrosin-1-like protein	Q9HCM7	*FBRSL1*	0.01	2	110.8
Polypyrimidine tract-binding protein 3	O95758	*PTBP3*	0.01	2	59.7
tRNA-dihydrouridine(20) synthase [NAD(P)+]-like	Q9NX74	*DUS2*	0.01	2	55

### Phosphoproteins interactions analysis

**Fig. 4:**
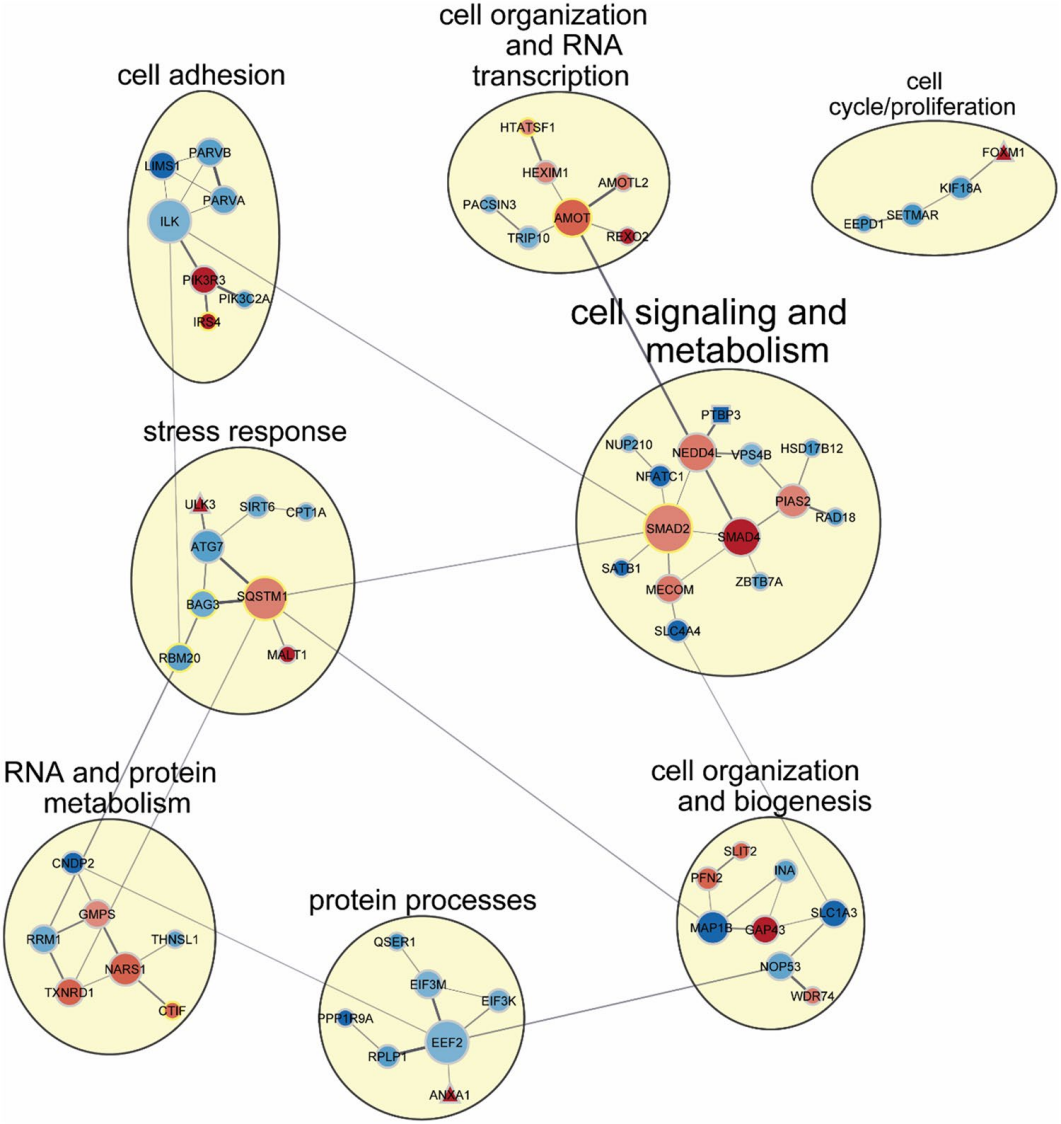
Protein interactions and biological processes shared by phosphoproteins significantly dysregulated in FLAG-TTBK2-L209F cells. Nodes gradient colors represent the relative expression according to log2 (L209/WT ratio) values. Upregulated phosphoproteins are in reddish colors and the ones recovered only in FLAG-TTBK2-L209F cells (“exclusive”) have triangularly shaped nodes. Downregulated phosphoproteins are in bluish colors and phosphoproteins not recovered in FLAG-TTBK2-L209F cells (“lost”) are shown as rectangular shaped nodes. The node size and edge width are according to the node degree of distribution and STRING score retrieved in the network analysis. Phosphoproteins with identified phosphorylation sites in our data are highlighted with a yellow margin. The protein-protein interaction map was performed using stringAPP from Cytoscape 3.9.1. Network clustering (GLay) and annotations based on GO: Biological Process terms were performed with the AutoAnnotate plugin version 1.3.5.

To explore potential interactions and functional consequences of DEPs, a PPI network was generated (Fig. 4) along with associated Gene Ontology (GO) Biological Process terms. In this network, eight major clusters were identified (Fig. 4), with central nodes corresponding to either upregulated phosphoproteins – SMAD2, p62, AMOT, and NARS1 or downregulated phosphoproteins – EEF2, ILK, MAP1B, and KIF18A, in FLAG-TTBK2-L209F cells. Some phosphoproteins were found exclusively in FLAG-TTBK2-L209F cells (“exclusive”; ANXA1, FOXM1 and ULK3), while others were identified only in FLAG-TTBK2-WT cells (“lost”; PTBP3) (Fig. 4; triangular and rectangular shaped nodes, respectively).

The largest cluster, linked to cell signaling and metabolism, centered on the upregulated phosphoproteins SMAD2, SMAD4 and NEDD4L, all involved in the TGF-β signaling pathway^43^. Conversely, downregulated factors at the periphery of this cluster, namely SATB1, ZBTB7A, and PTBP3, may regulate transcription through the TGF-β signaling pathway^44–46^. The p62 cluster highlighted the stress response process, presenting several autophagy-related proteins, with BAG3 and ATG7 downregulated, while ULK3 and p62 itself were upregulated. The MAP1B cluster, related to cell organization and biogenesis, included NOP53 and WDR74, both involved in ribosomal biogenesis and RNA metabolism^47^. The ILK cluster associated with cell adhesion comprised proteins of the PINCH-ILK-Parvin (PIP) complex, including ILK, LIMS1, PARVA, and PARVB (all downregulated), which provide critical links between integrins and the actin cytoskeleton^48^. In the EEF2 cluster associated with protein processes, most phosphoproteins were downregulated and involved in translation. The AMOT cluster was engaged in cell organization and RNA transcription and contained mostly upregulated phosphoproteins. In addition, downregulated TRIP10 and PACSIN3 play a role in actin cytoskeleton reorganization during vesicle formation and endocytosis^49,50^. The NARS1 cluster was associated with RNA and protein metabolism, while the small KIF18A cluster was associated with cell cycle and cell proliferation (Fig. 4).

**Table 2:** List of phosphorylated sites identified in differentially expressed proteins (DEPs). The phosphorylated residues are represented in red lowercase letters. The phosphorylation sites were annotated using PhosphositePlus v6.7.1.1, Phospho.ELM v.9.0, and PhosphoNet databases.

**Protein**	**Accession No.**	**Phospho-sites**	**ptmRS proba-** **bility**	**Phosphorylation peptide sequence**	**State**	**Upstream kinases** **(in vitro**)	**Downstream effects on modified protein**	**Downstream effects on biological processes**	**Downstream effects on interactions**
**Upregulated**									
p62/ SQSTM1	Q13501	Ser272	100	LTPVsPESSSTEEK	Confirmed in mammals	CDK1; MAPK13 (P38D)	Intracellular localization; Molecular association - regulation; protein degradation; ubiquitination	Autophagy - inhibited; Carcionogenesis - altered/induced; Cell cycle regulation; Signaling pathway regulation	Induce interaction - Raptor, TRAF6, mTOR; Inhibit interaction - LC3B
SMAD2	Q15796	Thr8	100	mSSILPFtPPVVK	Confirmed in mammals	MAPK3 (ERK1)	Activity - induced; Molecular association - regulation; protein stabilization	Transcription - induced	Induce interaction - SMAD4
AMOT	Q4VCS5	Ser200	100	AHPPVTSAPLsPPQPNDLYK	Predicted	-	-	-	-
		Ser309	99.2	NSQPHsPTSSLTSGGSLPLLQSPPSTR	Confirmed in mammals	-	-	-	-
		Ser332	100	NSQPHSPtSSLTSGGSLPLLQSPPSTRLsPAR	Predicted	-	-	-	-
		Ser714	100	DTTVISHsPNTSYDTALEAR	Confirmed in mammals	-	-	-	-
		Ser808	100	SLMSISnAGSGLLSHSSTLTGsPIMEEK	Predicted	-	-	-	-
		Thr1061	100	TDGPVFHSNtLER	Confirmed in mammals	-	-	-	-
CTIF	O43310	Ser299	100	LEDTAGDTGHSSLEAPRsPDTLAPVASER	Confirmed in mammals	-	-	-	-
NEDD4L	Q96PU5	Ser448	100	SLsSPTVTLSAPLEGAK	Confirmed in mammals	PKACA; SGK1	Activity - induced; Molecular association - regulation; phosphorylation; protein conformation and stabilization; ubiquitination	Signaling pathway regulation	-
HTATSF1	O43719	Ser498	100	ESEEGNPVRGSEEDsPKK	Confirmed in mammals	-	-	-	-
		Ser642	100	VFDDEsDEKEDEEYADEK	Confirmed in mammals	-	-	-	-
		Ser676	100	LFEEsDDKEDEDADGKEVEDADEK	Confirmed in mammals	-	-	-	-
TNIK	Q9UKE5	Ser640	100	QNsDPTSENPPLPTR	Confirmed in mammals	-	-	-	-
IRS4	O14654	Ser757	100	VsPPPAPsPPKAPDT	Predicted	-	-	-	-
**Downregulated**									
BAG3	O95817	Ser173	100	SQsPAASDCSSSSSSASLPSSGR	Confirmed in mammals	-	-	-	-
RBM20	Q5T481	Ser742	99.6	SGsPNLPHSVSSYK	Confirmed in mammals	-	-	-	-
		Ser1048	99.6	GVESSDVHPAPTVQQMSsPKPAEER	Predicted	-	-	-	-

Predicted - Predicted by Kinexus P-Site Prediction algorithm.

### Functional enrichment analysis

 We used the web tool WebGestalt to conduct over-representation analysis (ORA) in our set of phosphoproteins (Table S6). We found seven statistically significant upregulated pathways in FLAG-TTBK2-L209F cells: embryonic development, negative transcriptional regulation, negative regulation of organelle organization, and cellular responses to stress. Interestingly, SMAD4 and SMAD2 were present in more than half of these enriched sets, while SLIT2, PIK3R3, and histone proteins (H3-3A/B and H3C13/14/15) were identified in more than one enriched set. Furthermore, we identified one downregulated pathway “Cell-extracellular matrix interactions” that includes the ILK and parvin proteins (Table S6).

 Additionally, gene set enrichment analysis (GSEA) was performed to obtain more insights into the biological alterations caused directly or indirectly by L209F (Table S7). In FLAG-TTBK2-L209F cells, among the enriched pathways [with positive normalized enriched score (NES)], we highlight the sets related to cytoskeleton regulation, such as “Ciliary part”, “Regulation of Actin Cytoskeleton” and “Regulation of Cytoskeleton Organization”. In addition, several enriched pathways included several members of the histone clusters (e.g., WP2369). Furthermore, the FLAG-TTBK2-L209F cells were found to lack phosphoproteins involved in translation and transcription pathways. Among these negatively enriched pathways, there were several ribosomal and mitochondrial ribosomal proteins, as well as several members of the eukaryotic initiation factor 3 (eIF3) multiprotein complex. In this data, we also observed negative NES in sets related to gene expression and mitochondrial activity. Interestingly, a decrease in Parkinson Disease (PD)-associated phosphoproteins, such as PARK7 and tau, was observed (Table S7).

### Potential TTBK2 targets in transcription and autophagy

Among the proteins potentially affected by the L209F variant in the MS/MS data, many were associated with transcription and autophagy/protein degradation pathways. This prompted us to investigate several proteins involved in these biological processes in more detail. Unfortunately, due to the lack of available antibodies, we were unable to consistently confirm the phosphorylation state or specific phosphorylation sites of all targets.

**Fig. 5:**
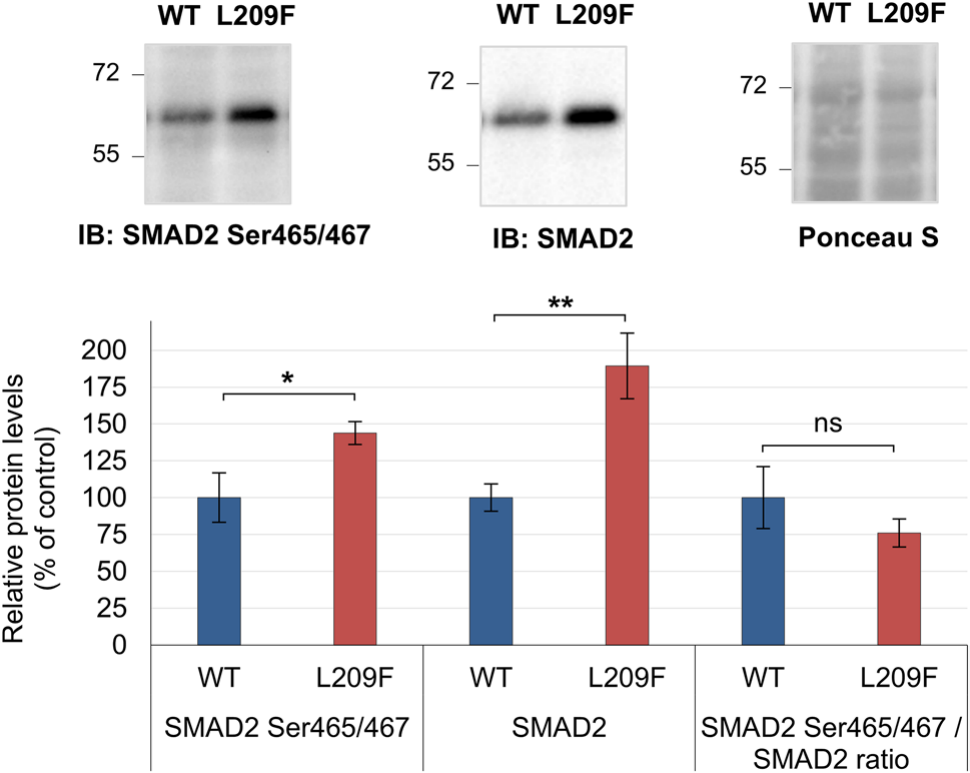
SMAD2 protein expression was elevated in FLAG-TTBK2-L209F cells. Analysis of SMAD2 Ser465/467 and total SMAD2 protein levels in FLAG-TTBK2-WT (WT) and FLAG-TTBK2-L209F (L209F) by immunoblotting using specific antibodies. SMAD2 Ser465/467 and total SMAD2 levels were normalized to Ponceau S staining (loading control). SMAD2 Ser465/467 phosphorylation levels normalized to total SMAD2 levels are also shown. Quantification data are presented in percentage as mean ± SD of three independent experiments; non-significant (ns), *P ≤ 0.05 and **P ≤ 0.01 compared with the WT cell line (t-test).

We started by analyzing the transcription factor SMAD2 (upregulated phosphoprotein) and the state of SMAD2 phosphorylation at Ser465/467, which is crucial for SMAD2-mediated TGF-β signaling^51^. We confirmed that the levels of SMAD2 phosphorylated at Ser465/467 were significantly increased by 43% (*p* = 0.015), accompanied by a simultaneous increase in SMAD2 protein expression (89%, *p* = 0.003) in FLAG-TTBK2-L209F cells (Fig. 5). But there were no significant differences between cell lines when the phosphorylated levels were normalized to total protein levels. Nevertheless, SMAD2 high levels suggest transcriptional dysregulation in FLAG-TTBK2-L209F cells.

**Fig. 6:**
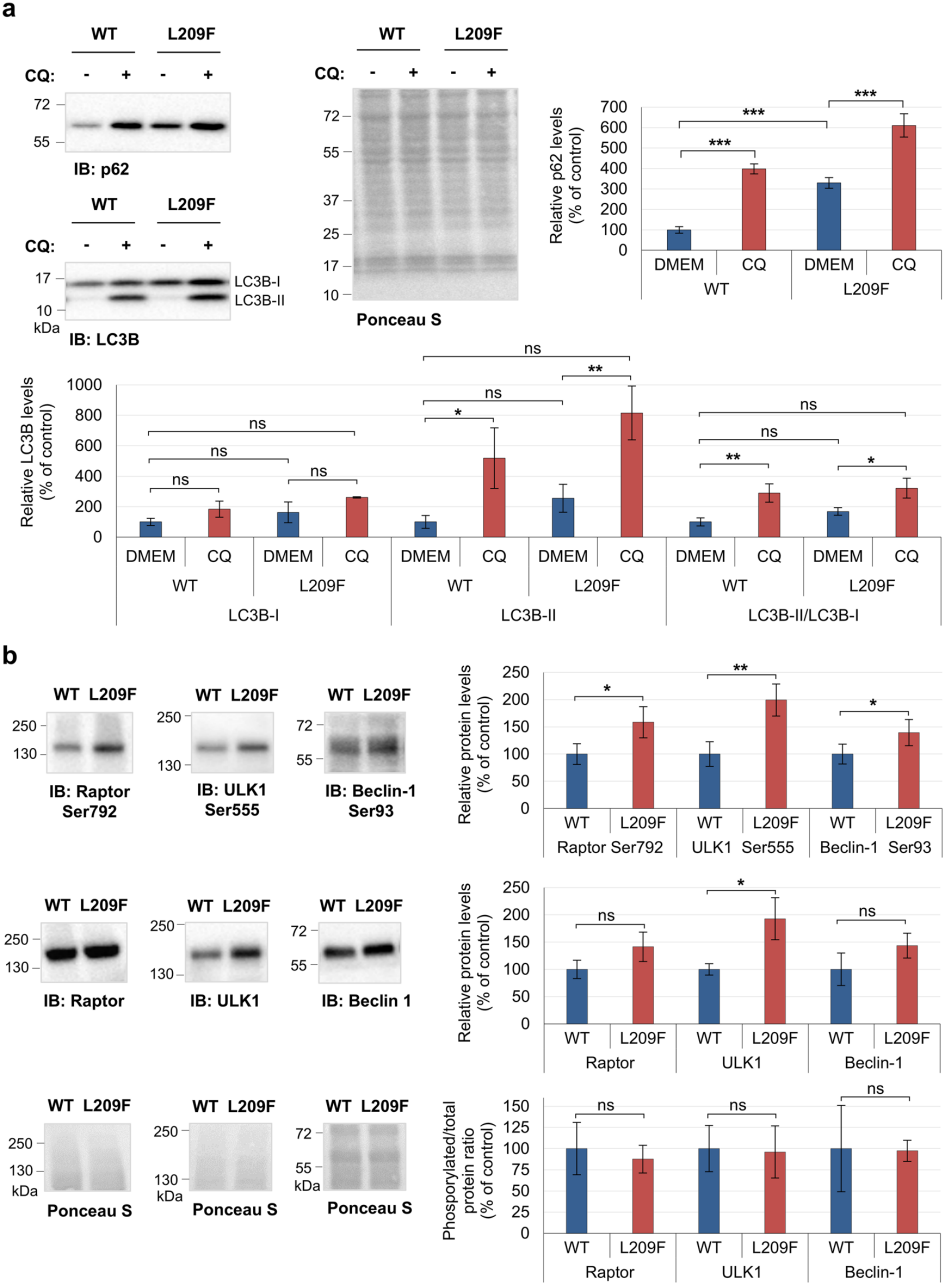
The expression of several autophagy-related proteins is increased in FLAG-TTBK2-L209F cells but the autophagic flux seems unaffected. **(a)** Protein expression analysis of p62, LC3B-I, LC3B-II and LC3B-II/LC3B-I ratio in non-treated FLAG-TTBK2-WT (WT) and FLAG-TTBK2-L209F (L209F) cells (incubated with the control vehicle DMEM) and in cells incubated with 10 µM chloroquine (CQ) for autophagy blockage. Immunoblotting was performed using specific antibodies and Ponceau S staining was used as loading control. **(b)** Protein expression analysis of the upstream autophagy-related phosphoproteins raptor, ULK1 and beclin-1 in WT and L209F cells by immunoblotting. Raptor Ser792, ULK1 Ser555 and Beclin-1 Ser93 phosphorylated levels, as well as total raptor, ULK1 and beclin-1 levels were normalized to Ponceau S staining (loading control). Phosphorylated levels of raptor, ULK1 and beclin-1 were also normalized to the respective total protein levels. Quantification data are presented in percentage as mean ± SD of at least three independent experiments; non-significant (ns), *P ≤ 0.05, **P ≤ 0.01 and ***P ≤ 0.001 compared with the WT cell line and with non-treated cells in panel (a) (one-way ANOVA/Tukey); non-significant (ns), *P ≤ 0.05 and **P ≤ 0.01 compared with the WT cell line in panel (b) (t-test).

Another phosphoprotein – p62 – was also upregulated in FLAG-TTBK2-L209F cells (about a threefold increase compared to non-treated WT cells, *p* ≤ 0.001); Fig. 6a). Since p62 accumulation has been linked to impaired protein degradation by the autophagic system^52^, we analyzed the levels of other autophagy-related proteins and phosphorylated forms that are critical for autophagy induction. Phosphorylated levels of raptor (Ser792), ULK1 (Ser555), and beclin-1 (Ser93) were significantly increased (approximately 60, 100 and 40%; *p* = 0.042, 0.01 and 0.041, respectively) in FLAG-TTBK2-L209F cells, accompanied by an increase in the total expression levels of the respective proteins (Fig. 6b), although only ULK1 increase reached statistical significance (increase of 92%, *p* = 0.016). However, no significant differences were observed between cell lines when the phosphorylated levels were normalized to total protein levels.

To investigate further if TTBK2-L209F cells had an altered autophagic flux, we treated cells with chloroquine, an inhibitor of autophagic flux by decreasing autophagosome-lysosome fusion^52^. We did not find statistically significant differences in either LC3B-I, LC3B-II or LC3B-II/LC3B-I ratio between non-treated FLAG-TTBK2-WT and L209F cells (Fig. 6a). Nevertheless, the levels of LC3B-II increased approximately five and eight times in FLAG-TTBK2-WT and L209F cells, respectively, following drug treatment as expected (*p* = 0.029 and *p* = 0.006; Fig. 6a). In accordance, the LC3B-II/LC3B-I ratio increased approximately three times in both cell lines treated with chloroquine (*p* = 0.006 and 0.019; Fig. 6a). Similarly, p62 levels increased approximately four and two times after drug treatment in FLAG-TTBK2-WT and -L209F cells, respectively (*p* ≤ 0.001; Fig. 6a). Together these results suggested that autophagic flux was not impaired in the presence of the L209F variant.

Furthermore, we measured the levels of the mRNAs encoding these proteins to ascertain whether elevated protein levels were caused by transcriptional activation rather than dysfunctional protein degradation. The mRNA levels of SMAD2, p62, LC3B and beclin-1 were significantly increased (3.4, 0.5, 0.8 and 1.7 fold change; *p* = 0.012, 0.006, 0.05 and 0.021 respectively) while raptor and ULK1 mRNAs levels tended to decrease in FLAG-TTBK2-L209F cells (Fig. S6), suggesting that expression changes may be, at least in part, due to transcription regulation for most of these proteins.

Additionally, since proteasome inhibition can induce p62 synthesis^52^, we assessed the protease activities associated with the proteasome complex. Chymotrypsin, trypsin, and caspase-like protease activities were slightly increased in FLAG-TTBK2-L209F cells compared to WT cells, but the differences were not statistically significant (Fig. S7), indicating normal proteasome function.

## Discussion

In this study, we demonstrated for the first time the detrimental impact of a missense variant in TTBK2 (c.625 C > T, p.Leu209Phe; Fig. 1). While all confirmed SCA11 cases to date have been linked to truncating variants in *TTBK2*, our findings highlight the potential for missense variants to disrupt TTBK2 function, particularly those within the kinase domain. The TTBK2-L209F variant described here is extremely rare, with only three heterozygous alleles identified in the European non-Finnish population. Thereby, incomplete disease penetrance or underdiagnosis until later stages of disease progression cannot be ruled out.

To ascertain the potential pathogenicity of TTBK2-L209F, we conducted functional studies using a HEK293T cell model genetically modified by CRISPR/Cas9 to express the *TTBK2* missense variant in homozygosity (Fig. 2a). While this model does not fully replicate the disease context, it provided a reliable system to investigate the variant’s impact on TTBK2 expression and molecular functions.

Our results provided evidence for a loss of function mechanism underlying the pathogenesis of TTBK2-L209F, as shown by reduced TTBK2 expression and kinase activity. The variant caused reduced TTBK2 protein levels while maintaining normal mRNA expression, likely due to decreased protein stability (Fig. 2b and c). In contrast, previous studies of *TTBK2* truncating variants reported a partial reduction in mRNA levels, suggestive of premature degradation by NMD^15^. Consistent with prior findings^8,9,29^, our data showed impaired kinase activity of mutated TTBK2. Specifically, analysis of the phosphorylation state of TDP-43 at Ser409/410, a known target of TTBK2^11,12^, showed that TTBK2-L209F has a reduced capacity to phosphorylate TDP-43 (Fig. 3). Furthermore, these experiments did not support a dominant negative effect, since TTBK2-L209F was not able to interfere with the ability of TTBK2-WT to phosphorylate TDP-43 (Fig. 3b). Although, this mechanism cannot be entirely ruled out, as the levels of both endogenous and overexpressed TTBK2-L209F proteins were lower in comparison with WT proteins, potentially skewing the results.

Cytoskeleton abnormalities were identified in the FLAG-TTBK2-L209F cell line (Fig. 2d), specifically reduced tubulin acetylation and KIF2A levels. Future studies are required to determine if these effects were a direct result of impaired TTBK2 kinase activity against its cytoskeletal targets or were indirectly influenced by other mechanisms. Nevertheless, we hypothesize that reduced TTBK2 expression or kinase activity could lead to microtubule dynamic instability, resulting in reduced tubulin acetylation, as TTBK2 functions as a + TIP^6,7^and phosphorylates tubulin and other microtubule-associated proteins (MAPs)^2,3^. Another plausible scenario is that TTBK2 could indirectly influence tubulin acetylation by phosphorylating TDP-43. TDP-43 is known to influence neurite outgrowth by regulating tubulin deacetylase HDAC6 levels^53^, which were elevated in FLAG-TTBK2-L209F cells (Fig. 2). Deficits in tubulin acetylation can alter the MAP landscape, leading to impairment of axonal transport, abnormal polarization and migration in neurons, all hallmarks of many neurodegenerative diseases^54^. For example, increasing microtubule acetylation has been shown to rescue axonal transport and locomotor deficits in Huntington’s disease (HD) and PD, respectively, and to revert axonal loss in Charcot-Marie-Tooth disease^54^. Regarding KIF2A, it is known that phosphorylation at Ser135 by TTBK2 inhibits KIF2A interaction with microtubules and decreases its microtubule depolymerizing activity^7^. It remains to be determined whether reduced KIF2A levels correlate with decreased phosphorylation of KIF2A at Ser135, as well as whether these changes are regulated by TTBK2 or are a consequence of cytoskeleton instability. Interestingly, pathogenic variants in *KIF2A* have been linked to cortical dysplasia^55^, and *KIF2A* deficiency has disrupted neurogenesis and axonal transport, leading to neurodegeneration in mice^56^. Moreover, pathogenic variants that disrupt the functions of other kinesins (e.g., *KIF1C*, *KIF7*, *KIF26B*) have been also associated with cerebellar ataxia^57^.

Our analysis of protein phosphorylation in FLAG-TTBK2-L209F cells (Table 1) revealed potential alterations in the phosphorylation of other microtubule-associated proteins (e.g., KIF18A and MAP1B, both downregulated). Interestingly, several actin-binding proteins (e.g., PARVA/B/parvin alpha and beta, PPP1R9A/neurabin 1, and PFN2/profilin 2) were also identified in this dataset, as well as intermediate filament-associated proteins (e.g., EPPK1/epiplakin 1 and INA/alpha-internexin), suggesting that other cytoskeletal components can also be disrupted in FLAG-TTBK2-L209F cells.

Phosphoproteome data also drew our attention towards SMAD2, which was present in several enriched sets in the ORA analysis (Table S6) and emerged as a central node in the PPI network (Fig. 4). Notably, SMAD2 was highly phosphorylated at Thr8 in FLAG-TTBK2-L209F cells (Table 2). This residue is phosphorylated by MAPK3, leading to increased SMAD2 protein levels and complex formation with SMAD4, thereby enhancing transcriptional activity^58^. SMAD4 was also identified as an upregulated phosphoprotein in our MS/MS data, along with NEDD4L (Table 1), which targets SMAD proteins for degradation, modulating the TGF-β pathway^43^. NEDD4L was phosphorylated at Ser488 in our data (Table 2). Dephosphorylation of this residue has been reported to inhibit the activity of NEDD4L E3 ubiquitin ligase by reducing NEDD4L protein levels and regulating neuronal excitability^59^. Therefore, we hypothesize that increased phosphorylation at Ser488 may correlate with elevated NEDD4L protein levels. Additionally, we analyzed the phosphorylation state of SMAD2 at Ser465/467, which was elevated in FLAG-TTBK2-L209F cells, along with total SMAD2 levels (Fig. 5). Phosphorylation of Ser465/467 enables SMAD2 association with SMAD4 and facilitates interaction with other co-factors to regulate gene expression and mediate TGF-β signaling^43,51^. Therefore, TGF-β signaling may be affected in FLAG-TTBK2-L209F cells. Curiously, downregulation of several genes involved in TGF-β signaling, including *daf-14* (the orthologue of SMAD2), partially rescued worm incoordination with an *unc-2* (orthologue of *CACNA1A*; genetic cause of SCA6 and episodic ataxia type 2) truncating variant^60^.

Additional upregulated phosphoproteins involved in transcription and gene expression were identified in our MS/MS data, including various histone proteins, PIAS2/protein inhibitor of activated STAT 2, and histone-lysine N-methyltransferase MECOM (Fig. 4). The precise mechanisms by which reduced TTBK2 expression and/or kinase activity led to the upregulation of phosphoproteins associated with transcription and gene expression remain to be fully elucidated. Intriguingly, several phosphoproteins involved in translation and transcription pathways (Table S6) were downregulated in FLAG-TTBK2-L209F cells, including ribosomal proteins, members of the eIF3 multiprotein complex, and EEF2/elongation factor 2. A pathogenic variant in *EEF2* was found to cause SCA26, by impairing translation and increasing susceptibility to proteostatic disruption^61^. In addition to EEF2, two other downregulated phosphoproteins associated with hereditary cerebellar ataxias were identified in FLAG-TTBK2-L209F cells (Table 1): SLC1A3/excitatory amino acid transporter 1, linked with episodic ataxia type 6 through decreased glutamate uptake^62^; and ATG7, a ubiquitin-activating enzyme E1, implicated in a recessive form of cerebellar ataxia and impaired autophagic flux^63^.

Beyond ATG7, other phosphoproteins involved in protein degradation pathways were significantly dysregulated in FLAG-TTBK2-L209F cells. Among them, p62 may be particularly relevant, since autophagy and proteasome degradation are often impaired in SCAs^64^ and dysregulation of p62 levels has been observed in several neurodegenerative diseases^65^. Phosphorylation of p62 at Ser272 was elevated in FLAG-TTBK2-L209F cells (Table 2). This residue was previously reported to be phosphorylated upon nocodazole treatment during mitosis by CDK1^34^. Also, inflammation-related protein mitogen-activated protein kinase kinase kinase 7 (MAP3K7) phosphorylates p62 at different residues, potentially including Ser272, inhibiting the autophagic degradation of p62 and promoting p62-mediated cellular signaling^67^. Total p62 protein levels were increased in FLAG-TTBK2-L209F cells (Fig. 6a), suggesting autophagy impairment^52^. However, protein levels of several autophagy-associated proteins were also increased, along with its phosphorylated forms raptor (Ser792), beclin-1 (Ser93) and ULK1 (Ser555) (Fig. 6) related to autophagy induction^52,68,69^. Treatment with chloroquine, an autophagic inhibitor that blocks autophagosome-lysosome fusion^52^, resulted in increased LC3B-II and p62 levels, along with an elevated LC3B-II/LC3-I ratio in both cell lines (Fig. 6a), indicating a normal LC3B-II turnover, autophagosome formation, and autophagic flux^52^. Furthermore, mRNA levels of p62 and beclin-1 were also upregulated in FLAG-TTBK2-L209F cells (Fig. S6), indicating that expression changes may be due to transcriptional activation. However, this was not the case for all autophagy proteins, as mRNA levels of ULK1 and raptor tended to decrease in FLAG-TTBK2-L209F cells. It is known that increased protein levels, at least for ULK1, do not always correlate with its mRNA levels under certain conditions^70^. While this study did not confirm whether TGF-β signaling drives these expression changes, previous research has shown that TGF-β can upregulate the mRNA levels of some autophagy-related proteins and activate autophagy in specific cancer cells^71^.

Although our study offers valuable insights, some limitations must be acknowledged. First, our study relies on a single WT and a single clonal mutant cell line, so we cannot fully exclude the possibility that some of the observed differences may reflect clonal variability. Future work will be needed to strengthen these findings, for example by analyzing additional independent clones, performing rescue experiments, or even using complementary model systems. Secondly, our approach to assess TTBK2 kinase activity may provide only an indirect assessment. Thus, a kinase assay directly measuring TTBK2 activity would complement our results on TDP-43 phosphorylation. Finally, our data did not definitively establish the role of deregulated phosphoproteins but highlight several critical aspects that should be explored in future research to better understand TTBK2 biological roles in both health and disease. Additionally, it would be valuable to explore in detail the TTBK2 interactome, which remains superficially characterized, and compare those results with our phosphoproteomics data.

This study provided the first evidence linking a *TTBK2* missense variant (TTBK2-L209F) to a loss of function mechanism. This variant led to impaired protein phosphorylation, potentially disrupting key cellular processes and molecular pathways, including cytoskeleton dynamics, gene regulation and TGF-β signaling. Future studies investigating these processes will be crucial for unraveling the pathogenic mechanisms of TTBK2 dysfunction and its role in the development of cerebellar ataxia.

## Supplementary Information

Below is the link to the electronic supplementary material.

Supplementary Material 1

Supplementary Material 2

## Data Availability

The mass spectrometry proteomics data have been deposited to the ProteomeXchange Consortium via the PRIDE partner repository with the dataset identifier PXD056662. Sanger sequencing data have been deposited in NCBI’s Sequence Read Archive (SRA) database with the accession number PRJNA1375520. Additional data supporting the findings of this study are available from the corresponding author upon reasonable request.
