# Supplementary material for: Missense variant in TTBK2 kinase domain causes loss of function and impaired protein phosphorylation
Source: Sci Rep. 2025 Dec 21;16:2501. doi: 10.1038/s41598-025-32288-0 (PMC12820296; doi:10.1038/s41598-025-32288-0)

**Supplementary file – uncropped blots**

Missense variant in *TTBK2* kinase domain causes loss of function and impaired protein phosphorylation

Daniela Felício, Hugo Osório, Conceição Pereira, Ana Filipa Brandão, João Parente Freixo, Inês Carvalho, Ana Paula Sousa, Margarida Castro-Caldas, Jorge Sequeiros, Carolina Lemos, Mariana Santos

Correspondence: mariana.graca@ibmc.up.pt

**Uncropped blot images.** Original blots corresponding to figures in the main paper and supplementary figures are shown. Black boxes indicate the regions/lanes selected from presentation in the paper. Some membranes were cut considering the protein MW before incubating each part with the respective antibody.


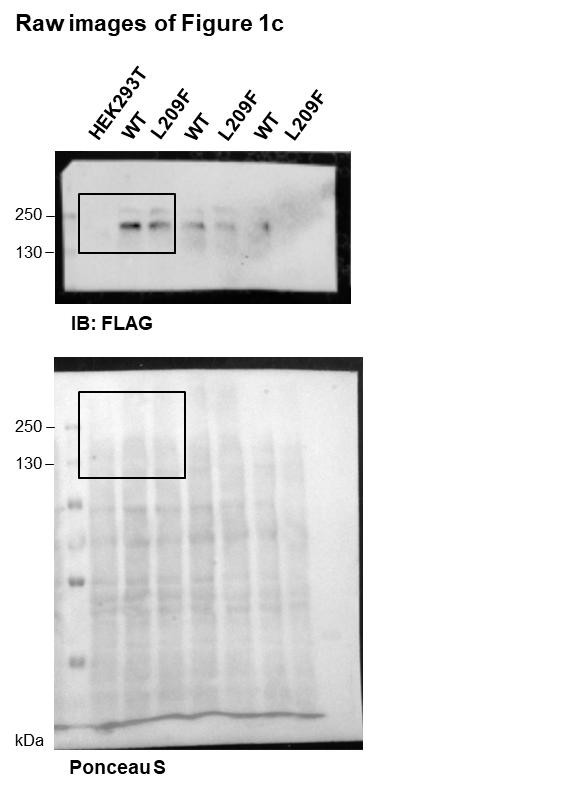


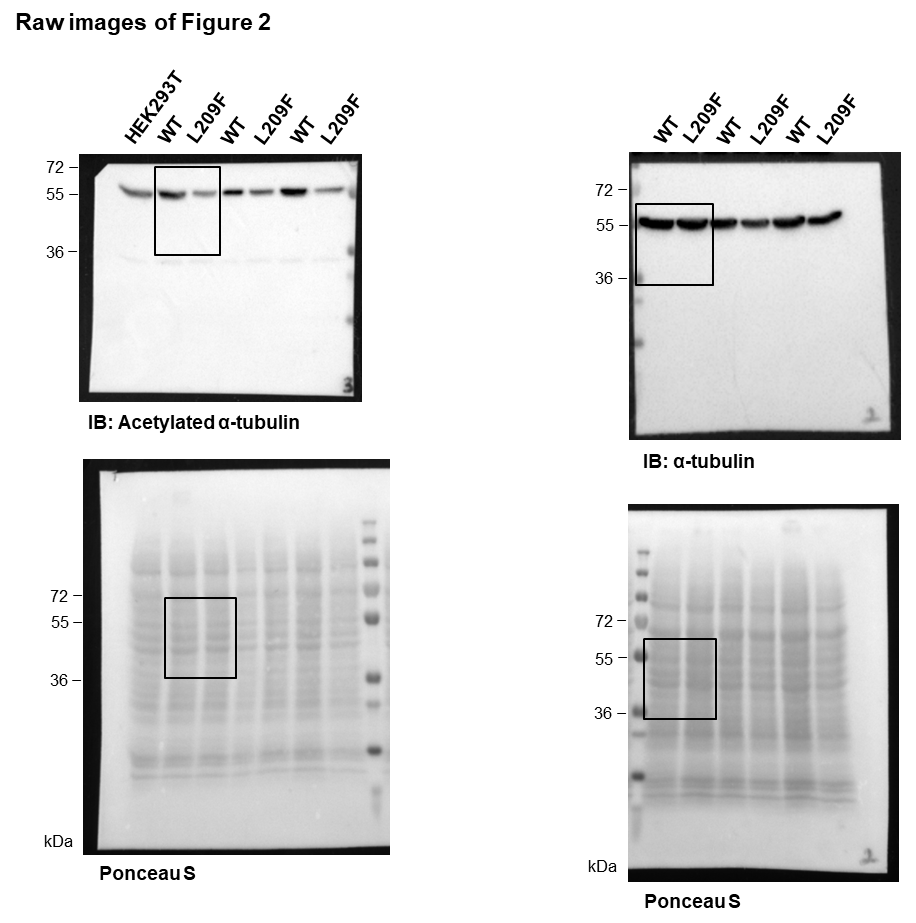


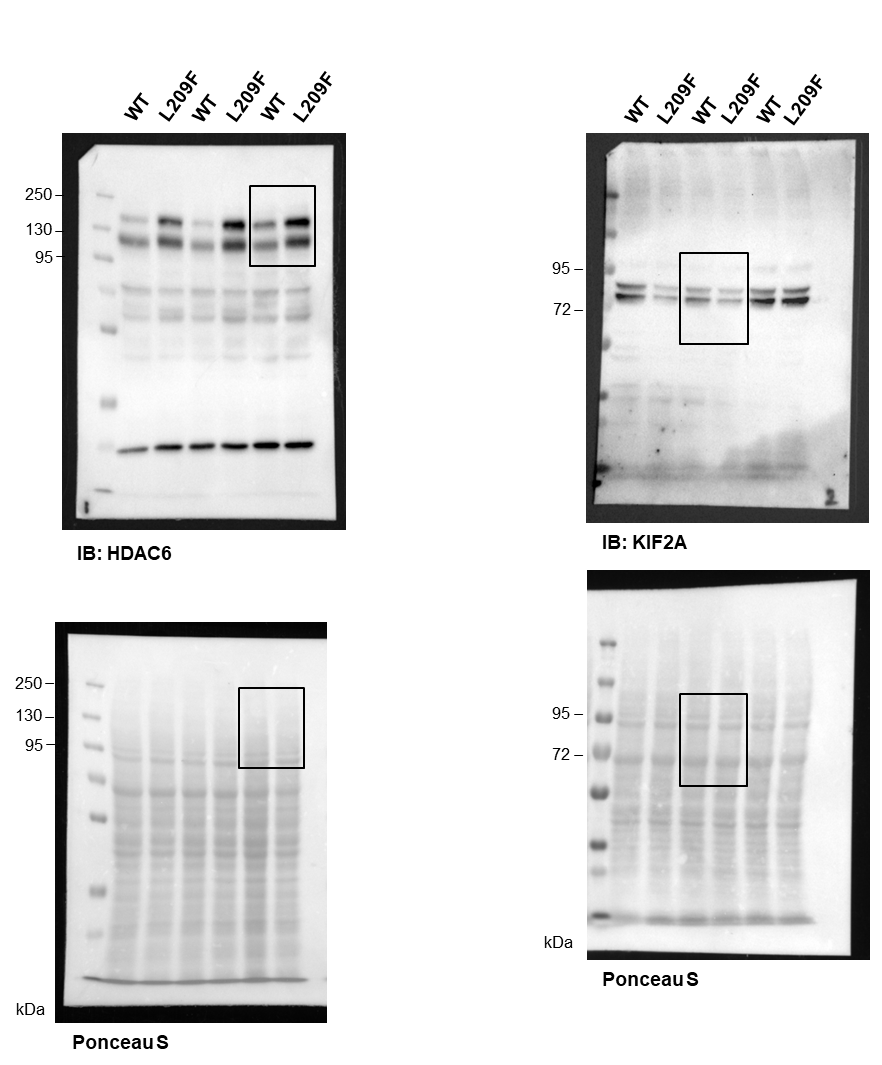


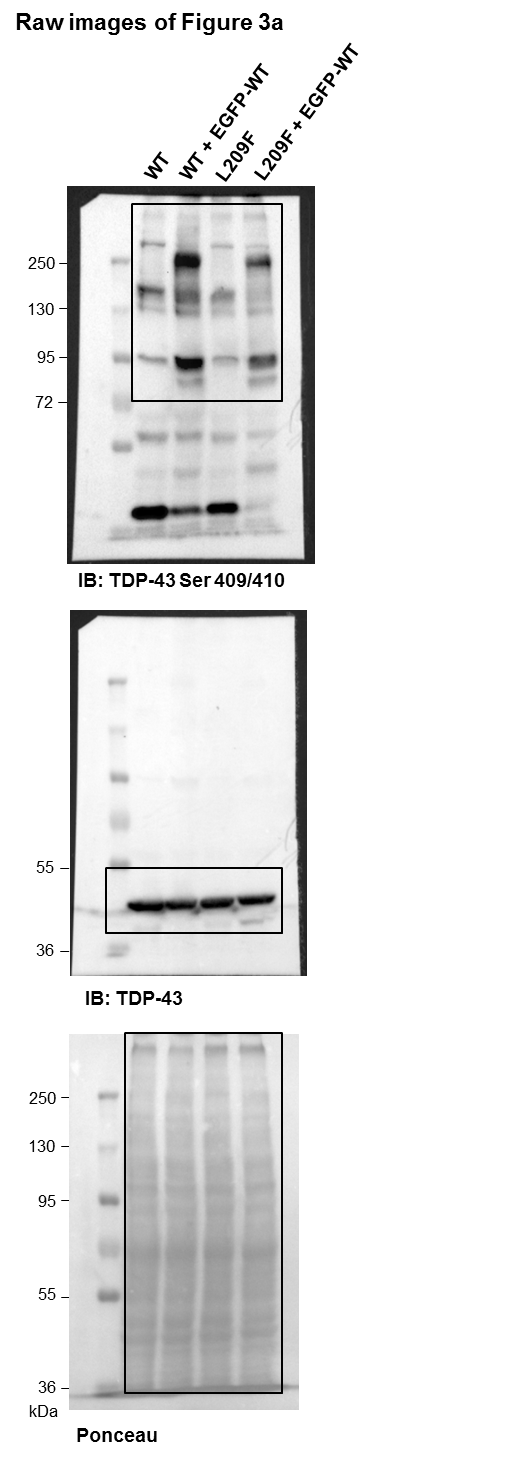


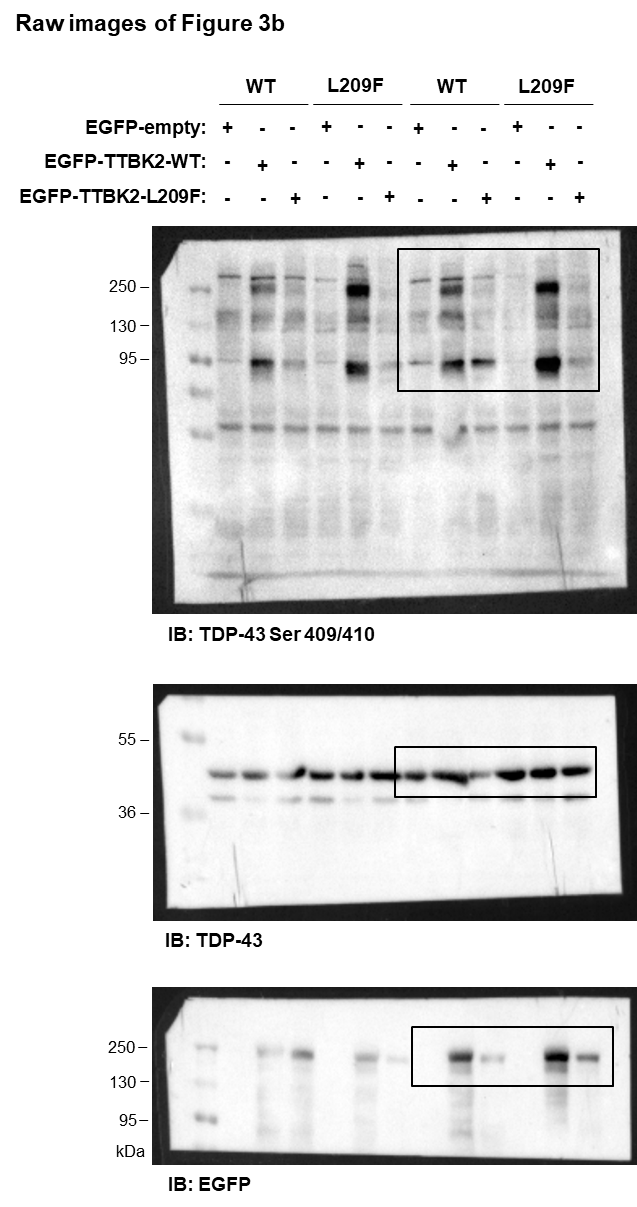


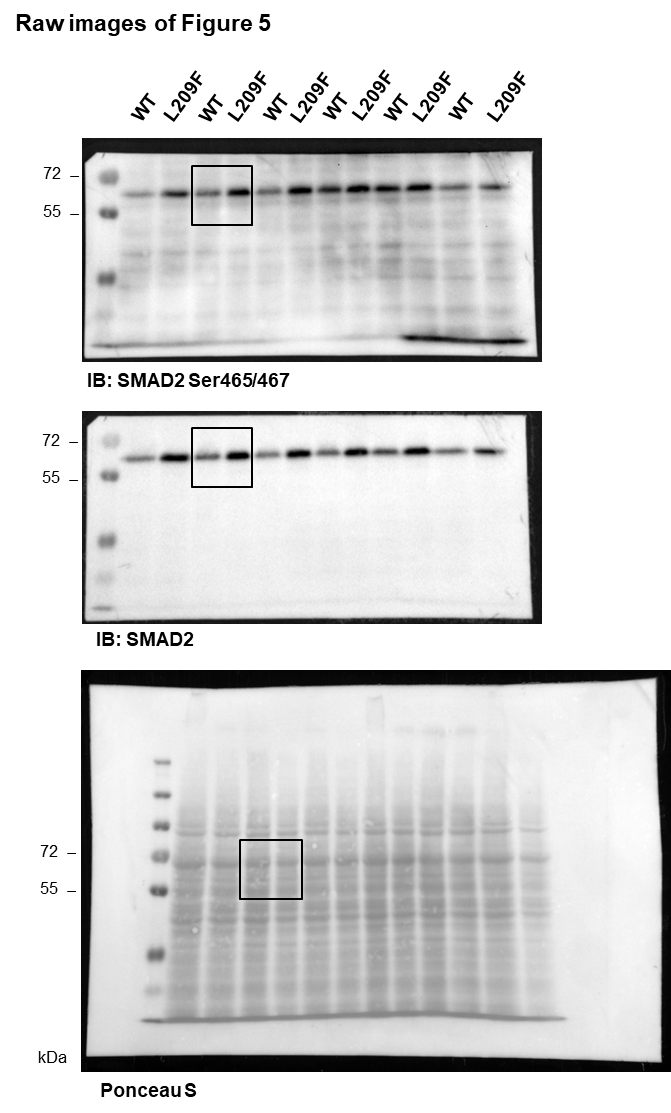


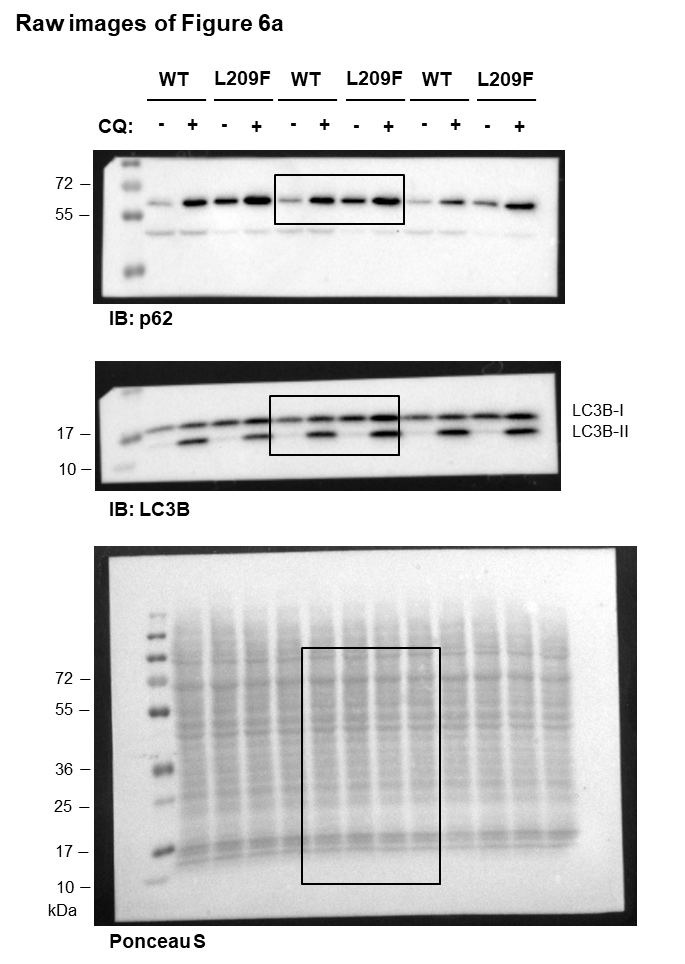


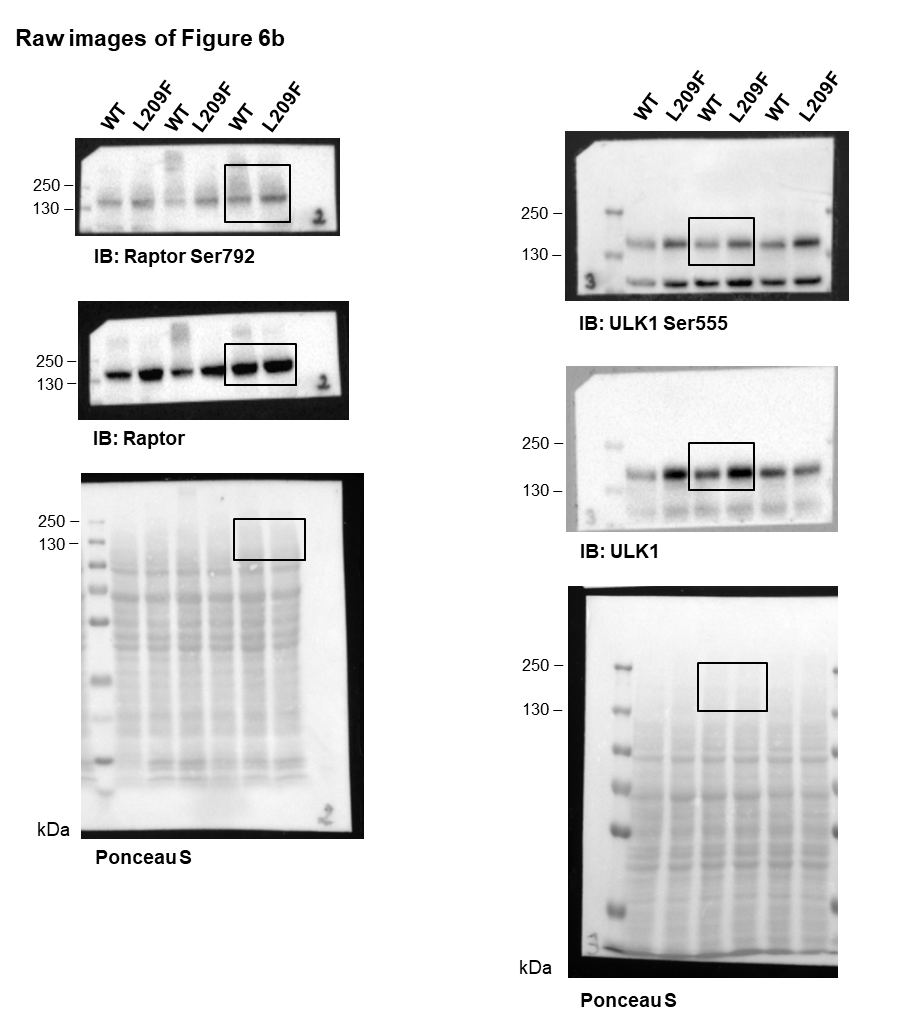


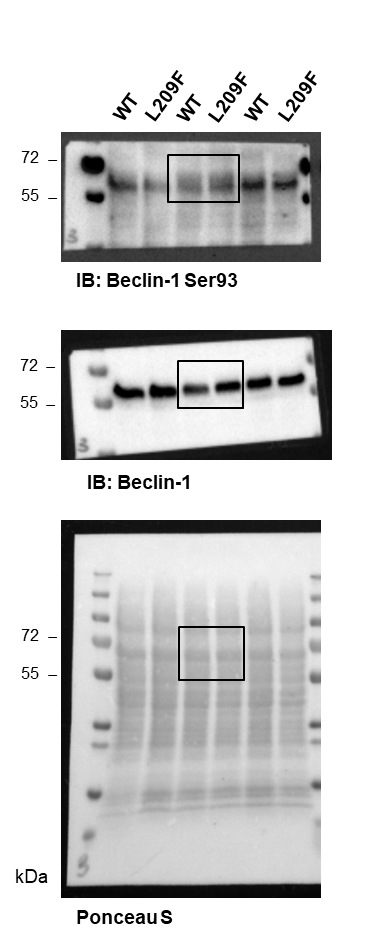


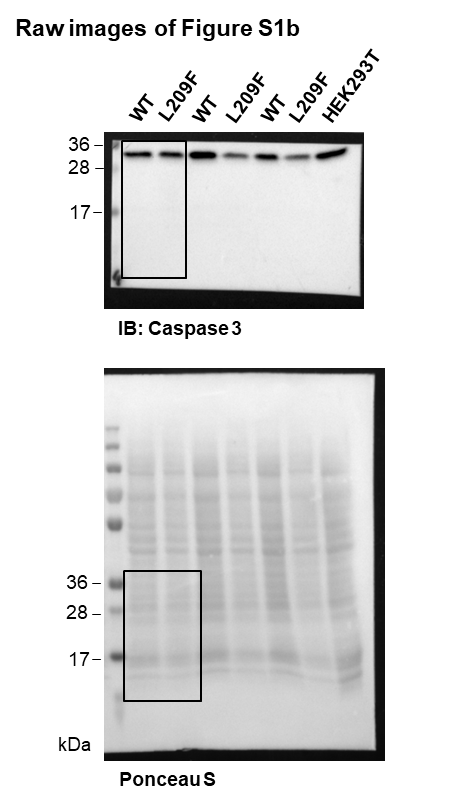


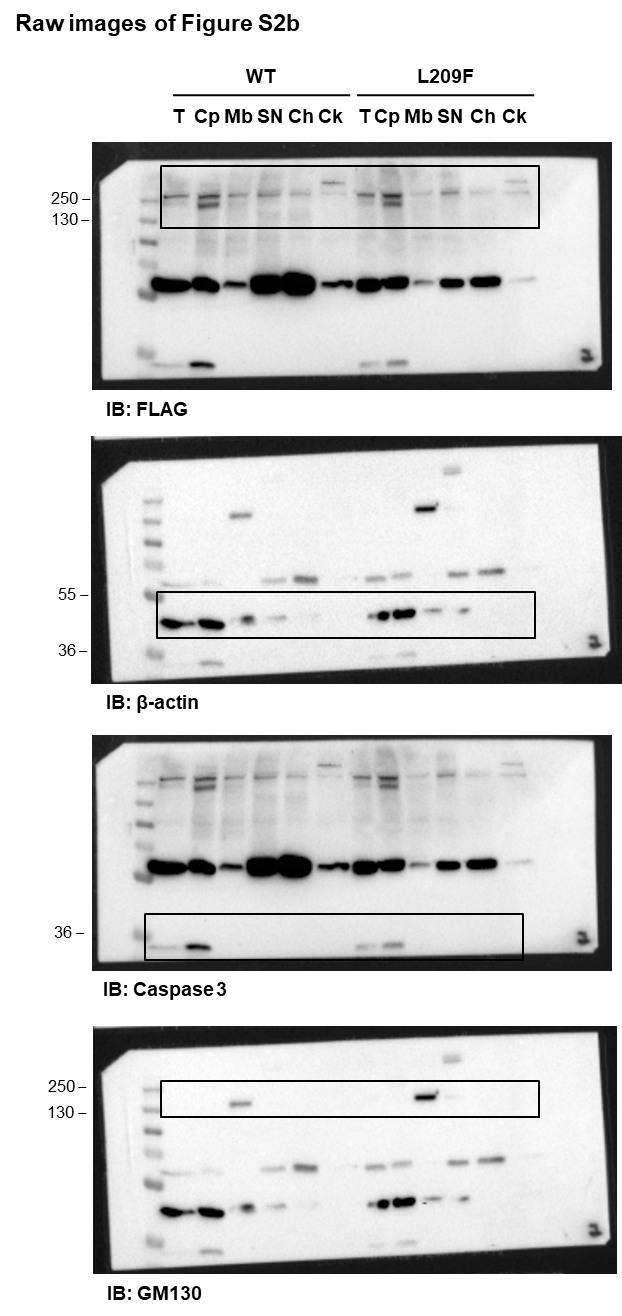


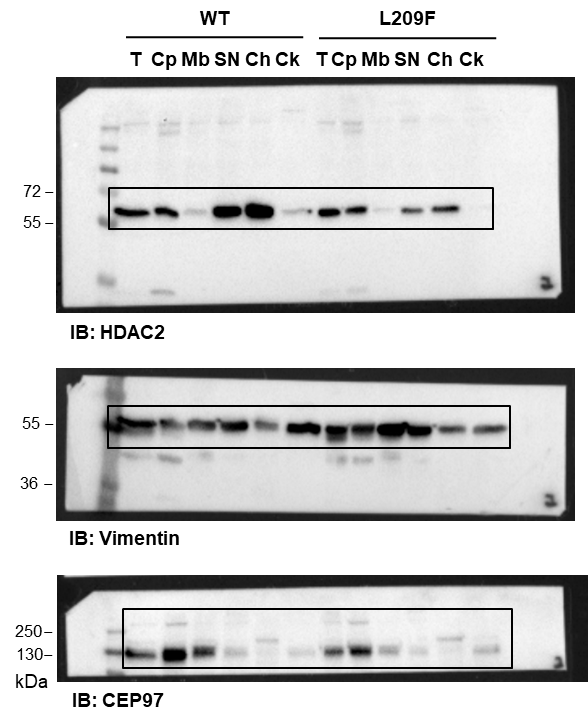


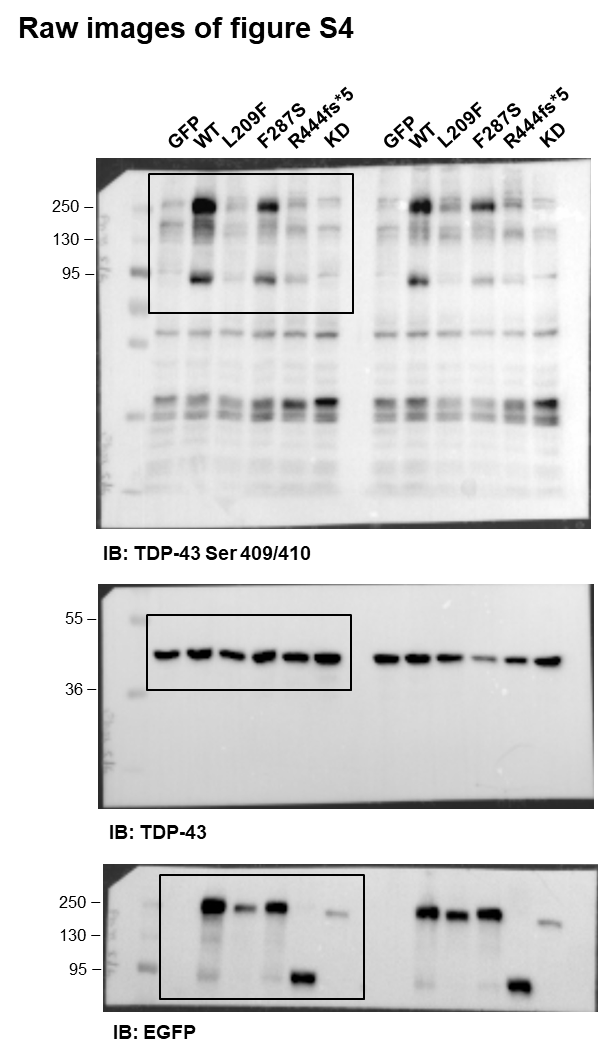

Supplement: Supplementary file 1 — Supplementary Material 1 [file 41598_2025_32288_MOESM1_ESM.docx]
