# Supplementary material for: Missense variant in TTBK2 kinase domain causes loss of function and impaired protein phosphorylation
Source: Sci Rep. 2025 Dec 21;16:2501. doi: 10.1038/s41598-025-32288-0 (PMC12820296; doi:10.1038/s41598-025-32288-0)
Supplement: Supplementary file 2 — Supplementary Material 2 [file 41598_2025_32288_MOESM2_ESM.docx]

**Supplementary Information**

Missense variant in TTBK2 kinase domain causes loss of function and impaired protein phosphorylation

Daniela Felício, Hugo Osório, Conceição Pereira, Ana Filipa Brandão, João Parente Freixo, Inês Carvalho, Ana Paula Sousa, Margarida Castro-Caldas, Jorge Sequeiros, Carolina Lemos, Mariana Santos

Correspondence: mariana.graca@ibmc.up.pt

**Supplementary material and methods**

**Genetic analysis and clinical information**

Clinical and family history collection, along with neurological evaluations, of a patient with cerebellar ataxia were conducted at a Hospital Center in Lisbon, Portugal. The patient is a female with progressive ataxia and neuropathy, dysarthria, and cerebellar atrophy. She was referred for genetic testing of hereditary cerebellar ataxias due to her brother's (deceased) diagnosis with the same condition and the presence of a variant of unknown significance in *TTBK2* (SCA11) (Brandão *et al*, 2013). Written informed consent for investigation and publication were obtained. The study was performed in line with the principles of the Declaration of Helsinki. Genetic analysis was performed as part of routine diagnostic activity at the Center for Predictive and Preventive Genetics (CGPP), Porto, Portugal.

Briefly, genomic DNA was extracted from the peripheral blood using QIAamp® DNA Blood Mini Kit (Qiagen, Hilden, Germany). Common autosomal dominant ataxias of triplets expanded repeats [SCA1, SCA2, SCA3/MJD, SCA6, SCA7, SCA17, dentatorubral pallidoluysian atrophy (DRPLA)], but also SCA5, SCA8, SCA15, as well as common recessive ataxias [Friedreich's ataxia, ataxia with oculomotor apraxia (AOA) 1 and 2] were excluded by conventional methodologies. Since the clinical phenotype matched SCA11, *TTBK2* exons and intronic flanking regions were genotyped by PCR. In brief, standard PCR amplifications were performed using HotStar Taq master mix (Qiagen, Hilden, Germany) and purified with Exo/SAP (GRiSP, Porto,Portugal). The genomic regions of the repeats were amplified using fluorescently labeled primers. Genotyping was then performed by Sanger sequencing using Big Dye Terminator Cycle Sequencing v1.1 (Applied Biosystems, Foster City, CA, USA), or fragment analysis, in an ABI 3130xl Genetic Analyzer (Applied Biosystems, Foster City, CA, USA). Homoallelic cases were further tested by repeat-primed PCR followed by fragment analysis.

**Subcellular Fractionation**

For subcellular protein fractionation, FLAG-TTBK2-WT and -L209F cells were washed and scraped from the plate in ice-cold 1x PBS. The whole cell extract (corresponding to the total fraction) was centrifuged at 500 *g* for 3 minutes and the supernatant was discarded. Subcellular protein extraction was then performed, using the Subcellular Protein Fractionation Kit for cultured cells (Thermo Fisher Scientific, Waltham, MA, USA), according to the manufacturer’s instructions. Subcellular fractions corresponding to the cytoplasm, membranes, soluble nuclear, and chromatin-bound nuclear extracts were isolated. A fraction of the whole cell extract was collected and sonicated for 10 sec intermittently with a power of 60 W (Branson sonifier 250; ThermoFisher Scientific, Waltham, MA, USA).

**Immunofluorescence**

FLAG-TTBK2-WT and -L209F cells were fixed using 4% paraformaldehyde/4% sucrose (Merck-Millipore, Darmstadt, Germany) for 20 minutes, and permeabilized/blocked in microscopy buffer (1% BSA, 0.1% saponin, and 0.02 % NaN3) for 1h. Then, cells were incubated with the primary antibodies for 3h at 4ºC followed by incubation with the secondary antibodies for 1 hour at RT. After washing with PBS, cells were stained with Hoechst 33342 (1:10.000; Invitrogen, ThermoFisher Scientific, Waltham, MA, USA) for 5 minutes to label the nucleus. Preparations were mounted with ProLong™ Gold Antifade Mountant (Invitrogen, ThermoFisher Scientific, Waltham, MA, USA) and visualized using an epifluorescence Zeiss Axio Imager Z1 microscope equipped with an Axiocam MR3.0 camera and Axiovision 4.7 software (Carl Zeiss, Oberkochen, Germany).

**Supplementary figures and tables**

**Figure S1** TTBK2-L209F variant does not affect cell viability. **(a)** Luminescence cell viability assay of FLAG-TTBK2-WT and -L209F cells. **(b)** Protein analysis of caspase-3 levels in FLAG-TTBK2-WT and -L209F cells by immunoblotting using an antibody against caspase-3 and cleaved fragments. Ponceau S staining was used as loading control. Quantification data are presented in percentage as the mean ± SD of at least three independent experiments; non-significant (ns) compared with the WT cell line (t-test in (a) and Mann-Whitney U test in (b)).

**
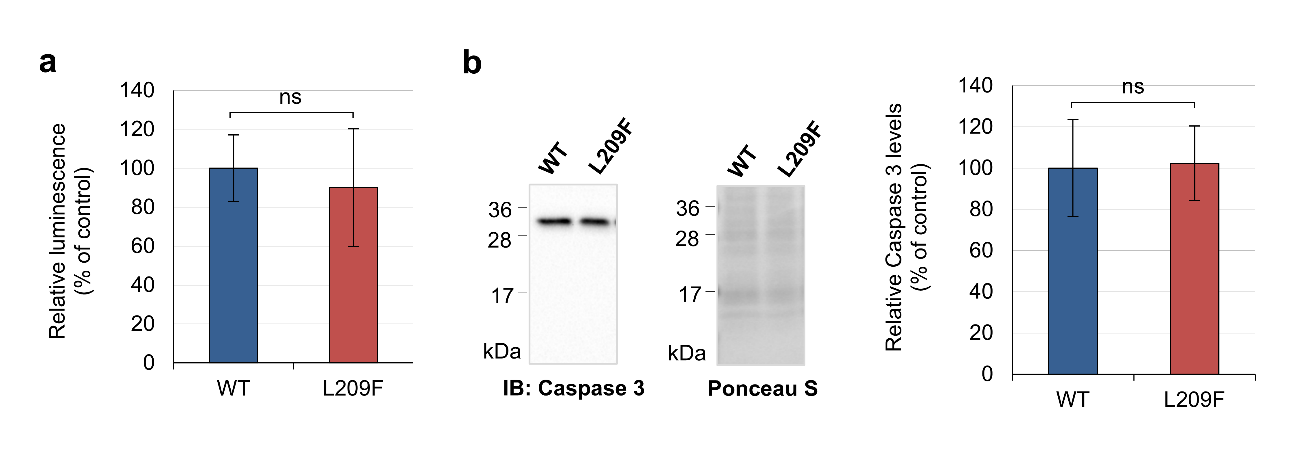
**

**Figure S2** TTBK2 proteins seem to localize mainly in the cytoplasm. **(a)** Immunofluorescence showing the localization of TTBK2 and CEP-97 in FLAG-TTBK2-WT (WT) and FLAG-TTBK2-L209F (L209F) cells probed with anti-FLAG and anti-CEP97 primary antibodies and Alexa Fluor 488 (green) and 568 (red) conjugated secondary antibodies. The nucleus was stained with Hoechst 33342 (blue). Photographs were acquired using a Zeiss Axio Imager Z1 microscope. Bars, 10 μm. **(b)** Subcellular fractionation of FLAG-WT and FLAG-L209F cells. Presence of TTBK2 with the anti-FLAG antibody was evaluated in each fraction, as well as fractions markers: β-actin (total fraction, T), caspase 3 (cytoplasmic fraction, Cp), GM130 (membrane fraction, Mb), HDAC2 (soluble nuclear and chromatin-bound fractions, SN and Ch), and vimentin (cytoskeletal fraction, Ck). The localization of CEP97, a centrosomal protein, is also shown. Ψ identifies an unspecific band.


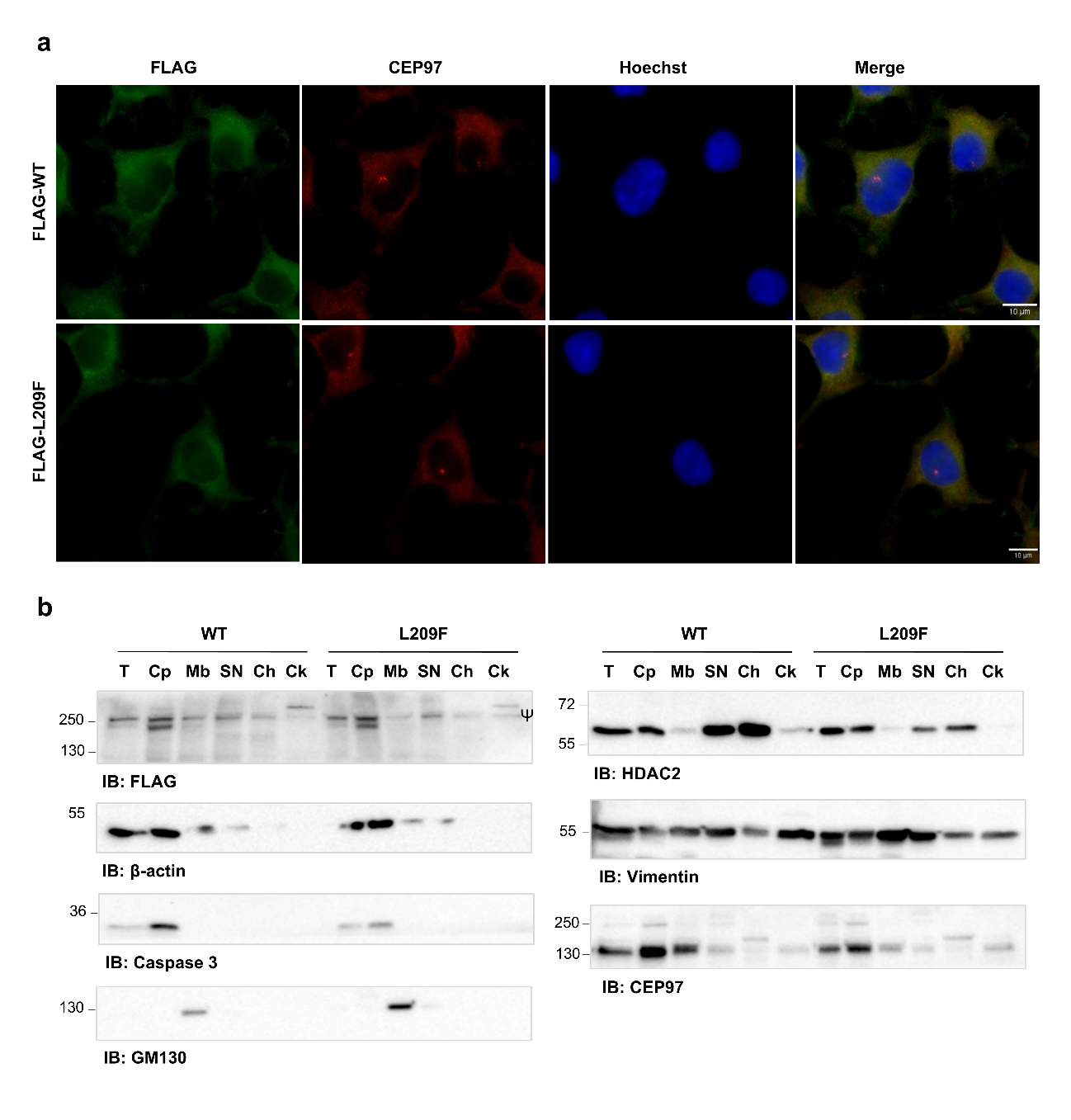


**Figure S3** KIF2A mRNA levels were unaltered in FLAG-TTBK2-L209F cells. Analysis of KIF2A mRNA levels in FLAG-TTBK2-WT (WT) and FLAG-TTBK2-L209F (L209F) by qPCR and normalized towards ACTB levels. Quantification data are presented in fold change as the mean ± SD of three independent experiments; non-significant (ns) compared with the WT cell line (t-test).


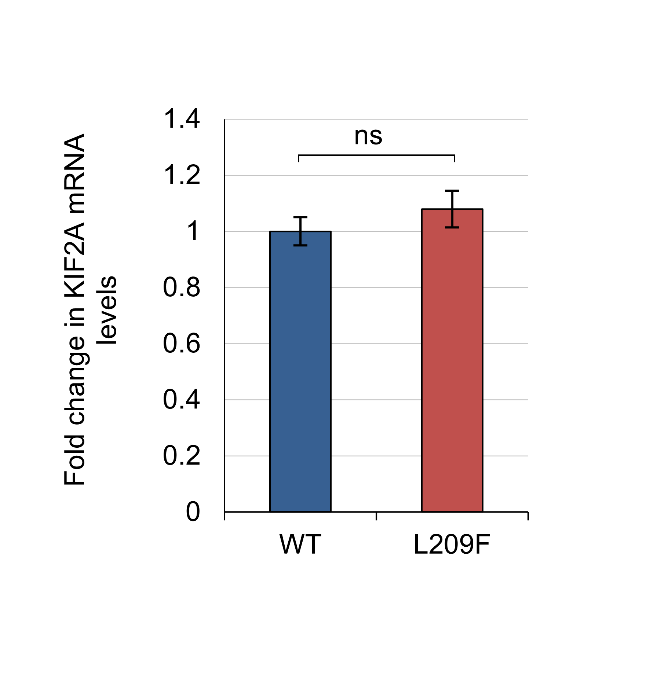


**Figure S4** TTBK2-L209F variant shows impaired kinase activity toward TDP-43, similar to kinase-dead (KD) and SCA11 (R444fs*5) mutants. Analysis of TDP-43 Ser409/410 protein levels (pTDP-43) in HEK293T cells transfected with either empty vector EGFP, EGFP-TTBK2-WT, EGFP-TTBK2-L209F, EGFP-TTBK2-F287S, EGFP-TTBK2-R444fs*5 or EGFP-TTBK2- KD. The ratio pTDP-43/total TDP43 is shown in the graphic. Immunoblotting was performed with anti-TDP-43 Ser409/410, anti-TDP-43 and anti-EGFP antibodies. Quantification data are presented in percentage as the mean ± SD of three independent experiments; non-significant (ns), **P*≤0.05 compared with empty vector transfected cells (one-way ANOVA/Tukey).


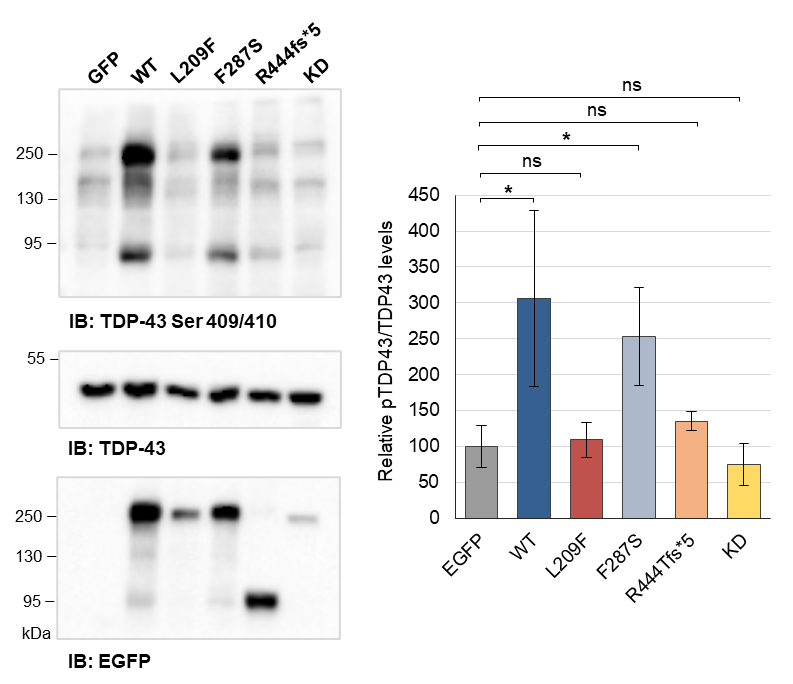


**Figure S5** Overview of the phosphoprotein enrichment protocol used to identify 110 differentially expressed proteins (DEPs) between FLAG-TTBK2-L209F and -WT cell lines. (a) Schematic representation of the MS/MS protocol and phosphoproteomics data analysis. (b) Volcano plot highlighting both up and down regulated DEPs, with protein names labeled for those with p-value<0.001 (https://www.bioinformatics.com.cn/srplot).


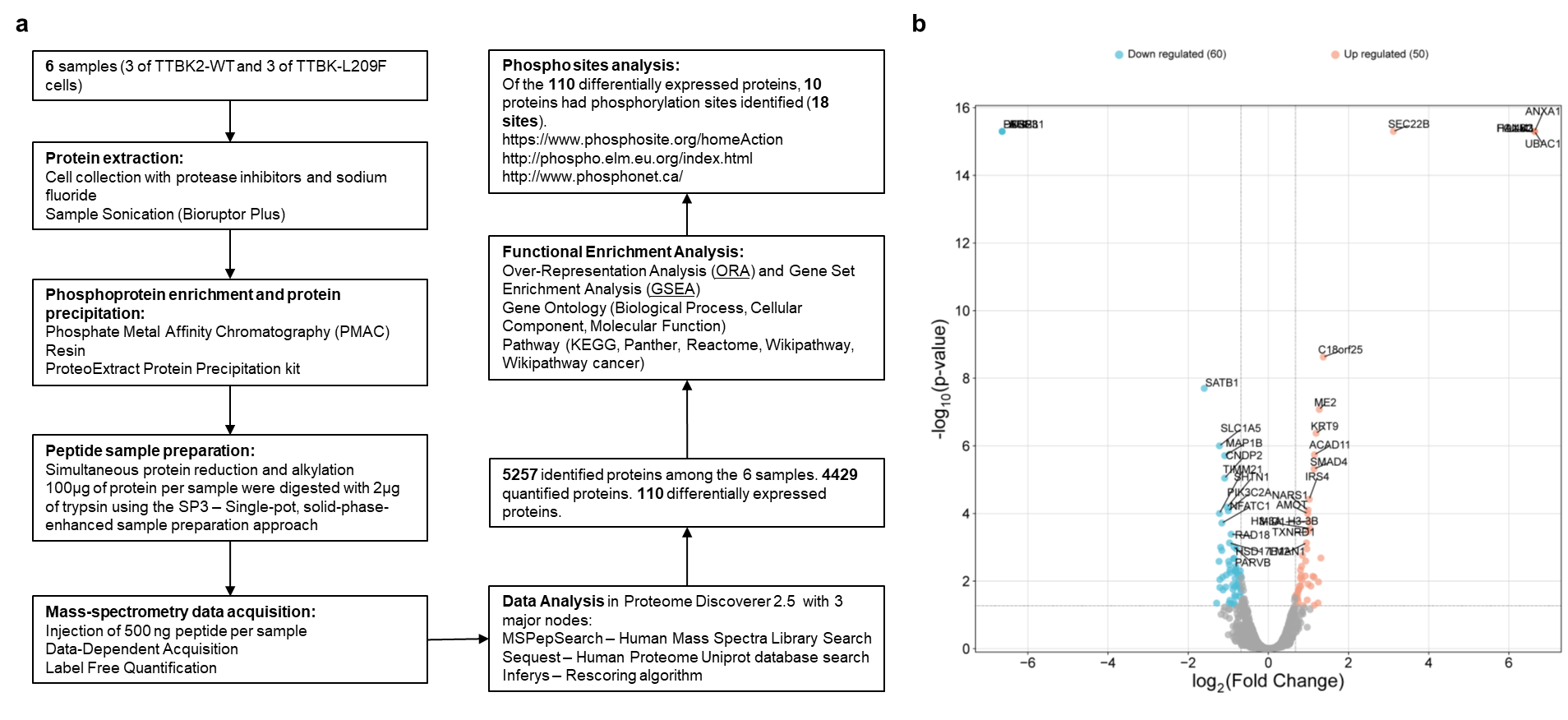


**Figure S6** The mRNA levels of transcription and autophagy-related genes are affected in FLAG-TTBK2-L209F cells. Analysis of SMAD2, p62, LC3B, beclin-1, ULK1 and raptor mRNA levels in FLAG-TTBK2-WT (WT) and FLAG-TTBK2-L209F (L209F) by qPCR and normalized towards ACTB levels. Quantification data are presented in fold change as the mean ± SD of at least three independent experiments; non-significant (ns), **P*≤0.05 and ***P*≤0.01 compared with the WT cell line (t-test, except for p62 and LC3B data where the Mann-Whitney U test was used).


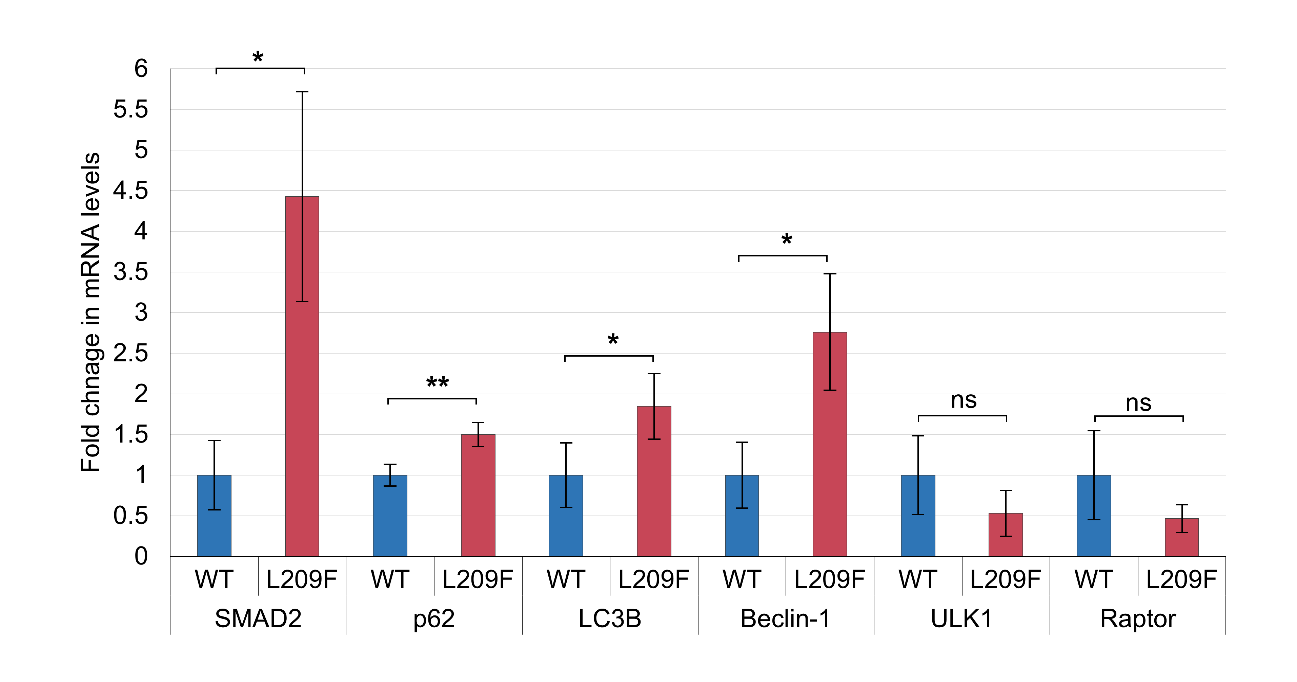


**Figure S7** TTBK2-L209F does not seem to affect proteasome activity. Luminescence assays were employed to quantify the activities of cell proteases, including chymotrypsin, trypsin, and caspase, in both FLAG-TTBK2-WT and -L209F cells. Both cell lines were also treated with MG132 as a control condition for proteasome inhibition (reflected in severely reduced luminescence levels). Data are presented in percentage as the mean ± SD of three independent experiments; non-significant (ns) compared with the WT cell line (t-test for chymotrypsin and Mann-Whitney U test for trypsin and caspase data).


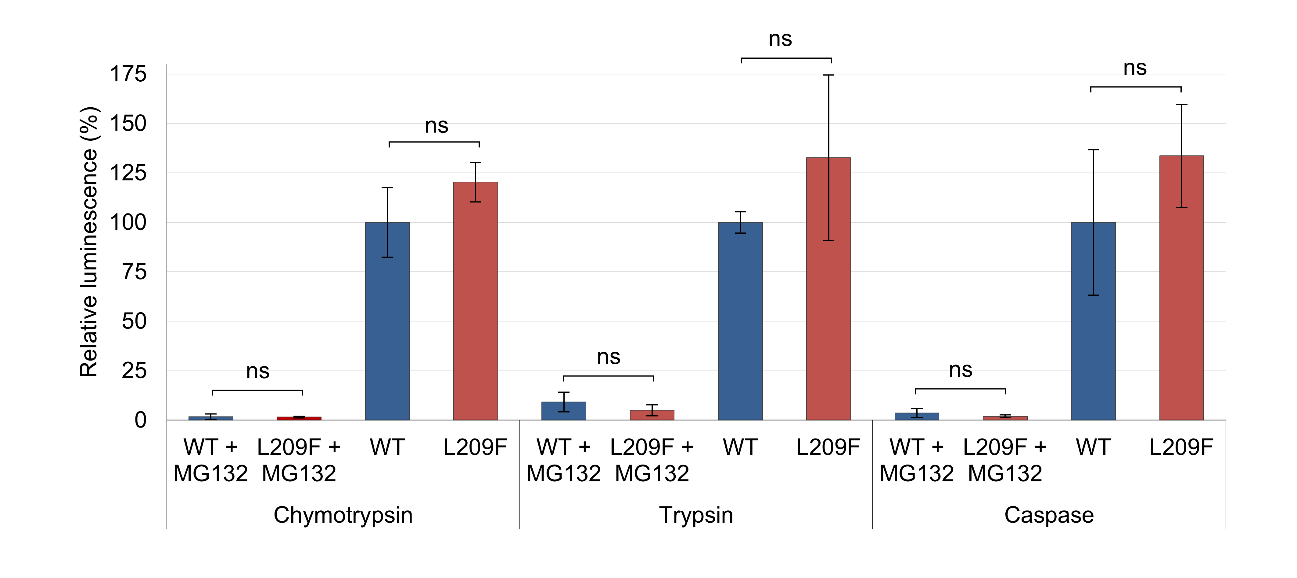


**Table S1** List of antibodies and dilutions used in Western blot and immunofluorescence protocols.

| **Antigen** | **Description** | **Manufacturer** | **Reference** | **Dilution** |
| --- | --- | --- | --- | --- |
| **Western blot - primary antibodies** | | | | |
| EGFP | mouse monoclonal | Abnova | MAB1765 | 1:3000 |
| FLAG | mouse monoclonal | Sigma-Aldrich | F1804 | 1:1000 |
| β-actin | mouse monoclonal | Sigma-Aldrich | A5441 | 1:30.000 |
| GM130 | mouse monoclonal | BD Biosciences | 610822 | 1:1000 |
| Caspase 3 | mouse monoclonal | Cell Signalling | 9668 | 1:1000 |
| HDAC2 | mouse monoclonal | Santa Cruz | sc-9959 | 1:1000 |
| HDAC6 | rabbit polyclonal | Proteintech | 12834-1-AP | 1:500 |
| Vimentin | rabbit polyclonal | Proteintech | 10366-1-AP | 1:30.000 |
| KIF2A | rabbit polyclonal | Proteintech | 13105-1-AP | 1:2000 |
| α-Tubulin | mouse monoclonal | Santa Cruz | sc-5286 | 1:10.000 |
| acetylated-α-tubulin | mouse monoclonal | Sigma | T7451 | 1:10.000 |
| TDP-43/TARDBP | rabbit polyclonal | Proteintech | 10782-2-AP | 1:30.000 |
| Phospho (S409/410)-TDP-43 | rabbit polyclonal | Proteintech | 22309-1-AP | 1:1000 |
| p62/SQSTM1 | mouse monoclonal | Proteintech | 66184-1-Ig | 1:1000 |
| LC3B | rabbit polyclonal | Cell Signalling | 2775 | 1:1000 |
| ULK1 | rabbit monoclonal | Cell Signalling | 8054 | 1:1000 |
| Phospho (Ser555)-ULK1 | rabbit monoclonal | Cell Signalling | 5869 | 1:1000 |
| Beclin1 | rabbit monoclonal | Cell Signalling | 3495 | 1:1000 |
| Phospho (Ser93)-Beclin1 | rabbit monoclonal | Cell Signalling | 14717 | 1:1000 |
| Raptor | rabbit monoclonal | Cell Signalling | 2280 | 1:1000 |
| Phospho (Ser792)-Raptor | rabbit polyclonal | Cell Signalling | 2083 | 1:1000 |
| SMAD2 | rabbit monoclonal | Cell Signalling | 5339 | 1:2000 |
| Phospho (Ser465/467)-SMAD2 | rabbit monoclonal | Cell Signalling | 3108 | 1:1000 |
| CEP97 | rabbit polyclonal | Proteintech | 22050-1-AP | 1:5000 |
| **Immunofluorescence - primary antibodies** | | | | |
| FLAG | mouse monoclonal | Proteintech | 66008-3-Ig | 1:200 |
| CEP97 | rabbit polyclonal | Proteintech | 22050-1-AP | 1:200 |
| **Western blot - secondary antibodies** | | | | |
| HRP-conjugated IgG | Goat anti-mouse | Santa Cruz | sc-2005 | 1:5000 |
| HRP-conjugated IgG | Goat anti-rabbit | Merck-Millipore | 401393 | 1:10.000 |
| **Immunofluorescence - secondary antibodies** | | | | |
| Alexa fluor 568 conjugated IgG | Goat polyclonal anti-rabbit | Thermo Fisher Scientific | A-11011 | 1:200 |
| Alexa fluor 488 conjugated IgG | Goat polyclonal anti-mouse | Thermo Fisher Scientific | A-11029 | 1:200 |

HRP - horseradish peroxidase

**Table S2** Oligonucleotides used to amplify and check for alterations in off-target sequences of CRISPR/Cas9 experiments.

| **Mismatches** | **Gene** | **Off-target Sequence** | **PCR Primers (5'-3')** |
| --- | --- | --- | --- |
| **3xFLAG-TTBK2-WT cell line** | | | |
| 4 | *DNAJC7* | ATCAGAGATTTACAATGAGTAGG | GCCTCCTGAGTTCACACCAT |
|  |  |  | TTGTTGTACTGCTATCAAATACCAT |
|  | *TMEM220* | ATTACTGTCTTGCAGTGAGTTGG | GGAATCCAGTTGGTGGAAGA |
|  |  |  | ACCCTGCAGAAAGTCGTCTG |
|  | *SCHIP1* | ATCACTGAATTGCAGTGGGTGGG | TCCATGTCATTGATATTATTGATGTTC |
|  |  |  | TGTAGCTGCCCAATAGTCATGT |
|  | *MARCH3* | AAAAGAGTATTGCAATCAGTGGG | CGCGTCCATTACTCAAGGAG |
|  |  |  | AGATGGGAGTGAGAACAGCAA |
|  | *SEMA5A* | ATGAGTCTTTTGCAATGACTTGG | TCGGCTGGATTTTCATCATC |
|  |  |  | CACAGTGATGGACAAACACAACT |
|  | *CSMD3* | ATCAGTGTATACCCATGAGATGG | TTGCATTGAAACATTTTTGTGA |
|  |  |  | TGGAGCAAAGGAAAGCAATAG |
|  | *PAGE5* | ATCAGGGTATTGCACCTAGTGGG | GTCTTTAGATTTATGATTCGATGCAC |
|  |  |  | AAGCCTCCGTCAAAATGGTA |
|  | *PAGE2B* | ATCAGGGTATTGCACCTAGTGGG | TGAGCTAGTGTACACGCACTGAT |
|  |  |  | GGCTGAACAAAGCATACAGG |
|  | *PAGE2* | ATCAGGGTATTGCACCTAGTGGG | CTTTAGATGTGTGATTCGATGC |
|  |  |  | CTTGTCCCTTTTATACTCATTTGTTC |
| **3xFLAG-TTBK2-L209F cell line** | | | |
| 4 | *SLC7A2* | TCAAGATGAATAAGGACCAAGGG | ACTGTTTGCTGGGAGCATTA |
|  |  |  | TAATCAGGTGTAGGCCAGAGA |
|  | *CLYBL* | ACATGCTGAATAGGGAACAATGG | CAGGACTCCCTCAGTCATAAAC |
|  |  |  | CTTAAGGAACAGTCGTCCAGAG |
|  | *TTBK1* | GCATGTAGAAGAGGGACCACAGG | GGAAGCACACACGGTAGTT |
|  |  |  | CATCCGGTGCTCATACTTCTC |
|  | *TMEM181* | GCATGTAGAATATGTACCATAGG | TTCAACTTGTCTTCCCTGGTAT |
|  |  |  | CTGGTCATCTTCCAGGTTCTC |
| 3 | *TAFA1* | ATATGTAGAATAACAACCAATGG | AACCAGCAGTCTTTGGTGAA |
|  |  |  | TGAATCCAACTGTGCCTGAA |
|  | *PIBF1* | AAATGTAGCATAAGGACAAATGG | TCCTTCAGAGCAAACCATCAG |
|  |  |  | CCAACAGTGAGGAACTGATGAT |
|  | *NEBL* | ACATGTACAATAAGGAAAAATGG | TACCAGTTCCCTGAGGCATT |
|  |  |  | AACTAGCGCAGTCTAATTGTGTA |
|  | *STAT5B* | ACATGAAGAATAAGGGCCCAGGG | GACCAAGGAGAACCTCGTG |
|  |  |  | ACATCTGCTTTGCTCTATTTCC |
|  | *DEPDC5* | GCAGGTAGAATCAGGACCAATGA | GCTTACAAGGGTAGAACAGTAGG |
|  |  |  | ATTCAGAGGGTCCAGAAATCAC |
|  | *TCF4* | ACCTTTTGAATAAGGACCAAAGA | GGTTCAGGCTTTCAGAGCTAAT |
|  |  |  | CATATCACACGAGGGCAGAAT |
| 2 | *KALRN* | ACATATAGAATAAGGACCAGAAG | TGACATGAGGAAGAGCTTGTG |
|  |  |  | GTGGAGGCTCTGGATGTATAAG |

**Table S3** Oligonucleotides used to amplify and confirm TTBK2 coding exonic sequence and flanking introns.

| **Exon/Intron** | **PCR Primers (5'-3')** |
| --- | --- |
| Exon 2 | TATTAGTAGTGCTTCCTGATGCTG |
|  | AGGGTATAAACAGAGCTTAATCATAG |
| Exon 3 | AAGAGATCAATTGCTTTCAAGTTAG |
|  | GAATGTAATTAAGGTTCAGGAAGATAC |
| Exon 4 | CTGGGAGGAGAAGAGGTAGGTAG |
|  | CTGTTTAAATACAGTTCCATCTGC |
| Exon 5 | AATGGTAAGTGAGTAGTTTCCCAG |
|  | CCACAGCAAATACACAAAGC |
| Exon 6 | TGGAACATGTGTGTTATATAGAG |
|  | TCATTAAATGTATATGCCACTTC |
| Exon 7 | TTTCTTCCATTTACTTCATGGTTC |
|  | GCAAGACTCTGTCTCAACAAATATAC |
| Exon 8 | GCTTGCTTCCCTTATTTCTACC |
|  | AAGCTAAGAGGGAGGGCTATAA |
| Intron 8 | GCTTGCTTCCCTTATTTCTACC |
|  | TGCTGAATTCTGGAGGGAGA |
| Exon 9 | GAGCTCAGTTCTTGCTTTATGC |
|  | TGAAGTTAAGTCCATCCAGAGG |
| Exon 10 | GCAATTTGAGCCATCAGTTT |
|  | TCTGAGGGAATCTGGATGAA |
| Exon 11 | AACCTCCGCCTTCTAGATTC |
|  | CCCTCATGAATGAGAGTGGT |
| Exon 12 | TTTGAAAGGCTTCAATGATTC |
|  | GGAGCTGAATAGACTCAGTGTTAC |
| Exon 13 | GAACTGAGCTCCATCTTCCA |
|  | TGAAGCAGCAGTAGGAGGAC |
|  | TAGGTCCTTGGGCAGAAAAT |
|  | CAGTGGCCATCAATGTTAGA |
| Exon 14 | TGTGTTCTTCCTATAAATTAGAATTGG |
|  | GTCTTGATTTGGCACCACAC |
|  | TTAAGTAGAGGGCAGCATTG |
|  | TTTTGCAGAAACTGGAGAGG |
|  | GGATATGGTTAGGTCATCCTTTG |
|  | TGGTGGACTACAATAAAGCAG |
| Exon 15 | AATTTGTCCAGGGTCATACAAC |
|  | TTTACATAGAAAGTACAGGGACACAC |

**Table S4** Oligonucleotides used for qPCR experiments.

| **Target** | **qPCR Primers (5'-3')** |
| --- | --- |
| TTBK2 (Exon4/5) | TGTAGATTTATTGGCTGTGG |
|  | TGGTACTAATGGTGAATGTG |
| TTBK2 (Exon 8/9) | TGGAGTTTGTGGTTGGTCAG |
|  | TGAGCCTGTGGTCATATCTCT |
| KIF2A | CCTGACCTTGTTCCTGATG |
|  | CTGAACCAACCACTCTATTATCT |
| SMAD2 | GAGAGTTGAGACACCAGTT |
|  | GAGTGAGTATAGTCATCCAGAG |
| p62/SQSTM1 | ATGACATCTTCCGAATCTAC |
|  | CATCGCAGATCACATTGG |
| LC3B | CGAGAGCAGCATCCAACC |
|  | ACATGGTCAGGTACAAGGAACT |
| Beclin-1 | GAGACCCAGGAGGAAGAG |
|  | GATGAATCTGCGAGAGACA |
| ULK1 | AAGAAGAACCTCGCCAAG |
|  | TAGACAGAATTAGCCATTTCCT |
| Raptor | ACAAGAACTACACGCAGTA |
|  | CATTGGAGCAGTCGTAGA |
| ACTB | GCACTCTTCCAGCCTTCCTTC |
|  | GTGATCTCCTTCTGCATCCTGTC |

**Table S5** *TTBK2* missense variants nucleotide conservation and pathogenicity prediction scores.

| **Reference** | Galatolo *et al*., 2021 | Iqbal *et al*., 2017 | Brandão *et al*, 2013; this study | Coutelier *et al*., 2017 |
| --- | --- | --- | --- | --- |
| **dbSNP** | rs778218227 | rs200821440 | rs1595936604 | rs199517778 |
| **cDNA** | c.239T>A | c.245G>C | c.625C>T | c.659T>C |
| **Protein** | **p.Phe80Tyr** | **p.Gly82Ala** | **p.Leu209Phe** | **p.Val220Ala** |
| **Main clinical phenotype /**  **Age at onset** | Spinocerebellar ataxia / 73 years old | Complex ataxia; spastic ataxia / NA | Progressive ataxia, neuropathy, dysarthria, cerebellar atrophy / 48 years old | Ataxia / NA |
| **gnomAD v4.1.0 allele frequency** | 1.239E-06 | 2.658E-04 | 1.86E-06 | 1.425E-05 |
| **gnomAD v4.1.0 allele count** | 2 | 429 | 3 | 23 |
| **Conservation tools** | | | | |
| **GERP++** | 5.31 | 5.31 | 5.57 | 5.57 |
| **PhyloP100way (vertebrate)** | 8.517 | 7.211 | 10.003 | 5.389 |
| **PhyloP30way (mammalian)** | 1.312 | 1.026 | 1.176 | 1.312 |
| **PhyloP17way (primate)** | 0.691 | 0.549 | 0.676 | 0.756 |
| **PhastCons100way (vertebrate)** | 1 | 1 | 1 | 1 |
| **PhastCons30way (mammalian)** | 1 | 1 | 1 | 1 |
| **PhastCons17way (primate)** | 0.998 | 0.999 | 0.999 | 0.997 |
| **Pathogenicity tools** | | | | |
| **CADD Score** | 26.1 | 24.6 | 28 | 20.3 |
| **MutationTaster** | Disease-causing (1) | Disease-causing (1) | Disease-causing (1) | Disease-causing (0.999) |
| **Mutation assessor** | Neutral (0.800) | Neutral (0.505) | Medium (1.95) | Neutral (0.495) |
| **MutPred2** | Pathogenic (0.823) | Pathogenic (0.917) | Pathogenic (0.776) | Benign (0.435) |
| **FATHMM** | Tolerated (2.09) | Tolerated (1.93) | Tolerated (1.89) | Tolerated (2.14) |
| **FATHMM-MKL** | Damaging (0.979) | Damaging (0.975) | Damaging (0.971) | Damaging (0.920) |
| **FATHMM-XF** | Damaging (0.840) | Damaging (0.883) | Damaging (0.845) | Neutral (0.483) |
| **DEOGEN2** | Tolerated (0.340) | Tolerated (0.355) | Tolerated (0.097) | Tolerated (0.091) |
| **EIGEN** | Uncertain (5.141) | Uncertain (5.765) | Pathogenic (13.961) | Benign (2.233) |
| **SIFT** | Deleterious (0.05) | Deleterious (0.04) | Deleterious (0.01) | Tolerated (0.79) |
| **Provean** | Damaging (-2.86) | Damaging (-5.71) | Damaging (-3.87) | Neutral (-1.19) |
| **REVEL** | Benign (0.30) | Benign (0.42) | Pathogenic (0.638) | Benign (0.081) |
| **Primate AI** | Damaging (0.856) | Tolerated (0.779) | Damaging (0.905) | Damaging (0.885) |
| **MetaLR** | Tolerated (0.060) | Tolerated (0.107) | Tolerated (0.228) | Tolerated (0.039) |
| **MetaRNN** | Damaging (0.617) | Tolerated (0.427) | Damaging (0.763) | Tolerated (0.110) |
| **Polyphen** | Possibly damaging (0.77) | Possibly damaging (0.678) | Probably damaging (0.999) | Benign (0.031) |
| **BayesDel addAF** | Tolerated (-0.035) | Tolerated (-0.192) | Damaging (0.244) | Tolerated (-0.106) |
| **BayesDel noAF** | Tolerated (-0.192) | Tolerated (-0.156) | Damaging (0.114) | Tolerated (-0.391) |
| **ClinPred** | Damaging (0.928) | Tolerated (0.263) | Damaging (0.995) | Tolerated (0.361) |
| **LIST-S2** | Damaging (0.967) | Damaging (0.980) | Damaging (0.991) | Tolerated (0.748) |
| **VEST4** | Deleterious (0.804) | Deleterious (0.93) | Deleterious (0.773) | Tolerated (0.429) |
| **DANN** | Deleterious (0.953) | Deleterious (0.953) | Deleterious (0.999) | Deleterious (0.915) |
| **Nº software** | 14/22 | 11/22 | 19/22 | 5/22 |

NA, not available. Nº software, number of sowftare predicting deleterious effects.

**Table S6** Functional enriched categories of the 110 DEP (+, upregulated; -, downregulated) in FLAG-TTBK2-L209F cells using the WebGestalt ORA enrichment method. The Gene Ontology (GO) categories [Biological Process (BP), Cellular Component (CC) and Molecular Function (MF)], pathway databases (KEGG, Panther, Reactome and Wikipathway), and disease functional databases (Disgenet and GLAD4U) were considered for analysis. P≤0.05; Benjamini–Hochberg approach. Values were rounded to 2 decimal places. The weighted set cover redundancy reduction method was included as a post-processing step.

| **DEP** | **Gene Set** | **Description** | **Gene Symbol** | **Size** | **Expect** | **Ratio** | **P-value** | **FDR** |
| --- | --- | --- | --- | --- | --- | --- | --- | --- |
| + | R-HSA-1266738 | Developmental Biology | *PIAS2, SLIT2, KRT1, KRT10, GAP43, PFN2, KRT9, H3F3B, H3F3A, SMAD4, SMAD2, HIST2H3D, HIST2H3C, HIST2H3A, PIK3R3* | 1074 | 2.89 | 5.20 | 8.30E-08 | 4.41E-04 |
|  | GO:0051253 | Negative regulation of RNA metabolic process | *HEXIM1, ZIC2, H3F3A, MECOM, FOXM1, SMAD4, SQSTM1, ARID5B, SMAD2, HIST2H3C, NEDD4L, DHX36, MBD2, MBD1* | 1295 | 3.48 | 4.02 | 5.25E-06 | 4.29E-03 |
|  |  |  |  |  |  |  |  |  |
|  | GO:0072359 | Circulatory system development | *SLIT2, HEXIM1, ANXA1, KRT1, SMAD4, SHB, SMAD2, AMOT, BBS7, PIK3R3, DHX36, MBD2* | 1026 | 2.76 | 4.35 | 1.31E-05 | 7.11E-03 |
|  |  |  |  |  |  |  |  |  |
|  | GO:0010629 | Negative regulation of gene expression | *CTIF, SLIT2, HEXIM1, ANXA1, KRT1, SMAD4, SHB, SMAD2, AMOT, BBS7, PIK3R3, DHX36, MBD2* | 1733 | 4.66 | 3.22 | 3.25E-05 | 1.30E-02 |
|  |  |  |  |  |  |  |  |  |
|  | GO:0010639 | Negative regulation of organelle organization | *MID1, SEC22B, SLIT2, PFN2, LMAN1, H3F3A, SMAD4* | 371 | 1.00 | 7.02 | 5.71E-05 | 2.06E-02 |
|  |  |  |  |  |  |  |  |  |
|  | GO:0001667 | Ameboidal-type cell migration | *SLIT2, ANXA1, PFN2, ARID5B, AMOT, PIK3R3, AMOTL2* | 381 | 1.02 | 6.83 | 6.75E-05 | 2.28E-02 |
|  |  |  |  |  |  |  |  |  |
|  | R-HSA-2262752 | Cellular responses to stress | *H3F3B, H3F3A, TXNRD1, HIST2H3D, HIST2H3C, HIST2H3A, TNIK* | 426 | 1.15 | 6.11 | 1.35E-04 | 4.11E-02 |
| - | R-HSA-446353 | Cell-extracellular matrix interactions | *LIMS1, ILK, PARVB, PARVA* | 18 | 0.05 | 73.96 | 2.17E-07 | 4.61E-03 |

**Table S7** Functional enrichment analysis using WebGestalt GSEA enrichment approach. The Gene Ontology (GO) categories [Biological Process (BP), Cellular Component (CC) and Molecular Function (MF)], pathway databases (KEGG, Panther, Reactome and Wikipathway), and disease functional databases (Disgenet and GLAD4U) were considered for analysis. P≤0.05; Benjamini–Hochberg approach. Values were rounded to 2 decimal places. The weighted set cover redundancy reduction method was included as a post-processing step. LEN – leading edge number; ES – enrichment score; NES – normalized enrichment score. The GSEA information before the processing step of redundancy reduction may be available upon request.

| **Gene Set** | **Description** | **Gene Symbol** | **Size** | **LEN** | **ES** | **NES** | **P-Value** | **FDR** |
| --- | --- | --- | --- | --- | --- | --- | --- | --- |
| hsa05034 | Alcoholism | *H2AFV, HDAC4, BRAF, SOS1, H3F3B, H3F3A, ARAF, HDAC6, HIST2H2BF, HIST1H3A, HIST1H3B, HIST1H3C, HIST1H3D, HIST1H3E, HIST1H3F, HIST1H3G, HIST1H3H, HIST1H3I, HIST1H3J, HIST4H4, HIST1H4A, HIST1H4B, HIST1H4C, HIST1H4I, HIST1H4D, HIST1H4E, HIST1H4F, HIST1H4H, HIST1H4J, HIST1H4K, HIST1H4L, HIST2H4A, HIST2H4B, HIST2H3D, HIST2H3C, HIST2H3A, HIST2H2BE, HDAC1, MAPK3, HDAC2* | 62 | 40 | 0.49 | 4.42 | 0.00 | 0.00 |
| WP2369 | Histone Modifications | *H3F3B, H3F3A, HIST1H3A, HIST1H3B, HIST1H3C, HIST1H3D, HIST1H3E, HIST1H3F, HIST1H3G, HIST1H3H, HIST1H3I, HIST1H3J, KMT2B, HIST4H4, HIST1H4A, HIST1H4B, HIST1H4C, HIST1H4I, HIST1H4D, HIST1H4E, HIST1H4F, HIST1H4H, HIST1H4J, HIST1H4K, HIST1H4L, HIST2H3D, HIST2H3C, HIST2H3A, SUV39H2, SET, SMYD5, EED* | 50 | 32 | 0.43 | 3.57 | 0.00 | 0.00 |
| R-HSA-2559583 | Cellular Senescence | *PHC3, H2AFV, MAPKAPK2, BMI1, TP53, AGO1, RPS6KA3, TNIK, H3F3B, H3F3A, ERF, PHC2, ANAPC7, CDKN2A, RPS6KA1, HIST1H3A, HIST1H3B, HIST1H3C, HIST1H3D, HIST1H3E, HIST1H3F, HIST1H3G, HIST1H3H, HIST1H3I, HIST1H3J, HIST4H4, HIST1H4A, HIST1H4B, HIST1H4C, HIST1H4I, HIST1H4D, HIST1H4E, HIST1H4F, HIST1H4H, HIST1H4J, HIST1H4K, HIST1H4L, HIST2H4A, HIST2H4B, HIST2H3D, HIST2H3C, HIST2H3A, HIST2H2BE, RNF2, MAPK3, HIRA, TNRC6B, RELA, MAPK1, HIST1H1E* | 103 | 50 | 0.29 | 3.48 | 0.00 | 0.00 |
| WP4553 | FBXL10 enhancement of MAP/ERK signaling in diffuse large B-cell lymphoma | *H2AFV, H3F3B, H3F3A, HIST1H3A, HIST1H3B, HIST1H3C, HIST1H3D, HIST1H3E, HIST1H3F, HIST1H3G, HIST1H3H, HIST1H3I, HIST1H3J, HIST2H3D, HIST2H3C, HIST2H3A, RNF2, MAPK3, MAPK1, EED, H2AFY2* | 27 | 21 | 0.54 | 3.38 | 0.00 | 0.00 |
| R-HSA-9018519 | Estrogen-dependent gene expression | *H2AFV, MED1, AGO1, POU2F1, H3F3B, H3F3A, HSP90AA1, YY1, HIST1H3A, HIST1H3B, HIST1H3C, HIST1H3D, HIST1H3E, HIST1H3F, HIST1H3G, HIST1H3H, HIST1H3I, HIST1H3J, HIST4H4, HIST1H4A, HIST1H4B, HIST1H4C, HIST1H4I, HIST1H4D, HIST1H4E, HIST1H4F, HIST1H4H, HIST1H4J, HIST1H4K, HIST1H4L, HIST2H4A, HIST2H4B, HIST2H3D, HIST2H3C, HIST2H3A, CREBBP, HIST2H2BE, HDAC1, TNRC6B, USF1, POLR2H, CDK9, CTSD, AGO2, POLR2K, CBFB* | 84 | 46 | 0.32 | 3.37 | 0.00 | 0.00 |
| R-HSA-68875 | Mitotic Prophase | *H2AFV, EMD, SMC4, NEK9, NEK7, H3F3B, H3F3A, NCAPD3, NUP54, NCAPG2, SMC2, RAB2A, HIST1H3A, HIST1H3B, HIST1H3C, HIST1H3D, HIST1H3E, HIST1H3F, HIST1H3G, HIST1H3H, HIST1H3I, HIST1H3J, HIST4H4, HIST1H4A, HIST1H4B, HIST1H4C, HIST1H4I, HIST1H4D, HIST1H4E, HIST1H4F, HIST1H4H, HIST1H4J, HIST1H4K, HIST1H4L, HIST2H4A, HIST2H4B, HIST2H3D, HIST2H3C, HIST2H3A, HIST2H2BE, PRKCA, MAPK3, PLK1, MAPK1, NUMA1, SET* | 95 | 46 | 0.29 | 3.29 | 0.00 | 0.00 |
| R-HSA-157118 | Signaling by NOTCH | *H2AFV, PRKCI, HDAC4, DLGAP5, PBX1, NOTCH2, TP53, AGO1, TLE4, H3F3B, H3F3A, ITCH,PSMD8, YWHAZ, HDAC6, HIST1H3A, HIST1H3B, HIST1H3C, HIST1H3D, HIST1H3E, HIST1H3F, HIST1H3G, HIST1H3H, HIST1H3I, HIST1H3J, PSMF1, HIST4H4, HIST1H4A, HIST1H4B, HIST1H4C, HIST1H4I, HIST1H4D, HIST1H4E, HIST1H4F, HIST1H4H, HIST1H4J, HIST1H4K, HIST1H4L, HIST2H4A, HIST2H4B, HIST2H3D, HIST2H3C, HIST2H3A,CREBBP, HIST2H2BE* | 120 | 45 | 0.24 | 3.12 | 0.00 | 0.00 |
| WP4320 | The effect of progerin on the involved genes in Hutchinson-Gilford Progeria Syndrome | *MBD2, TP53, H3F3B, H3F3A, CBX1, HIST1H3A, HIST1H3B, HIST1H3C, HIST1H3D, HIST1H3E, HIST1H3F, HIST1H3G, HIST1H3H, HIST1H3I, HIST1H3J, HIST2H3D, HIST2H3C, HIST2H3A, HDAC1, HDAC2* | 33 | 20 | 0.45 | 3.03 | 0.00 | 0.00 |
| R-HSA-8878171 | Transcriptional regulation by RUNX1 | *PHC3, H2AFV, PSME4, CTSV, BMI1, OCLN, ARID1B, SMARCC1, AGO1, PTPN11, H3F3B, H3F3A, ITCH, PHC2, PRKCQ, PSMD8, ASH2L, HIST1H3A, HIST1H3B, HIST1H3C, HIST1H3D, HIST1H3E, HIST1H3F, HIST1H3G, HIST1H3H, HIST1H3I, HIST1H3J, PSMF1, KMT2B, HIST4H4, HIST1H4A, HIST1H4B, HIST1H4C, HIST1H4I, HIST1H4D, HIST1H4E, HIST1H4F, HIST1H4H, HIST1H4J, HIST1H4K, HIST1H4L, HIST2H4A, HIST2H4B, HIST2H3D, HIST2H3C, HIST2H3A, CREBBP, HIST2H2BE, RNF2, CSNK2A2, RBBP5* | 139 | 51 | 0.23 | 3.02 | 0.00 | 0.00 |
| WP481 | Insulin Signaling | *EHD2, PIK3R3, RAPGEF1, GSK3B, PRKCI, STXBP4, IRS4, PIK3R1, GSK3A, CBL, PTPN11, RPS6KA3, MAP2K2, SOS1, EIF4E, PRKCQ, PIK3C3, KIF3A, RPS6KA1, SORBS1, PIK3R4, GYS1, PRKCA, MAPK3, GRB10, RHEB, STX4, FOXO3, MAPK1, PFKM, TSC1, STXBP1, PTPN1, MAP3K7, MTOR, KIF5B, MAP3K4, MAP3K2, FLOT1, EHD1, INPP4A, AKT1, MAP4K4, TSC2* | 78 | 44 | 0.28 | 2.85 | 0.00 | 0.00 |
| GO:1903311 | Regulation of mRNA Metabolic Process | *DHX36, SUPT6H, RNF40, MAPKAPK2, RNF20, RBM17, GIGYF2, IGF2BP1, ANGEL2, DHX34, TNPO1, DAZAP1, PUM1, HNRNPA1, XRN1, SON, FXR1, SAMD4B, LARP1, YWHAZ, FMR1, GTPBP1, C9orf78, DIS3, RBM4B, CIRBP, TRA2B, DYRK1A, PRKCA, PARN, VIM, CPSF4, ALKBH5, TNRC6B, EIF4G1, EXOSC9, SRSF10, SRSF7, SET, SRSF2, CDK9, YWHAB, AGO2, EXOSC8, MYEF2, DDX17, IGF2BP2, RBM4, SLTM, SRSF6, HNRNPD, CARHSP1, DCP1A, SART3, SECISBP2, MTOR, RBMX, SUPT5H, AKT1, CDK11B, RBM8A, IWS1, RBM5, EXOSC6, RBM42, RBM15B, FAM172A, CNOT8, NELFE, RBM15, LEO1, CCNT1, NUP214, EXOSC4, TBRG4, WTAP, SNW1, SRSF3, PABPC4, UPF1, APEX1, AHCYL1, SRPK2, PABPC1, SRSF9, YBX1, PRPF19, TRA2A, BARD1, EXOSC7, SAFB2, SREK1, SRSF5, SERBP1, RNASEL, SRPK1, ANP32A, CDC73, SRSF4, RPS27A, METTL16, ZC3H14, HNRNPC, CPSF7, ZC3HAV1, EXOSC2, RBM25, THRAP3, C1QBP, MOV10, SRSF1, RNPS1, PAPOLA, HNRNPA2B1, CWC22, HNRNPU, HSPB1, PTBP1, JMJD6, EXOSC1, U2AF2, RBM7, SNRPA, SNRNP70, METTL14, METTL3, PAF1, SMU1, CTR9, CPSF6, KHDRBS3, PNPT1, NUDT21, SF3B4, PCID2, DDX5, YTHDC1, HNRNPR, SYNCRIP, DHX9, HSF1, SAP18, RBMXL1, EXOSC3, PDE12, PRDX6, ZFP36L2, RBM10, RBM23, NCBP2, RBM3, POLR2G, DCP2, PUM2, FXR2, HNRNPK, YTHDF2, PABPN1, XPO1, RBM19, HNRNPL, KHDRBS1* | 185 | 162 | 0.18 | 2.76 | 0.00 | 0.00 |
| GO:0032147 | Activation of Protein Kinase Activity | *WNK1, RAPGEF1, TRAF4, STK25, MAPKAPK2, BRAF, MALT1, IKBKG, PTPN11, TAB2, MAP2K2, TNIK, STK24, PKN1, KIF14, CCDC88A, MAP3K20, IRAK1, HMGB1, TPX2, LAMTOR3, RIPK1, PRKAR1A, STK26, ECT2, MAPK3, KIDINS220, MAPK1, PAK4, PRKACA, ERP29, DAXX, TAOK2, ABL1, TAB3, TRAF2, ERCC6, PTPN1, MAP3K7, MTOR, MAP3K4, MAP3K2, AKT1, MAP4K4, PAK2, RIPK2, SPAG9, TAOK1, TAB1, DLG1, MARK2, PAK1, CLSPN, EIF2AK2, KARS, NEK4, MAP2K1* | 102 | 57 | 0.23 | 2.70 | 0.00 | 0.00 |
| hsa04360 | Axon guidance | *PIK3R3, GSK3B, SLIT2, CAMK2D, NCK1, DPYSL2, PIK3R1, PTPN11, CAMK2G, NCK2, PARD6B, PPP3CA, DPYSL5, PLCG1, ENAH, CDK5, PRKCA, MAPK3, ABLIM1, MAPK1, PAK4, ROCK1, ABL1, CFL1, ROCK2, NFATC3, PAK2, PAK1, PLXNA1, PLCG2* | 44 | 30 | 0.35 | 2.69 | 0.00 | 0.00 |
| GO:0051493 | Regulation of Cytoskeleton Organization | *CORO1A, SMAD4, GSK3B, DIAPH1, SLIT2, SKA1, NCK1, CAMSAP1, EPS8, MID1, ARPC5, BRAF, PIK3R1, CEP131, LATS1, PFN2, RDX, CDK5RAP2, CHORDC1, NCK2, LIMCH1, CDC42EP4, CEP250, NEXN, CHMP2B, CORO1B, CCDC88A, PXN, NF2, MAPRE2, MAP9, AURKA, TRIOBP, SH3PXD2B, TPX2, ACTR2, WDR1, VANGL2, HDAC6, CDK5, RND3, SPTBN1, FCHSD2, UBXN2B, ECT2, PAFAH1B1, STMN3, PFN1, DYRK1A, MAPK3, MAPRE3, CKAP2, EZR, PSRC1, DSTN, TACC3, PLK1, CTTN, MAPK1, NUMA1, NIN, NES, CDC42EP1, WASF2, ABL2, FHOD3, TRIM27, TSC1, TAOK2, ROCK1, MYCBP2, ABL1, MAP2, ARPIN, FHOD1, CFL1, TMN1, ARHGAP35, WASF1, PPM1E, MTOR, ROCK2, CYLD, CAPN2, AKAP13, DYNLT1, TAOK1, DLG1, TRAF3IP1, SYDE1, MARK2, KIF11, PAK1, TMOD1, NSFL1C, STAG2, CENPJ, ARHGEF2, CETN2, KATNB1, FLNA, ARHGAP17, TUBB4A, HDGFL3, ARPC4, ACTR3, SWAP70, CEP97, CLIP1, HNRNPU, LRP1, WASL, BCAS3, SORBS3, SPTAN1, WIPF2, MYO1C, ARAP1, DCTN1, HAX1, AFAP1, MECP2, CHEK1* | 212 | 123 | 0.16 | 2.64 | 0.00 | 0.00 |
| hsa05203 | Viral carcinogenesis | *PIK3R3, HDAC4, MAPKAPK2, PIK3R1, TP53, IKBKG, VAC14, GTF2E1, PXN, CDKN2A, YWHAZ, HDAC6, HIST2H2BF, GTF2H2, GTF2H2C, HIST4H4, HIST1H4A, HIST1H4B, HIST1H4C, HIST1H4I, HIST1H4D, HIST1H4E, HIST1H4F, HIST1H4H, HIST1H4J, HIST1H4K, HIST1H4L, HIST2H4A, HIST2H4B, HLA-A, CREBBP, HIST2H2BE, RBL2, TBPL2, HDAC1, HLA-B, MAPK3, HDAC2, RELA, MAPK1, PRKACA, YWHAB, HLA-C, USP7, TRAF2, SCRIB, CREB1, GTF2E2, ACTN1, DDX3X, DLG1, RBPJ, EIF2AK2, POLB, CDK1, ACTN4, PSMC1, YWHAE, CHD4, YWHAH, UBE3A, CHEK1, YWHAQ, SRC, PIK3R2, HDAC3, CASP3, VDAC3, STAT3, HNRNPK, SND1, CDC20, CDK4* | 103 | 73 | 0.22 | 2.57 | 0.00 | 0.00 |
| WP51 | Regulation of Actin Cytoskeleton | *PIK3R3, DIAPH1, ARPC5, BRAF, PIK3R1, MYH10, MAP2K2, RDX, SOS1, MSN, PXN, PPP1R12A, PIK3C3, ENAH, PIK3R4, PFN1, MAPK3, ARHGEF6, EZR, MAPK1, PAK4, ARHGEF1, LOC100505585, WASF2, ROCK1, GIT1, CFL1, ARHGAP35, WASF1, ROCK2, PAK2, ACTN1, VCL* | 58 | 33 | 0.27 | 2.50 | 0.00 | 0.01 |
| GO:0044441 | Ciliary part | *ULK3, UNC119B, SQSTM1, BBS7, SCLT1, SSX2IP, KIF5A, CEP131, CENPF, TTC26, CCDC88A, KIF3B, IFT122, PIK3C3, KIF3A, CROCC, MAP1LC3B, ARL13B, PRKAR1A, PIK3R4, ODF2, IFT81, CCDC120, SEPT4, CEP41, EZR, CEP290, PCM1, NIN, PFKM , DYNLL1, PRKACA, GPI, C5orf30, MLF1, ATP8A2, OCRL, PTPN23, JADE1, CEP170, CEP128, ARHGAP35, WDR11, KIF5B, MYO5A, EHD1, CYLD, AKT1, BBS2 ,CFAP20, SEPT2, TRAF3IP1, ATG16L1, NEK4, CENPJ, CETN2* | 102 | 56 | 0.21 | 2.50 | 0.00 | 0.02 |
| GO:0006397 | mRNA processing | *DHX36, DDX41, SUPT6H, DBR1, AKAP8L, RNF40, RNF20, HTATSF1, PSIP1, WBP11, RBM17, SF3A3, PUF60, CMTR1, GPKOW, CASC3, DAZAP1, HABP4, HNRNPH2, USP4, HNRNPA1, SON, NONO, CRNKL1, HNRNPA3, FMR1, GTF2H2, C9orf78, TFIP11, SFPQ, RBM4B, CIRBP, TRA2B, DYRK1A, ARL6IP4, DDX46, LSM2, CPSF4, DHX38, ALKBH5, ZMAT2, CACTIN, TUT1, POLR2H, SNRNP200, MAGOHB, AQR, SRSF10, SETX, SRSF7, PQBP1, ZNF326, PRKACA, RBM26, SRSF2, CDK9, CDC40, ESS2, SYF2, POLR2K, PRPF6, DDX17, RBM4, CCAR1, EFTUD2, SLTM, SRSF6, DHX35, AAR2, HNRNPD, SF3B2, SART3, CHERP, SRSF11, RBMX, NCBP3, PCF11, SUPT5H, SMNDC1, DHX15, HNRNPLL, PRPF38A, CD2BP2, CDK11B, PCBP1, RBM8A, LUC7L3, SNUPN, IWS1, RBM5, FRG1, SNRPE, FASTKD5, RBM42, RBM15B, SNU13, PRPF8, ACIN1, FAM172A, NELFE, RBM15, CCNT1, HNRNPH3, RNF113A, CLP1, GRSF1, DHX16, SRSF3, DDX42, AHCYL1, U2AF1L5, PABPC1, SRSF9, YBX1, SMN1, LUC7L2, TRA2A, SRRM2, SNRPB2, SCAF8, CWC15, ZRANB2, SRSF5, RBM27, CDK12, POLR2B, SNRPD3, CCAR2, CPSF3, SF3B3, ZC3H14, U2SURP, CPSF7, RNPC3, GCFC2, DHX8, PRPF4, RBM25, THRAP3, SRSF1, RNPS1, SF1, CWC22, HNRNPU, JMJD6, PRPF39, HNRNPUL1, U2AF2, EIF4A3, HNRNPH1, SNRPA, ZC3H13, PAN3, SNRNP70, THOC7, METTL3, XRN2, DDX39A, DNAJC8, SCAF1, SNRPF, SMU1, CTR9, CPSF1, CPSF6, KHDRBS3, PNPT1, ADAR, POLR2A, MFAP1, NUDT21, PRPF4B, PRPF38B, SF3B4, USP39, DDX5, SF3B1, DHX40, TENT4B, SUGP1, YTHDC1, HNRNPR, SYNCRIP, DHX9, ERCC2, THOC5, PNN, HSF1, SAP18, SF3A1, RBMXL1, SNRPD2, SARNP, PDE12, PRDX6, MTPAP, CTNNBL1, PDCD7, SRRM1, POLR2E, RBM10, CWC27, ECD, SNRPB, RBBP6, SRRT, RBM23, NCBP2, PAPOLG, PPWD1, IK, RBM3, SART1, POLR2G, PPIL1, PRPF40A, PRPF31, RBM39, DDX23, HNRNPK, RNGTT, PABPN1, RBM19, TSEN15, HNRNPL, CSTF1, ZCRB1, PPP4R2, SUGP2, KHDRBS1, PPP1R8, CCNH, DDX39B, SCAF11, THOC3, PRCC, PRMT5* | 346 | 237 | 0.12 | 2.49 | 0.00 | 0.01 |
| P00047 | PDGF signaling pathway | *PIK3R3, GSK3B, NCK1, MAPKAPK2, BRAF, PIK3R1, ETV3, GSK3A, RPS6KA3, MAP2K2, SOS1, NCK2, ERF, ARAF, PIK3C3, PLCG1, ELP1, RPS6KA1, PRKCA, MAPK3, MAPK1, NIN, ARHGAP12, MAP3K4, MAP3K2, PKN2, USF2, ARHGAP26, STAT1, PLCG2, MAP2K1, RPS6KB2, ARHGAP5, GAB1, PIK3R2, PDPK1, ARHGAP10, GABPA, MYC, RPS6KA5, AKT2, STAT3, RPS6KB1, RPS6KA4* | 52 | 44 | 0.29 | 2.44 | 0.00 | 0.01 |
| hsa04012 | ErbB signaling pathway | *PIK3R3, GSK3B, CAMK2D, NCK1, BRAF, PIK3R1, CBL, MAP2K2, CAMK2G, SOS1, NCK2, ARAF, PLCG1, PRKCA, MAPK3, MAPK1, PAK4, ABL2, ABL1, MTOR, AKT1, PAK2, PAK1, PLCG2, MAP2K1, RPS6KB2, GAB1, SRC, PIK3R2, MYC, AKT2, RPS6KB1, PRKCB, AKT3* | 42 | 34 | 0.31 | 2.40 | 0.00 | 0.01 |
| WP673 | ErbB Signaling Pathway | *PIK3R3, GSK3B, CAMK2D, NCK1, BRAF, PIK3R1, TP53, CBL, MAP2K2, CAMK2G, SOS1, NCK2, ARAF, PLCG1, PRKCA, MAPK3, MAPK1, PAK4, ABL2, ABL1, MTOR, AKT1, PAK2, PAK1, PLCG2, MAP2K1, RPS6KB2, GAB1, SRC, PIK3R2, PDPK1, MYC, AKT2, RPS6KB1, PRKCB, AKT3* | 45 | 36 | 0.30 | 2.39 | 0.00 | 0.02 |
| WP4656 | Joubert Syndrome | *NPHP3, BBS7, ARL13B, CEP41, CEP290, PCM1, RHEB, NIN, MRE11, PARP1, MTOR, MYO6, KAT5, PDE6D, MYO5A, CCP110, BBS2, CETN2, PCNT, FLNA, CEP97, OFD1* | 28 | 22 | 0.37 | 2.35 | 0.00 | 0.02 |
| GO:0042176 | Regulation of protein catabolic process | *SEC22B, NEDD4L, GSK3B, MAD2L1, RNF40, BAG2, DNAJB2, BBS7, CSNK1D, GSK3A, SMARCC1, SDCBP, LATS1, RDX, ITCH, GIPC1, CTSA, MSN, USP25, ARAF, EEF1A2, TLK2, AURKA, HSP90AA1, BAG5, GGA3, ATG4B, SUMO1, FMR, PSMF1, CUL4B, PIAS1, CSNK2A2, PIN1, DERL2, EZR, KEAP1, PLK1, WDR91, MAP1A, FAM83D, SENP1, STUB1, RELA, DDA1, ARIH1, TRIM32, PRKACA, NRDC, PSMD10, PSMD2, NUB1, ROCK1, MYCBP2, PSMD3, USP7, STX5, RPS7, CSNK2A1, NDUFA13, HECW2, AKT1, SNX9, FAM192A, TMF1, IDE, HSPBP1, PTPN3, ALAD, FLNA, HGS, DDRGK1, HSP90AB1, NSF, RYBP, CCAR2, SF3B3, OGT, PSMC4, USP14, PSMC1, PDCL3, WWP2, LRP1, BCAP31, OS9, PML, SNRNP70, UBE3A, VCP, DTL* | 156 | 91 | 0.16 | 2.24 | 0.00 | 0.04 |
| GO:0071103 | DNA conformation change | *DHX36,MCM6 ,ANXA1 ,H1FX,AKAP8L ,PSME4,TOP3A, MCM2, SMC4, OIP5, TP53, MCM4, HMGB3, NCAPD2, H3F3A, HMGB2, NCAPD3, HMGB1, CENPI, NCAPG2, CDKN2A, MIS18A, SMC2, AKAP8, HIST2H2BF, GTF2H2, HIST1H3A, HIST4H4, HIST2H3C, HIST2H2BE, HIRA, RPA1, INO80, MCM7, HJURP, SETX, SET, HIST1H1E, NCAPG, RUVBL2, PHF23, DAXX, TOP2A, CHD1L, SMARCA1, MRE11, H2AFY2, ERCC6, SART3, RPA2, CENPV, NCAPH2, CENPC, KNL1, CENPO, ACIN1, DDX3X, HAT1, RUVBL1, TWNK, WRNIP1, RBBP4, RSF1, CDK1, CDCA5, TOP3B, NUSAP1, POLE3, ASCC3, WAPL, CHD4, ATRX, NCAPH, HELB,TTN, XRCC5, RECQL5, CENPU, KAT6A, BRIP1, PAF1, NBN, CENPH, CHD1, DHX9, ERCC2, CENPM, RBBP7, HIST1H1C, RECQL4, NAA10, NAP1L1, SUPV3L1, BRD2, CHD3, CDC45, RECQL, CTCF, GRWD1, XRCC6, TSPYL1, IPO4, RAD50, UBN1, H2AFY, TOP1, HELLS, ANP32B* | 146 | 108 | 0.16 | 2.22 | 0.00 | 0.04 |
| P00005 | Angiogenesis | *PIK3R3, GSK3B, PRKCI, NCK1, MAPKAPK2, BRAF, PIK3R1, NOTCH2, SPHK2, MAP2K2, SOS1, NCK2, DVL2, PXN, ARAF, PRKCQ, PIK3C3, PLCG1* | 51 | 18 | 0.26 | 2.19 | 0.01 | 0.02 |
| WP4685 | Melanoma | *PIK3R3, BRAF, PIK3R1, TP53, MAP2K2, SOS1, ARAF, CDKN2A, LAMTOR3, MAPK3, MAPK1, CREB1, AKT1, VCL, PAK1, MAP2K1, E2F3, CALM3, PIK3R2, AKT2, CDK4, AKT3* | 26 | 22 | 0.35 | 2.18 | 0.00 | 0.05 |
| hsa03040 | Spliceosome | *WBP11, RBM17, ISY1, SF3A3, PUF60, HNRNPA1, CRNKL1, HNRNPA3, TRA2B, DDX46, LSM2, DHX38, ZMAT2, SNRNP200, MAGOHB, AQR, SRSF10, SRSF7, PQBP1, SRSF2, CDC40, SYF2, PRPF6, EFTUD2, SRSF6, SF3B2, CHERP, RBMX, SMNDC1, DHX15, PRPF38A, PCBP1, RBM8A, SNRPE, SNU13, PRPF8, ACIN1, DHX16, SRSF3, DDX42, TCERG1, U2AF1L5, U2AF1, SRSF9, TRA2A, SNRPB2, CWC15, SRSF5, SNRPD3, SF3B3, U2SURP, DHX8, PRPF4, RBM25, SRSF1, HNRNPU, U2AF2, EIF4A3, SNRPA, SNRNP70, SNRPF, PRPF38B, SF3B4, USP39, DDX5, SF3B1, SF3A1, RBMXL1, SNRPD2, CTNNBL1, SNRPB, NCBP2, SART1, PPIL1, PRPF40A, PRPF31, CCDC12, DDX23, HNRNPK, DDX39B, THOC3* | 111 | 81 | 0.17 | 2.13 | 0.00 | 0.03 |
| GO:0031227 | Intrinsic component of endoplasmic reticulum membrane | *STIM1, PIGT, EIF2AK3, EMC3, EMC7, PIGK, AMFR, PDIA3, HSPA5, EMC1, TBL2, DNAJB12, FKBP8, SEC61A1, AUP1, CANX, ANKLE2, SCD, MMGT1, EMC2, TMCO1, SEC63, RTN4, EMC8, HM13, RRBP1, TMEM33, SGPL1* | 38 | 28 | -0.27 | -2.01 | 0.01 | 0.03 |
| GO:0017056 | Structural constituent of nuclear pore | *NUP98, NUP155, POM121, NUP35, NUP160, NUP88, NUP107, NUP188, NUP153, NUP133, NUP214, TPR, UPF3B, NUP93, NUP205, NUP85* | 17 | 16 | -0.45 | -2.22 | 0.00 | 0.04 |
| hsa05010 | Alzheimer disease | *ATP2A2, ITPR3, EIF2AK3, APBB1, SDHA, UQCRQ, NDUFS6, CASP7, ATP5F1C, BAD, UQCRC2, CYC1, NDUFA4, UQCRFS1, MAPT, ITPR2, NDUFA11, NDUFA6, CAPN1, SDHB, ATP5PD, NDUFA9, NAE1, ATP5PB, PLCB1, NDUFA10, UQCRC1, NDUFS1, COX4I1, NCSTN, NDUFB10, NDUFS7, GAPDH* | 58 | 33 | -0.25 | -2.24 | 0.00 | 0.02 |
| GO:0006605 | protein targeting | *HACL1, PMPCB, RPLP2, TIMM21, ERBIN, RPL17, BAG3, RPL14, MIPEP, RPLP1, TIMM44, RPL10, RPS4X, VPS13A, YWHAG, PDCD5, UQCRC2, CAT, RPS17, TIMM50, RPL37A, RPL23, CDC37, USP36, PRKAA1, RPS14, RPL13A, RPS8, RPS18, RPL22, EXOC4, RPS23, RPL9, RPS11, RPL38, AIP, TOMM22, SAMM50, TYSND1, AGPS, RPS3A, AKAP12, SSR1, PEX14, RPS27, AP3M1, RPS2, RAB3IP, TIMM23, RPS13, RPL31, RPL11, RPL13, RPL4, ARIH2, RPL18, RPS6, RPS9, LARS, HSPA8, RPS5, RPS12, RPL27A, RPL8, SEC61A1, ECI2, LONP2, RPL24, RPS26, RPSA, RPL34, RPL29, RPS24, RPL27, RPL6, STOM, RPS27, RPS29, RPL15, RPS10, RPS20, AP3B1, ECH1, SLC25A6, HSPA4, KIF13B, C2CD5, TCAF1, RPL28, NUDT19, SRP72, RPL7, RPS16, RPS3, SEC63, RPL7A, RPL23A, ZFYVE16, RPL19, NPEPPS, SSR3, RPL30, RPS15A, RPL18A, SCP2, ATG13, RPLP0* | 188 | 106 | -0.14 | -2.24 | 0.00 | 0.03 |
| WP4297 | Thiamine metabolic pathways | *BCKDHA, SLC25A19, TKT, BCKDK, PDHA1,OGDH* | 6 | 6 | -0.74 | -2.27 | 0.00 | 0.04 |
| GO:0070003 | threonine-type peptidase activity | *PSMA5, PSMA3, PSMB7, PSMB1, PSMB4, PSMA6, PSMA2, PSMB2, PSMB3, PSMA1, PSMB5, PSMA4* | 14 | 12 | -0.49 | -2.29 | 0.00 | 0.03 |
| GO:0005759 | Mitochondrial matrix | *MRPS6, RPUSD3, MRPS27, MRPS28, PMPCB, TUFM, MRPS34, MRPS18B, BCKDHB, PUS1, FARS2, TFB1M, MIPEP, TIMM44, ECHS1, MRPS10, QARS, MAIP1, MRPS21, BCKDHA, DDX28, ATP5F1C, MCCC1, GLUD1, EARS2, CCNB1, PDK1, MRPS12, DHTKD1, NADK2, MRPS15, ALDH1B1, OAT, HSD17B8, MRPS16, MRPL14, ACADSB, NR3C1, NARS2, PDK2, OXCT1, MRPS26, PARK7, VDAC1, HSPA9, MRPS7, PARS2, MRPS22, LONP1, DIMT1, MRPL13, ALDH7A1, DBT, MRPL17, DNAJA3, MTHFD1L, NDUFA9, MCAT, MRPL20, IVD, MRPS35, MRPS2, MRPL23, ATP5PB, MRPL48, TRAP1, LRRC59, BCKDK, DLD, FASTKD2, MRPL39, TRUB2, ETFA, TFB2M, MRPL37, MRPS9, SHC, DARS2, NDUFA10, PDHA1, POLG2, PDHB, COQ5, OXA1L, OGDH, MRPS5, ABCE1, NDUFS1, SUCLA2, MRPL44, DLAT, TBRG4, MRPS30, CS, NDUFS7, PCK2, MRPL11, NUBPL, ACSF3, HADHA, PDSS2, GFM2, SHMT2, GOT2, MMAB, RNASEL, DAP3, CLPX, SUCLG1, FECH, SLC25A5, RPS3, SCO2, GLRX5, MRPL42, YARS2, C1QBP, MRPS17, MPG, DHX30, ALDH5A1, GLS, MCCC2, HADHB, GSR, ETFB, IDH3B, MRPL22, GCDH, RPL55, TARS2, NDUFS3, LRPPRC, AASS, MLYCD, MRPL15, PDE12, AK4, IDH3A, ACADM, PRDX3, SUPV3L1, TMLHE, TFAM, MRPL46, ACO2, TDRD7, MRPS31, HSPD1, ALDH1L2, PCCA, TSFM, MRPL41, SUCLG2, VDAC2, ATP5F1A, COASY, MRPL24, DLST, PMPCA, ERAL1* | 228 | 161 | -0.14 | -2.34 | 0.00 | 0.00 |
| hsa03022 | Basal transcription factors | *TAF9, TAF6L, TAF6, TAF5L, GTF2I, TAF7, GTF2H3, GTF2H1, CDK7, MNAT1, ERCC3, TAF9B, GTF2H4, GTF2F1, TAF1, TAF8, GTF2F2, TAF5* | 27 | 18 | -0.39 | -2.39 | 0.00 | 0.01 |
| GO:0101031 | Chaperone complex | *CCT5, CCT3, TCP1, CCT4, CCT8, CCT7, BAG3, CCT2, CCT6A, CDC37* | 17 | 10 | -0.48 | -2.41 | 0.00 | 0.00 |
| hsa03013 | RNA transport | *EIF3F, EIF3C, NUP210, NUP43, RANBP2, EIF3H ,THOC1, NUP98, NUP155, EIF3G, POM121, PHAX, NUPL2, RPP14, NUP35, THOC6, EIF3I, EIF3E, RPP30, EIF3B, GEMIN4, RPP40, CYFIP2, AAAS, NUP160, RPP25, DDX20, XPOT, PAIP1, POP5, EIF3D, SEH1L, NUP88, POM121C, EIF4G3, NUP37, EIF3A, EIF4G2, RPP38, RPP25L, KPNB1, EIF2B4, TGS1, GEMIN5, NUP107, NUP188, NUP153, RAE1, NCBP1, EIF4A2, POP1, EIF2S3, STRAP, THOC2 , POP4, NUP133, CLNS1A, NUP214, PABPC4, TPR, UPF3B, RGPD8, NUP50, EIF4B, RANGAP1, NUP93, EIF2B3 , NXF1, EIF1AX, EIF4A1, EIF3J, EIF2S1, THOC5, SAP18, XPO5, EIF5B, SENP2, NCBP2, NUP205, XPO1, RAN, DDX39B, NUP85, THOC3, PRMT5* | 125 | 86 | -0.19 | -2.41 | 0.00 | 0.01 |
| GO:0019843 | rRNA binding | *MRPS6, MRPS27, NGRN, RPS4X, DDX28, EEF2, RPL23, RPS14, RPS18, RPL9, RPS11, MRPS7, MRPL20, RPS13, RPL11, PTCD3, FASTKD2, DDX21, RPS9, SBDS, NOP53, RPS5, RPL8, UTP25, MRPL11, PPAN, RNASEL, RPS3, RPL23A, RPL19, MRPS17, KDM2B, RPLP0, IMP4, RPL5, RCC1L, ERAL1* | 46 | 37 | -0.30 | -2.46 | 0.00 | 0.01 |
| C0030567 | Parkinson Disease | *RPL14, RPS8, TALDO1, PARK7, MAPT, HSPA9,VPS35, RPL6, DDIT4, IGF2R, RPL23A,GAK* | 12 | 12 | -0.58 | -2.48 | 0.00 | 0.05 |
| hsa04714 | Thermogenesis | *SIRT6, SDHA, SMARCD2, KDM3B, ZNF516, UQCRQ, PRKAG1, NDUFS6, AKT1S1, ATP5F1C, ATP5ME, MAP2K3, UQCRC2, CYC1, ACTL6A, PRKAB2, KDM1A, PRKAA1, NDUFA4, DPF3, UQCRFS1, SMARCA2, PRKAG2, NDUFA11, ATF2, NDUFA6, COX15, SDHB, ATP5PD, NDUFA9, PRKAA2, ATP5PB, CPT1A, RPS6, NDUFA10, SMARCC2, UQCRC1, NDUFS1, COX4I1, NDUFAF4, GNAS, NDUFB10, NDUFS7, PRKACB* | 81 | 44 | -0.23 | -2.49 | 0.00 | 0.00 |
| GO:0034470 | ncRNA processing | *LAGE3, PDCD11, DUS2, NAT10, REXO4, TOE1, NSUN5, DUS3L, PELP1, NPM3, THUMPD3, UTP14A, RPL7L1, TSEN54, MTREX, PUS1, FARS2, RPL14, TFB1M, RPP14, DDX52, RPP30, DDX56, DDX47, GEMIN4, RPP40, GTPBP3, TRMT112, TARBP1, UTP4, RPS17, UTP11, SMAD3, MTO1, RPP25, USP36, NOP14, POP5, RPS14, RPS8, URB1, ELP4, NHP2, PUS7, CDK5RAP1, LYAR, TSEN34, MOCS3, DIMT1, TRMU, RPS27, SRFBP1, TYW1, RPP38, RPS2, NSUN2, RSL1D1, AGO3, RPL11, DDX49, BUD23, KRI1, NOB1, DHX37, FTSJ3, DDX21, RPS6, RPS9, INTS10, SBDS, RTRAF, PA2G4, TFB2M, NOP53, EXOSC5, MRPS9, NCBP1, TP53RK, DICER1, NGDN, WDR46, DDX1, POP1, THG1L, RCL1, TARBP2, WDR55, WDR4, FRG1, DROSHA, NOL6, RRP8, RRP9, RPSA, ESF1, CTU2, EXOSC6, SNU13, WDR36, INTS4, UTP25, NOP56, MRPL44, POP4, NOL8, RPS24, SENP3, RPL27, NOLC1, EXOSC4, NOL10, RNF113A, CLP1, RPS27, MRM3, BYSL, TSR3, GRSF1, KARS, INTS14, RRP1B, RPS15, SARS, ZNHIT6, METTL1, SHQ1, RRP7A, TRMT11, ELP2, EXOSC7, CDKAL1, QTRT2, CHD7, INTS11, RNASEL, NAF1, FTSJ1, ELP3, DIS3L2, NOC4L, NOP58, NOP2, MRTO4, GAR1, METTL16, CPSF3, TRMT1L, RPL7, RPS16, HSD17B10, RIOK2, WDR43, MRPL1, TRMT10C, RPUSD2, USP14, EXOSC2, RTCB, ABT1, RPL7A, WDR18, IMP3, HNRNPA2B1, RPS19, DCAF13, DDX27, TRMT6, EXOSC1, EIF4A3, DKC1, WDR12, AARS2, RIOK1, TEX10, METTL3, DDX51, XRN2, WDR75, UTP3, BOP1, TRPT1, UTP18, ADAR, FBL, TENT4B, BMS1, UTP6, HEATR1, EXOSC3, RRP15, PES1, INTS9, WDR3, TRMT1, RPL10A, SRRT, SUV39H1, RPS21, INTS7, RPL26, SSB, SART1, TUT4, NOL11, GTPBP4, OSGEP, TSR1, INTS1, THUMPD1, IMP4, MDN1, UTP15, TSEN15, RPL5, RIOK3, TUT7, MPHOSPH10, INTS3* | 258 | 217 | -0.14 | -2.57 | 0.00 | 0.00 |
| GO:0006091 | Generation of precursor metabolites and energy | *MTFR1L, PMPCB, PFKP, SIRT6, GLRX3, ZBTB7A, SDHA, GFPT1, SLC25A22, UQCRQ, PRKAG1, NDUFS6, NIPSNAP2, GFPT2, ATP5F1C, LDHA, SLC25A13, ATP5ME, CCNB1, UQCRC2, ARNT, DHTKD1, CYC1, CAT, ALDH1B1, PFKL, PRKAA1, NDUFA4, EIF6, OXCT1, UQCRFS1, PARK7, PRKAG2, NOA1, NDUFA6, PKM, SCO1, MYBBP1A, COX15, SDHB, AKR7A2, ATP5PD, PASK, NDUFA9, PRKAA2, ATP5PB, TRAP1, POR, DLD, FASTKD2, ASPH, ETFA, NOP53, NDUFA10, SLC25A12, IRS2, PDHA1, PHKA2, POLG2, PDHB, PPP1CC, UQCRC1, GPD2, PPP1R3D, OXA1L, OGDH, NDUFS1, SUCLA2, COX4I1, PPP1CA, ENO1, GNAS, HK2, NDUFB10, DLAT, ADSL, TBRG4, CS, NDUFS7, DDIT4, STOML2, PHKB* | 166 | 82 | -0.19 | -2.87 | 0.00 | 0.00 |
| R-HSA-8953854 | Metabolism of RNA | *LAGE3, PDCD11, DUS2, NAT10, HNRNPF, NUP210, RPLP2, NUP43, RANBP2, PELP1, THOC1, UTP14A, TSEN54, MTREX, PUS1, NUP98, BCAS2, RPL17, RPL14, NUP155, TFB1M, RPLP1, ELAVL1, POM121, GNL3, PHAX, NUPL2, RPP14, NUP35, RPL10, DDX52, THOC6, RPS4X, ADARB1, RNMT, RPP30, DDX47, GEMIN4, RPP40, GTPBP3, AAAS, TRMT112, PSMA5, UTP4, GTF2H3, WDR61, RPS17, CNOT1, NUP160, SF3B5, RPL37A, UTP11, RPL23, PRKCD, MTO1, RPP25, DDX20, XPOT, PAIP1, NOP14, POP5, RPS14, SNRPA1, IGF2BP3, RPL13A, RPS8, SEH1L, RPS18, CPSF2, RPL22, NHP2, PPP2CA, NUP88, PSMA3, RPS23, PUS7, RPL9, KHSRP, HNRNPM, RPS11, RPL38, PSMB7, PSMD13, TSEN34, NUP37, PCBP2, DIMT1, GTF2H1, CDK7, PSMB1, RPS3A, MNAT1, U2AF1L4, PSMC3, TRMU, PSMB4, CSTF3, RPS27, RPS27L, PSME1, TYW1, RPP38, RPS2, SNRPG, RPS13, CNOT10, NSUN2, RPL31, RPL11, DDX49, RPL13, BUD23, PSME2, SLU7, HNRNPA0, PLRG1, HSPA1B, TGS1, ERCC3, GEMIN5, NOB1, SF3A2, PSMD9, RPL4, NUP107, DHX37, GTF2H4, RPL18, FTSJ3, DDX21, WDR33, PSMA6, GTF2F1, NUP188, NUP153, RPS6, RPS9, POLDIP3, RTRAF, HSPA8, EXOSC5, RPS5,RAE1, NCBP1, TP53RK, RPS12, PAN2, RPL27A, BUD31, APOBEC3C, SNRNP40, EIF4A2, WDR46, DDX1, PPIE, POP1, RBM28, ZC3H11A, THG1L, PSMD12, RCL1, PSMA2, RPL8, CDC5L, GTF2F2, WDR4, RPL24, RPS26, THOC2, NOL6, RPSA, SLBP, RPL34, RPL29, LTV1, UTP25, TTC37, POP4, NUP133, PSME3, CLNS1A, PSMB2, RPS24, CNOT8, RPL27, NUP214, POLR2C, RPL6, PRPF3, WTAP, RPS27, BYSL, RPS29, GLE1, SNW1, RPL15, RPS10, TPR, RPS20, PSMD11, RRP7A, ETF1, TRMT11, PATL1, SF3B6, PRPF1, UPF3B, PSMC2, CDKAL1, PPP2R1A, PSMB3, RPL28, PSMA1, XAB2, RBM22, PSMB5, PSMA4, SRSF4, PSMD14, NOC4L, RPS27A, NUP50, NOP58, NOP2, GAR1, CPSF3, SF3B3, RPL7, RPS16, HSD17B10, U2SURP, RPS3, RIOK2, WDR43, EIF4B, TRMT10C, EDC3, HNRNPC, CPSF7, RNPC3, GCFC2, SNRPD1, PSMC4, PRPF4, EXOSC2, PSMC1, SMG7, RTCB, RPL7A, PWP2, WDR18, RPL23A, SRSF1, PNO1, RNPS1, PAPOLA, SF1, IMP3, PPIH, HNRNPA2B1, PSMD1, FIP1L1, CWC22, HNRNPU, HSPB1, RPS19, PTBP1, RPL19, DCAF13, NUP93, PSMD6, RPL12, TRMT6, HNRNPUL1, EXOSC1, U2AF2, EIF4A3, HNRNPH1, NXF1, DKC1, WDR12, SNRPA, RIOK1, PAN3, SNRNP70, RPL30, TEX10, GSPT1, THOC7, CSNK1E, METTL14, METTL3, XRN2, DDX39A, DDX6, DNAJC8, EIF4A1, FUS, RPS15A, SNRPF, WDR75, UTP3, RPL18A, BOP1, CPSF1, PSMC6, RPLP0, NIP7, UTP18, ADAR, POLR2A, PSMD4, NUDT21, SF3B4, USP39, RPL3, DDX5, FBL, SF3B1, SUGP1, BMS1, PSMA7, HNRNPR, UTP6, DHX9, HEATR1, ERCC2, THOC5, SF3A1, SNRPD2, SARNP, EXOSC3, PES1, CTNNBL1, PDCD7, SRRM1, POLR2E, WDR3, TRMT1, CWC27, RPL10A, SNRPB, FYTTD1, SRRT, NCBP2, RPS21, RPL26, PPWD1, EPRS, SART1, POLR2G, DCP2, PPIL1, TUT4, PRPF40A, PRPF31, RPL21, PSMB6, NOL11, PSMD7, DDX23, OSGEP, SMG5, TSR1, HNRNPK, PPIL4, THUMPD1, RNGTT, IMP4, CNOT4, PABPN1, NUP205, XPO1, UTP15, TSEN15, HNRNPL, CSTF1, ZCRB1, PSMC5, RPL5, RIOK3, RAN, TUT7, MPHOSPH10, CCNH, DDX39B, SMG1, NUP85, THOC3, PRCC, PRMT5* | 536 | 390 | -0.12 | -2.90 | 0.00 | 0.00 |
| PA447172 | Mitochondrial Diseases | *TACO1, CKMT1B, CKMT1A, OPA1, PMPCB, TUFM, SDHA, PUS1, FARS2, CLPB, SLC25A22, ECHS1, SLC25A15, SLC25A13, GTPBP3, MRPS12, NADK2, MRPS15, MICOS13, MTO1, SFXN4, PRKAA1, NDUFA4, SLC25A19, MRPS16, NARS2, PARK7, VDAC1, HSPA9, NDUFA11, SLC25A1, NDUFA6, PARS2, MRPS22, SCO1, LONP1, AIFM1, MRPL13, CAPN1, COX15, MRPL17, SDHB, TRMU, NDUFA9, MRPL20, MRPS2, MRPL23, ATP5PB, TRAP1, FASTKD2, MRPL39, BCS1L, MSTO1, ETFA, KIF21A, LARS, MRPL37, DARS2, NDUFA10, SLC25A12, PDHA1, POLG2, PDHB, AFG3L2, COQ5, MRPS5, NDUFS1, SUCLA2, PHB, COX4I1, TMEM70, DUFAF4, SLC25A24, CS, NDUFS7, MTCH2, NUBPL, HADHA, GFM2, SLC25A6, NDUFS4, LETM1, SUCLG1, PANK2, SLC25A5, SCO2, GLRX5, YARS2, C1QBP* | 153 | 89 | -0.20 | -2.98 | 0.00 | 0.00 |
| R-HSA-1430728 | Metabolism | *CNDP2, TACO1, CKMT1B, CKMT1A, ACACA, HACL1, NUP210, RPLP2, ITPR3, TBL1XR1, NUP43, SIN3A, NT5C2, RANBP2, NAMPT, IMPDH1, PFKP, GNB1, BCKDHB, DCTD, EEFSEC, SDHA, FAR1, NUP98, RPL17, SMPD1, AIMP2, RPL14, PIK3C2A, NUP155, RPLP1, POM121, NUPL2, ABCC1, NUP35, RPL10, ECHS1, UQCRQ, NDUFS6, RPS4X, MTMR12, QARS, BCKDHA, HSD17B12, ATP5F1C, SLC25A15, LDHA, MCCC1, GLUD1, MARS, SLC25A13, PPP2R1B, PIP5K1C, ESRRA, AAAS, ATP5ME, TRMT112, SMPD4, UQCRC2, GMPR2, PDK1, HACD3, PSMA5, ARNT, DHTKD1, CYC1, NADK2, RPS17, SIN3B, NUP160, ALDH1B1, PRKAB2, GART, RPL37A, SMS, OAT, RPL23, HSD17B8, GPHN, PFKL, NDUFA4, GLB1, SYNJ1, SLC25A19, IMPDH2, PFAS, RPS14, RARS, ACADSB, RPL13A, RPS8, SEH1L, TALDO1, RPS18, NCOR2, PDK2, RPL22, OXCT1, UQCRFS1, CRYL1, PPP2CA, NUP88, VDAC1, PSMA3, AGRN, LPCAT1, PRKAG2, MTHFD1, ITPR2, RPS23, RPL9, NDUFA11, RPS11, RPL38, SLC25A1, AIP, PSMB7, SBF2, NDUFA6, PARP4, PSMD13, RRM1, NUP37, PKM, SCO1, MOCS3, ASNS, PSMB1, AGPS, RPS3A, ALDH7A1, CARM1, DBT, COX15, SDHB, MTHFD1L, AKR7A2, ATP5PD, PSMC3, NDUFA9, PSMB4, SCLY, RPS27, RPS27L, MCAT, PSME1, CYB5R3, PRKAA2, OSBP, MTAP, IVD, RPS2, ATP5PB, TKT, RPS13, CPT1A, TRAP1, PNPLA6, KPNB1, PLCB1, RPL31, RPL11, RPL13, BCKDK, PSME2, POR, DLD, TGS1, PPT1, PSMD9, LPIN3, RPL4, NUP107, RPL18, MARCKS, ETFA, PSMA6, NUP188, NUP153, RPS6, RPS9, MED17, PIK3C2B, LARS, NCOA3, RPS5, RAE1, NDUFA10, RPS12, SLC25A12, ACOT7, RPL27A, PDHA1, PHKA2, PDHB, PPP1CC, PSMD12, OSBPL3, UQCRC1, GPD2, AIMP1, COQ5, OGDH, PSMA2, RPL8, CYP51A1, ECI2, PLEKHA3, EEF1E1, PLEKHA5, PDZD11, NDUFS1, SUCLA2, RPL24, RPS26, COX4I1, PPP1CA, RPSA, OSBPL5, SCD, RPL34, SYNJ2, RPL29, NDUFAF4, ENO1, DARS, GNAS, DNM2, NUP133, PSME3, PSMB2, HK2, NDUFB10, RPS24, DLAT, RPL27, ADSL, NUP214, AK6, RPL6, CS, MMS19, NDUFS7, RPS27, PCK2, PHKB, RPS29, NUBPL, ACSF3, PRKACB, GGPS1, HADHA, RPL15, RPS10, PDSS2, TPR, GAPDH, NME1, PFKFB3, GCLC, RPS20, PSMD11, HMMR, SPTLC1, GUSB, PISD, MAN2B2, HK1, SLC25A6, NDUFS4, PSMC2, NUDT15, SHMT2, PPP2R1A, GOT2, PSMB3, MMAB, PIAS4, RPL28, PSMA1, CBSL, NUDT19, PPP2R5D, PSMB5, PSMA4, SMARCD3, SUCLG1, FECH, PSMD14, NUP50, PANK2, RPL7, RPS16, SLC25A5, RPS3, PIP4K2C, SCO2, GLRX5, RPL7A, RPL23A, CIAO1, PSMD1, IARS, RPL19, SLC3A2, NUP93, CDK8, CTH, MED4, ALDH1L1, CSNK2B, ASS1, GLS, RPL30, SEC24D, RPS15A, PRKD3, PRKAR2B, RPL18A, RDH11, PAPSS2, HADHB, UQCR10, PHGDH, PSMC6, SCP2, GPX4, RPLP0, MAT2B, ABCC5, PSMD4, ABCB7, SP1, GCDH, PRKD1, PPAT, LRPPRC, NUDT5, MLYCD, AK4, PRKAR2A, IDH3A, ECSIT, MTM1, ACADM, ATP5MF, RPL10A, RUFY1, PPIP5K2, TMLHE, RPL26, ACO2, NAGLU, EP300, OSBPL8, SGPL1, EPRS, PCCA, PGP, RPL21, PPA1, PSMB6, LDHB, PSMD7, ATP5MG, LCLAT1, NUP205, ACSL3, SGSH, PECR, CKB, INPP5F, PSMC5, RPL5, ATP5F1A, COASY, NCOR1, PANK4, NME2* | 642 | 378 | -0.11 | -3.03 | 0.00 | 0.00 |
| C1836440 | Increased serum lactate | *TACO1, SDHA, FARS2, ECHS1, UQCRQ, LDHA, UQCRC2, CYC1, MTO1, MRPS16, SCO1, AIFM1, COX15, TRMU, NDUFA9, FASTKD2, BCS1L, NDUFA10, PDHA1, POLG2, OGDH, TMEM70, NDUFS4, SLC25A3, SCO2, YARS2* | 31 | 26 | -0.46 | -3.08 | 0.00 | 0.00 |
| R-HSA-1643685 | Disease | *VPS4B, ACACA, NUP210, TAF9, ELOC, RPLP2, TBL1XR1, NUP43, SIN3A, RANBP2, AP2A2, CUL1, GFPT1, NUP98, ATG7, RPL17, RPL14, TAF6, ARRB2, NUP155, NPM1, RPLP1, POM121, NUPL2, RASA3, NUP35, MAP2K7, RPL10, CTDP1, RPS4X, EPS15, CTNNB1, CBLL1, CUX1, AKT1S1, HMGA1, RNMT, MCCC1, SLC4A4, PDCD6IP, SLC12A6, PPP2R5E, PPP2R1B, EEF2, BAD, AAAS, MAP2K3, NFKB2, PSMA5, TAF7, GTF2H3, CHMP2A, RPS17, NUP160, RPL37A, CTBP2, AKAP9, SLC1A3, RPL23, SMAD3, KPNA3, CDC37, GLB1, FOXO4, LMNB1, RPS14, RPL13A, RPS8, SEH1L, TALDO1, RPS18, NCOR2, RPL22, PACS1, PPP2CA, NUP88, PSMA3, AGRN, RPS23, BANF1, CAST, RPL9, RPS11, MSH3, RPL38, PSMB7, PSMD13, NUP37, GTF2H1, CDK7, CHMP7, CAPN1, PSMB1, RPS3A, RHOG, MNAT1, PSMC3, AP2M1, PSMB4, RPS27, RPS27L, PSME1, RPS2, RPS13, SKP1, KPNB1, NMT1, RPL31, RPL11, RPL13, PSME2, HSPA1B, ERCC3, LIG4, PSMD9, RBX1, TAF9B, RPL4, KAT2A, NUP107, GTF2H4, ZMYM2, RPL18, CSK, B3GLCT, PSMA6, GTF2F1, NUP188, NUP153, ELOB, RPS6, RPS9, RPS5, SHC1, RAE1, NCBP1, RPS12, SLC27A4, IRS2, RPL27A, RAF1, TAF1, PSMD12, PSMA2, RPL8, GTF2F2, CANX, PHB, RPL24, RPS26, TAF5, KPNA2, RPSA, RPL34, NCSTN, RPL29, IPO5, NUP133, PSME3, PSMB2, RPS24, RPL27, CCDC59, NUP214, POLR2C, RPL6, RICTOR, RPS27, AP1G1, GOLGA2, RPS29, SNW1, RPL15, RPS10, SUPT4H1, TPR, GCLC, RPS20, TSG101, PSMD11, AP3B1, GUSB, ATP6V1H, HK1, SLC25A6, PSMC2, TAF2, PPP2R1A, PSMB3, MMAB, AP1M1, RPL28, PSMA1, LMNA, PPP2R5D, PSMB5, PSMA4, PSMD14, NUP50, RPL7, RPS16, SLC25A5, RPS3, FOXO1, PPIA, NELFB, ZC3HAV1, DAB2IP, NFKB1, RPL7A, RPL23A, KPNA5, PSMD1, RANGAP1, RPL19, JUN, NUP93, CDK8, NF1, RPL30, CHMP5, CAPNS1, RPS15A, SSRP1, RPL18A, MCCC2, FEN1, PAPSS2, VCP, AP2B1, TLN1, RASAL2, PSMC6, CPSF6, SRC, RPLP0, CUL5, POLR2A, CDC25A, PSMD4, NMT2, RAP1B, PIK3R2, RPL3, PDPK1, VPS4A, SUPT16H, PSMA7, XRCC4, ERCC2, ELL, MAPKAP1, APC, RANBP1, AP1B1, POLR2E, CDC25C, RPL10A, MYC, BCR, CCNT2, SEL1L, HDAC3, NCBP2, RPS21, RPL26, NAGLU, BRAP, EP300, AGK, DERL1, POLR2G, GALK1, CCNK, ELOA, XRCC6, PCCA, RPL21, PSMB6, CLTC, PSMD7, ERLIN2, AKT2, STAT3, RNGTT, PABPN1, NUP205, XPO1, SGSH, SND1, PSMC5, RPL5, NCOR1, RAN, ARRB1, CHMP4B, CCNH, PRDX2, NUP85, AKT3, DNAJC3* | 431 | 300 | -0.13 | -3.10 | 0.00 | 0.00 |
| GO:0022613 | Ribonucleoprotein complex biogenesis | *PDCD11, NAT10, EIF3F, REXO4, EIF3C, EIF3K, TAF9, NSUN5, EIF3M, EIF3H, PELP1, NPM3, SURF6, UTP14A, RPL7L1, EIF3L, MTREX, GTPBP10, RPL14, NPM1, BCCIP, TFB1M, EIF3G, RPL10, DDX52, EIF3I, EIF3E, TICRR, DDX28, DDX56, DDX47, EIF3B, SFSWAP, GEMIN4, TRMT112, UTP4, RPS17, CNOT1, UTP11, DDX20, USP36, PRKAA1, NOP14, POP5, EIF3D, RPS14, EIF6, RPL13A, RPS8, URB1, NHP2, NUP88, MAPT, RPS23, GNL2, RPL38, LYAR, SDAD1, MRPS7, DIMT1, MYBBP1A, EIF3A, NOC2L, EFL1, RPS27, RPS27L, SRFBP1, PRKAA2, MRPL20, RPS2, SNRPG, MRPS2, RSL1D1, AGO3, RPL11, DDX49, BUD23, SLU7, KRI1, FASTKD2, TGS1, GEMIN5, NOB1, SF3A2, DHX37, LIMD1, GNL3L, FTSJ3, DDX21, RPS6, RPS9, SBDS, PA2G4, TFB2M, NOP53, EXOSC5, RPS5, MRPS9, NCBP1, DICER1, PAN2, EIF4H, NGDN, WDR46, DDX1, AATF, RCL1, EIF2S3, TARBP2, ABCE1, WDR55, CD2BP2, PWP1, STRAP, RSL24D1, LUC7L3, RPL24, SNUPN, LUC7L, RBM5, FRG1, DROSHA, NOL6, SNRPE, RRP8, CEBPZ, RRP9, RPSA, ESF1, EXOSC6, NUFIP1, SNU13, WDR36, LTV1, UTP25, NOP56, POP4, NOL8, PRPF8, CLNS1A, NMD3, RPS24, DDX3X, SENP3, RPL27, NOLC1, RUVBL1, EXOSC4, OGFOD1, RPL6, NOL10, PRPF3, DENR, CLP1, RPS27, MRM3, BYSL, MRPL11, TSR3, RPS10, RRP1B, RPS15, ZNHIT6, SHQ1, SRPK2, RIOX2, ZNF622, G3BP1, RRP7A, SRSF9, PATL1, URB2, SMN1, LUC7L2, PRPF19, PPAN, EXOSC7, SRSF5, CHD7, HEATR3, RNASEL, NAF1, SRPK1, CDC73, SNRPD3, XAB2, RBM22, HSP90AB1, DIS3L2, NOC4L, NOP58, NOP2, MRTO4, GAR1, METTL16, RPL7, RPS16, RIOK2, WDR43, MRPL1, EIF4B, RPUSD2, EDC3, CPSF7, GCFC2, SNRPD1, EXOSC2, ABT1, RPL7A, WDR18, RPL23A, C1QBP, SRSF1, SF1, IMP3, DYNC1H1, RPS19, DCAF13, RPL12, DDX27, PRPF39, DHX30, EXOSC1, EIF4A3, DKC1, EIF5, WDR12, RIOK1, PAN3, TEX10, DDX51, XRN2, DDX6, EIF3J, SNRPF, WDR75, UTP3, AAMP, BOP1, CPSF6, RPLP0, NIP7, UTP18, ADAR, MRPL22, EIF2S1, BRIX1, NUDT21, USP39, RPL3, FBL, SF3B1, ZNF593, NOM1, TENT4B, BMS1, YTHDC1, UTP6, DHX9, HEATR1, BICD1, SF3A1, SNRPD2, PTGES3, EXOSC3, RRP15, PES1, WDR3, RPL10A, SNRPB, SUV39H1, RPS21, RPL26, SART1, PRPF31, PUM2, NOL11, GTPBP4, DDX23, TSR1, THUMPD1, IMP4, MDN1, XPO1, UTP15, RPL5, RIOK3, RAN, MPHOSPH10, DDX39B, SCAF11, ERAL1, PRMT5* | 341 | 292 | -0.15 | -3.11 | 0.00 | 0.00 |
| R-HSA-390450 | Folding of actin by CCT/TriC | *CCT5, CCT3, TCP1, CCT4, CCT8, CCT7, CCT2, CCT6A* | 8 | 8 | -0.92 | -3.17 | 0.00 | 0.00 |
| GO:0044455 | Mitochondrial membrane part | *OPA1, PMPCB, TAMM41, TIMM21, SDHA, UQCRQ, NDUFS6, ATP5F1C, ATP5ME, UQCRC2, CYC1, MICU1, MICOS13, TIMM50, NDUFA4, SLC25A19, UQCRFS1, PARK7, NOA1, RHOT1, NDUFA11, NDUFA6, TOMM22, SAMM50, SCO1, ATP5MD, COX15, SDHB, ATP5PD, LYN, NDUFA9, ATP5PB, TIMM23, CPT1A, BCS1L, NDUFA10, MICU2, AFG3L2, UQCRC1, COQ5, OXA1L, NDUFS1, PHB, COX4I1, TMEM70, ANKZF1, SYNJ2, NDUFB10, ARMCX3, NDUFS7, PISD, SLC25A6, NDUFS4* | 82 | 53 | -0.31 | -3.28 | 0.00 | 0.00 |
| WP477 | Cytoplasmic Ribosomal Proteins | *RPLP2, RPL17, RPL14, RPLP1, RPL10, RPS4X, RPS17, RPL37A, RPL23, RPS14, RPL13A1, RPS8, RPS18, RPL22, RPS23, RPL9, RPS11, RPL38, RPS3A, RPS27, RPS2, RPS13, RPL31, RPL11, RPL13, RPL4, RPL18, RPS6, RPS9, RPS5, RPS12, RPL27A, RPL8, RPL24, RPS26, RPSA, RPL34, RPL29, RPS24, RPL27, RPL6, RPS27, RPS29, RPL15, RPS10, RPS20, RPL28, RPL7, RPS16, RPS3, RPL7A, RPL23A, RPL19, RPL30, RPS15A, RPL18A, RPLP0* | 74 | 56 | -0.33 | -3.42 | 0.00 | 0.00 |
| GO:0005840 | Ribosome | *MRPS6, MRPS27, MRPS28, RPLP2, EIF2AK3, MRPS34, EIF3H, MRPS18B, SURF6, MRPS25, RPL7L1, RPL17, RPL14, RPLP1, RPL10, RPS4X, MRPS10, MRPS21, EEF2, MRPS12, MRPS15, RPS17, HSPA14, RPL37A, RPL23, MRPS23, RPS14, MRPS16, MRPL14, RPL13A, RPS8, RPS18, RPL22, MRPS26, RPS23, RPL9, RPS11, RPL38, METTL17, MRPS7, MRPS22, MRPL13, RPS3A, MRPL17, RPS27, RPS27L, MRPL20, MRPS35, RPS2, MRPS2, MRPL23, RPS13, MRPL48, RPL31, RPL11, RPL13, PTCD3, MRPL39, GEMIN5, RPL4, RPL18, RPS6, RPS9, RPS5, MRPL37, MRPS9, RPS12, RPL27A, MRPS5, RPL8, CANX, RSL24D1, RPL24, RPS26, RPSA, RPL34, NUFIP1, RPL29, MRPL44, RPS24, RPL27, RPL6, MRPS30, RPS27, RPS29, MRPL11, RPL15, RPS10, APEX1, RPS20, ZNF622, RPL28, DAP3, DNAJC21, RPL7, RPS16, RPS3, MRPL42, RPL7A, RPL23A, RPL19, MRPS17, RPL30, RPS15A, RPL18A, RPLP0, PNPT1, RACK1, MRPL22, MRPL55, DHX9, RRBP1, NAA10, RPL10A, RPL26* | 165 | 114 | -0.24 | -3.49 | 0.00 | 0.00 |
| GO:0044445 | Cytosolic part | *CCT5, RPLP2, EIF2AK3, CCT3, TCP1, SURF6, PFKP, RPL7L1, CCT4, CCT8, CCT7, RPL17, RPL14, RPLP1, RPL10, RPS4X, CCT2, BAIAP2, WASHC4, CCT6A, BAG6, RPS17, GET4, RPL37A, CTBP2, RPL23, PFKL, BORCS5, RPS14, RPL13A, RPS8, RPS18, RPL22, UCHL5, RPS23, RPL9, HOMER1, RPS11, RPL38, BORCS6, RRM1, RPS3A, RPS27, RPS27L, CYB5R3, RPS2, RPS13, RPL31, RPL11, RPL13, HTT, GEMIN5, RPL4, RPL18, RPS6, RPS9, HSPA8, RPS5, RPS12, RPL27A, ERC1, DHX33, RPL8, RSL24D1, RPL24, RPS26, RPSA, RPL34, NUFIP1, RPL29, ENO1, DBN1, RPS24, RPL27, RPL6, MMS19, RPS27, RPS29, RPL15, RPS10, RPS20, ZNF622, RPL28, PSMD14, RPL7, RPS16, RPS3, RPL7A, RPL23A, CIAO1, STX12, PSMD1, RPL19, SNAPIN, RPL30, RPS15A, RPL18A, PSMC6, RPLP0, RACK1* | 147 | 99 | -0.25 | -3.53 | 0.00 | 0.00 |
| GO:0005743 | Mitochondrial inner membrane | *MRPS6, MRPS27, CKMT1B, OPA1, MRPS28, PMPCB, TAMM41, TIMM21, MRPS34, MRPS18B, MRPS25, SDHA, TIMM44, SLC25A22, UQCRQ, NDUFS6, MRPS10, MAIP1, MRPS21, ATP5F1C, SLC25A15, SLC25A13, ATP5ME, UQCRC2, MRPS12, CYC1, MRPS15, MICU1, MICOS13, TIMM50, SFXN4, NDUFA4, MRPS23, SLC25A19, MRPS16, MRPL14, UQCRFS1, MRPS26, PARK7, NOA1, NDUFA11, SLC25A1, NDUFA6, MRPS7, MRPS22, SCO1, AIFM1, MRPL13, ATP5MD, COX15, MRPL17, SDHB, ATP5PD, LYN, NDUFA9, MRPL20, MRPS35, MRPS2, MRPL23, ATP5PB, TIMM23, MRPL48, TRAP1, PTCD3, MRPL39, BCS1L, EFHD1, MRPL37, MRPS9, NDUFA10, SLC25A12, MICU2, AFG3L2, UQCRC1, GPD2, COQ5, OXA1L, MRPS5, NDUFS1, PHB, COX4I1, TMEM70, NDUFAF4, MRPL44, SLC25A24, NDUFB10, YME1L1, MRPS30, NDUFS7, STOML2, MRPL11, MTCH2, HADHA, PISD, SLC25A6, NDUFS4, SHMT2, GOT2, LETM1, DAP3, CLPX, FECH, SLC25A3, SLC25A5, RPS3, SCO2, MRPL42, MRPS17, HIGD1A, IMMT, HADHB, UQCR10, COQ8B, ABCB7, MRPL22, MRPL55, CNP, ECSIT, ATP5MF, MRPL46, COQ8A, AGK, MRPS31, HSPD1, ATP5MG, SLC25A21, ALDH18A1, STAT3, CHCHD3, MRPL41, ATP5F1A, MRPL24, RCC1L, PMPCA, ERAL1* | 177 | 135 | -0.24 | -3.59 | 0.00 | 0.00 |
| GO:1990204 | Oxidoreductase complex | *PMPCB, BCKDHB, SDHA, UQCRQ, NDUFS6, BCKDHA, UQCRC2, PDK1, DHTKD1, CYC1, NDUFA4, PDK2, UQCRFS1, PARK7, NDUFA11, NDUFA6, RRM1, DBT, SDHB, NDUFA9, BCKDK, DLD, BCS1L, KAT2A, NDUFA10, PDHA1, PDHB, UQCRC1, GPD2, OGDH, NDUFS1, NDUFB10, DLAT, NDUFS7, P4HB* | 44 | 35 | -0.48 | -3.78 | 0.00 | 0.00 |
| hsa03010 | Ribosome | *MRPS6, RPLP2, RPL17, RPL14, RPLP1, RPL10, RPS4X, MRPS10, MRPS21, MRPS12, MRPS15, RPS17, RPL37A, RPL23, RPS14, MRPS16, MRPL14, RPL13A, RPS8, RPS18, RPL22, RPS23, RPL9, RPS11, RPL38, MRPS7, MRPL13, RPS3A, MRPL17, RPS27, RPS27L, MRPL20, RPS2, MRPS2, MRPL23, RPS13, RPL31, RPL11, RPL13, RPL4, RPL18, RPS6, RPS9, RPS5, MRPS9, RPS12, RPL27A, MRPS5, RPL8, RSL24D1, RPL24, RPS26, RPSA, RPL34, RPL29, RPS24, RPL27, RPL6, RPS27, RPS29, MRPL11, RPL15, RPS10, RPS20, RPL28, RPL7, RPS16, RPS3, RPL7A, RPL23A, RPL19, MRPS17, RPL30, RPS15A, RPL18A, RPLP0, MRPL22* | 100 | 76 | -0.33 | -3.83 | 0.00 | 0.00 |
| GO:0003735 | Structural constituent of ribosome | *MRPS6, RPLP2, MRPS34,MRPS18B, MRPS25, RPL7L1, RPL17, RPL14, RPLP1, RPL10, RPS4X, MRPS21, MRPS12, MRPS15, RPS17, RPL37A, RPL23, MRPS23, RPS14, MRPS16, MRPL14, RPL13A, RPS8, RPS18, RPL22, RPS23, RPL9, RPS11, RPL38, MRPS7, MRPS22, MRPL13, RPS3A, MRPL17, RPS27, RPS27L, MRPL20, MRPS35, RPS2, MRPS2, MRPL23, RPS13, RPL31, RPL11, RPL13, RPL4, RPL18, RPS6, RPS9, RPS5, MRPL37, MRPS9, RPS12, RPL27A, MRPS5, RPL8, RSL24D1, RPL24, RPS26, RPSA, RPL34, RPL29, RPS24, RPL27, RPL6, MRPS30, RPS27, RPS29, MRPL11, RPL15, RPS10, RPS20, RPL28, DAP3, RPL7, RPS16, RPS3, MRPL42, RPL7A, RPL23A, RPL19, MRPS17, RPL30, RPS15A, RPL18A, RPLP0, MRPL22, MRPL55* | 116 | 87 | -0.32 | -3.93 | 0.00 | 0.00 |
| R-HSA-72766 | Translation | *MRPS6, MRPS27, EIF3F, EIF3C, EIF3K, FARSB, MRPS28, RPLP2, TUFM, EIF3M, MRPS34, EIF3H, MRPS18B, MRPS25, EIF3L, FARS2, RPL17, AIMP2, RPL14, EIF3G, RPLP1, RPL10, RPS4X, MRPS10, EIF3I, EIF3E, QARS, MRPS21, MARS, FARSA, EIF3B, EEF2, TRMT112, EARS2, MRPS12, MRPS15, RPS17, RPL37A, RPL23, MRPS23, EIF3D, RPS14, MRPS16, MRPL14, RARS, SSR4, RPL13A, RPS8, NARS2, RPS18, RPL22, MRPS26, RPS23, RPL9, RPS11, RPL38, HARS, MRPS7, PARS2, MRPS22, MRPL13, RPS3A, EIF3A, MRPL17, CARS, SSR1, RPS27, RPS27L, MRPL20, RPS35, RPS2, SRPRB, RPN2, MRPS2, MRPL23, RPS13, MRPL48, RPL31, RPL11, RPL13, EIF2B4, PTCD3, MRPL39, RPL4, RPL18, VARS, RPS6, RPS9, LARS, RPS5, MRPL37, MRPS9, DARS2, RPS12, RPL27A, EIF4H, EIF4A2, EEF1B2, AIMP1, OXA1L, EIF2S3, MRPS5, RPL8, SEC61A1, EEF1E1, RPL24, RPS26, RPSA, RPL34, RPL29, DARS, MRPL44, RPS24, RPL27, TARS, RPL6, MRPS30, RPS27, RPS29, MRPL11, RPL15, RPS10, GFM2, RPS20, ETF1, RPL28, DAP3, SRP72, RPL7, RPS16, RPS3, EIF4B, MRPL42, YARS2, RPL7A, RPL23A, DDOST, IARS, RPL19, MRPS17, EIF2B3, SSR3, EIF1AX, RPL30, EIF4A1, RPS15A, EIF3J, RPL18A, RPLP0, MRPL22, EIF2S1, MRPL55* | 226 | 151 | -0.24 | -4.13 | 0.00 | 0.00 |
